# Contribution of adipocyte Na/K-ATPase α1/CD36 signaling induced exosome secretion in response to oxidized LDL

**DOI:** 10.3389/fcvm.2023.1046495

**Published:** 2023-04-27

**Authors:** Sneha S. Pillai, Duane G. Pereira, Jue Zhang, Wenxin Huang, Mirza Ahmar Beg, Darcy A. Knaack, Bruno de Souza Goncalves, Daisy Sahoo, Roy L. Silverstein, Joseph I. Shapiro, Komal Sodhi, Yiliang Chen

**Affiliations:** ^1^ Department of Surgery, Biomedical Sciences, and Medicine, Joan C. Edwards School of Medicine, Marshall University, Huntington, WV, United States; ^2^ Versiti Blood Research Institute, Milwaukee, WI, United States; ^3^ Department of Biochemistry, Medical College of Wisconsin, Milwaukee, WI, United States; ^4^ Department of Medicine, Medical College of Wisconsin, Milwaukee, WI, United States; ^5^ Department of Pharmacology & Toxicology, Medical College of Wisconsin, Milwaukee, WI, United States

**Keywords:** Na/K-ATPase, CD36, adipocyte, exosomes, oxLDL, mitochondria, ROS, macrophage

## Abstract

**Introduction:**

Adipose tissue constantly secretes adipokines and extracellular vesicles including exosomes to crosstalk with distinct tissues and organs for whole-body homeostasis. However, dysfunctional adipose tissue under chronic inflammatory conditions such as obesity, atherosclerosis, and diabetes shows pro-inflammatory phenotypes accompanied by oxidative stress and abnormal secretion. Nevertheless, molecular mechanisms of how adipocytes are stimulated to secrete exosomes under those conditions remain poorly understood.

**Methods:**

Mouse and human *in vitro* cell culture models were used for performing various cellular and molecular studies on adipocytes and macrophages. Statistical analysis was performed using Student's t-test (two-tailed, unpaired, and equal variance) for comparisons between two groups or ANOVA followed by Bonferroni's multiple comparison test for comparison among more than two groups.

**Results and discussion:**

In this work, we report that CD36, a scavenger receptor for oxidized LDL, formed a signaling complex with another membrane signal transducer Na/K-ATPase in adipocytes. The atherogenic oxidized LDL induced a pro-inflammatory response in *in vitro* differentiated mouse and human adipocytes and also stimulated the cells to secrete more exosomes. This was largely blocked by either CD36 knockdown using siRNA or pNaKtide, a peptide inhibitor of Na/K-ATPase signaling. These results showed a critical role of the CD36/Na/K-ATPase signaling complex in oxidized LDL-induced adipocyte exosome secretion. Moreover, by co-incubation of adipocyte-derived exosomes with macrophages, we demonstrated that oxidized LDL-induced adipocyte-derived exosomes promoted pro-atherogenic phenotypes in macrophages, including CD36 upregulation, IL-6 secretion, metabolic switch to glycolysis, and mitochondrial ROS production. Altogether, we show here a novel mechanism through which adipocytes increase exosome secretion in response to oxidized LDL and that the secreted exosomes can crosstalk with macrophages, which may contribute to atherogenesis.

## Introduction

Adipose tissue (AT), in addition to its lipid storage function, is well-recognized as an endocrine organ that can actively secrete hundreds of different bioactive molecules including lipids, adipokines, and extracellular vesicles (1–3). Recent advances in nanotechnology have facilitated the development of novel methods for the detection, identification, and quantification of exosomes, a specific class of small (30∼150 nm in diameter) extracellular vesicles secreted by cells (4). AT-derived exosomes (Ad-Exo) can travel in the circulation and modulate the function of distant organs including the liver, muscle, and brain in order to regulate appetite and whole-body metabolism (2). However, AT dysfunction is often associated with abnormal secretion including Ad-Exo that may contribute to metabolic diseases such as obesity and atherosclerosis (2, 5, 6).

AT consists of adipocytes, preadipocytes, vascular endothelial cells, fibroblasts, and a variety of immune cells such as macrophages, dendritic cells, and lymphocytes (7). In healthy AT adipocytes are the predominant cell type. Adipocytes constantly sense their microenvironment to determine nutritional status and respond by secreting a mixture of molecules (secretome) to communicate with surrounding cells such as endothelial cells and macrophages (8, 9) and dysfunction of both cell types play significant roles in atherosclerosis (10, 11). Among the adipocyte secretome, exosomes have recently attracted attention because they not only impact surrounding cells but also regulate the functions of organs and tissues at distant locations (12–14). Accumulating evidence has also implicated dysregulated exosomes in the atherogenesis (15). More specifically, Ad-Exo have been shown to aggravate atherosclerosis (16). However, it remains poorly understood how Ad-Exo are induced under atherogenic conditions.

Oxidized LDL (oxLDL) is an atherogenic lipoprotein produced from native LDL under conditions of oxidative stress (17). Distinct from unmodified native LDL, oxLDL has a high affinity for the cell surface scavenger receptor CD36 (18). We have shown that binding of oxLDL to CD36 on the macrophage cell surface leads to a profound change in macrophage gene expression, CD36 interactions with other membrane proteins such as the Na/K-ATPase (NKA) and tetraspanin CD9, intracellular signaling, mitochondrial reactive oxygen species (mtROS) production, and reorganization of lipid metabolism. All these events facilitate pro-atherogenic functions (19–23). Interestingly, in addition to its role as a scavenger receptor, CD36 also mediates cellular uptake of long-chain fatty acids (24). Therefore, it is not surprising that CD36 is also highly expressed in mature adipocytes, mediating fatty acid uptake for lipid storage (25). Besides CD36, we have shown that NKA, a ubiquitously expressed plasma membrane ion transporter and signal transducer (26), plays an important role in adipocyte ROS signaling and regulation (27). Treatment of adipocytes with oxLDL significantly alters gene expression related to oxidative stress and cellular metabolism, which is blocked by a specific NKA signaling inhibitor pNaKtide (27–30).

While oxLDL is known to induce exosome secretion from macrophages (31), whether and how oxLDL stimulates exosome release from adipocytes is not well explored. In this study, we examined the role of adipocyte CD36 and NKA in response to oxLDL and demonstrated that CD36 and the NKA α1 subunit form a signaling complex that promotes lipid accumulation, pro-inflammatory cytokine MCP-1 secretion, and exosome secretion. Moreover, we showed that oxLDL-induced Ad-Exo (oxLDL-Ad-Exo) promoted pro-atherogenic phenotypes in macrophages including CD36 upregulation, IL-6 secretion, enhanced glycolysis, and reduced oxidative phosphorylation accompanied by increased mtROS. The pro-atherogenic features of oxLDL-induced Ad-Exo were mostly attenuated by pNaKtide, implicating NKA signaling in adipocyte communication with macrophages under atherogenic conditions. Additionally, we demonstrated similar adipocyte/macrophage crosstalk through Ad-Exo using human mesenchymal stem cell (MSC)-derived adipocytes and human monocyte-derived macrophages, showing the relevance of our findings to human cells. Altogether, these studies reveal a novel adipocyte CD36/NKA-dependent mechanism by which adipocytes communicate with macrophages through Ad-Exo under atherogenic conditions.

## Materials and methods

### Preparation of oxidized LDL (oxLDL)

Human LDL were purchased from Lee BioSolutions (MO, USA). LDL were diluted and oxidized as previously described (18). Briefly, oxidation of LDL (0.5 mg/ml) by Cu^2+^ was performed by dialysis vs. 5 μM CuSO_4_ in PBS for 6 h at 37 °C. Oxidation was terminated by adding BHT (40 μM) and DTPA (100 μM). Then the solution was subjected to dialysis against PBS with DTPA (100 μM) to remove Cu^2+^. For quality control, LDL oxidation was confirmed by thiobarbituric acid reactive substances (TBARS) assay using a commercial kit (Abcam). The oxLDL stock solution (0.5 mg/ml) was put in a 15 ml tube with argon gas flushed above the solution and parafilm wrapped around the cap before the tube was stored in a 4 °C fridge until usage.

### Mouse cell differentiation to adipocytes and *in vitro* studies

For 3T3-L1 cell differentiation, frozen mouse pre-adipocytes (3T3-L1) were purchased from ATCC. After thawing, cells were suspended in Dulbecco's Modified Eagle Medium supplemented with 10% heat-inactivated fetal bovine serum and 1% antibiotic/antimycotic solution (Invitrogen, Carlsbad, CA, USA) and maintained at 37 °C in a 5% CO_2_ incubator. Upon attaining 60%–70% confluence, the medium was replaced with adipogenic medium (iXCells Biotechnologies, San Diego, CA, USA), and the cells were cultured in 6-well plates for an additional seven days. The cells were treated with pNaKtide (0.7 µM) and oxLDL (50µg/ml) on day 5 and day 6. On day 7, the conditioned medium was collected for exosome extraction and cytokine assays. The cells were harvested and solubilized in RPA buffer for RNA isolation.

For ear mesenchymal stem cells (EMSC) differentiation, EMSC were isolated from male or female WT or CD36^−/−^ mice (C57Bl/6J strain, 8–12 weeks of age) as previously described (32). Briefly, external ears were excised and enzymatically digested with 1.5 mg/mL collagenase IV at 37°C in a shaking incubator for 1 h. Following digestion, EMSC were plated and cultured at 37°C/5% CO_2_ in growth medium (DMEM/F12, 15% fetal bovine serum (FBS), 1% penicillin-streptomycin (P/S)) supplemented with 10–100 ng/mL recombinant fibroblast growth factor (FGF). At passage 3, EMSC were plated for experiments, allowed to reach maximum confluency, and incubated for an additional 48 h (approx. 4 days total) in a growth medium. At day 0, EMSC were cultured in a differentiation medium (growth medium, 5 µg/ml insulin, 3 µM rosiglitazone, 1 µM dexamethasone, 500 µM 3-isobutyl-1-methylxanthine (IBMX)). On day 2 post-differentiation, cells were switched to a maintenance medium (growth medium, 5 µg/ml insulin, 3 µM rosiglitazone) and fed every other day until day 9 post-differentiation. Experiments were performed in adipocytes between passage 3 and passage 5.

### Human adipose-derived mesenchymal stem cells differentiation to adipocytes and *in vitro* studies

Human adipose-derived MSC (hMSC) were purchased from ATCC and cultured in MSC Basal Media (ATCC) supplemented with MSC Growth Kit (ATCC) and maintained at 37°C in a 5% CO_2_ incubator. Upon attaining 60%–70% confluence, the medium was replaced with adipogenic medium and the cells were cultured for an additional 14 days to differentiate the cells into adipocytes. The medium was changed after 48 h. Treatments with pNaKtide (1 µM) and oxLDL (50 µg/ml) were performed on day 10 and day 12. On day 14, the conditioned medium was collected for exosome extraction and cytokine assay. The cells were harvested and homogenized in RIPA for protein isolation.

### Oil Red O staining for lipid accumulation

Cells were plated at a density of 0.05 × 10^6^ cells per well in a 24-well plate. Oil Red O power was purchased from Sigma-Aldrich. Oil Red O (0.21%) dissolved in isopropanol was used to stain neutral lipids in cells differentiated in the presence of the adipogenic media. Briefly, cells were washed and fixed in 10% formaldehyde for 15 min then incubated with Oil Red O solution for 20 min at room temperature, followed by rinsing with PBS. Oil Red O was eluted by the addition of 100% isopropanol for 10 min in a shaker and lipid accumulation was measured as the relative absorbance at OD = 490 nm.

### ^3^H-Palmitic acid uptake

Adipocytes were serum-starved for 3–4 h in DMEM/F12 supplemented with 0.5% fatty acid-free BSA. A fatty acid solution was prepared by conjugating [9,10-^3^H(N)]-palmitic acid with fatty acid-free bovine serum albumin at a 4:1 molar ratio for 5–10 min at room temperature. Following serum starvation, the palmitic acid solution was spiked into each well (2 µCi/well) and incubated for 5, 15, or 30 min. After 30 min, phloretin (final concentration: 200 µM) was added to each well to inhibit fatty acid uptake, the media was collected, and lipids were extracted in isopropanol for at least 24 h, dried down under N_2_, and resuspended in isopropanol. Radioactivity was measured in the media and isolated lipids using a liquid scintillation counter and percent fatty acid uptake was calculated.

### Measurement of MCP-1 and IL-6 Levels in cell culture media

Levels of human and mouse monocyte chemoattractant protein-1 (MCP-1) and interleukin-6 (IL-6) were determined in conditioned media using an Enzyme-Linked Immunosorbent Assay (ELISA) kit according to the manufacturer's protocol (Abcam).

### CD36 siRNA transfection

siRNA-mediated silencing of CD36 was performed using Viromer BLUE (OriGene) according to the manufacturer's protocol. The CD36 siRNA duplex sequences are:

5’- CUAUUGAAGGCUUACAUCCAAAUGA - 3’

| | | | | | | | | | | | | | | | | | | | | | | | |

3’- UGGAUAACUUCCGAAUGUAGGUUUACU - 5’

### RNA extraction and real-time PCR

RNA was extracted from the cells using RNeasy Protect Mini Kit (QIAGEN) according to manufacturer's instructions. The quality and quantity of the isolated RNA were evaluated using a NanoDrop Analyzer (Thermo Scientific). RNA was transcribed to cDNA using RevertAid RT kit (Thermo Scientific) and qRT-PCR reactions were performed in triplicate using SYBR Green PCR Master Mix on a 7,500 Fast Real-Time PCR System (Applied Biosystems). Specific predesigned mouse specific primers (IDT DNA Technologies) were used for the amplification. The primers used include TFAM (Transcription Factor A, Mitochondrial), NRF-1 (Nuclear respiratory factor 1) and MFN1 (Mitofusin 1). GAPDH was used as an endogenous control. The comparative threshold cycle method (ΔΔCt) was used to calculate the fold amplification. The sequences of the RT-PCR primers used are:

TFAM- TTT CCA AGC CTC ATT TAC AAG C

AAA CCA AAA AGA CCT CGT TCA G

MFN1- CCG CTC ATT CAC CTT ATG GA

 GCC TTG ATG CTG ATG TCT TTG

NRF1- TGA GAT GCA GAG TAC AAT CGC

 CCG AAA GAG ACA GCA GAC AC

GAPDH- AATGGTGAAGGTCGGTGTG

 GTGGAGTCATACTGGAACATGTAG

### Total exosome isolation from cell culture conditioned medium

Exosomes were isolated from adipocyte conditioned medium using Total Exosome Isolation (from cell culture media) reagent (Invitrogen) according to the manufacturer's instructions. Briefly, the conditioned medium was centrifuged at 2,000 x g for 10 min to remove cells and cell debris, followed by another round of centrifugation at 4,500 x g for 30 min to remove apoptotic bodies. The resulting cell-free media was mixed with 0.5 volumes of the Total Exosome Isolation reagent and incubated overnight at 4°C. After incubation, the samples were centrifuged at 10,000 x g for 1 h at 4°C. Exosomes contained in the pellet were stored at −80°C for further analysis.

To treat macrophages, exosomes isolated from each well of adipocyte conditioned medium were resuspended in 1 ml of culture medium (RPMI media plus 10% FBS), which was used to incubate with each well of macrophages.

### Nanoparticle tracking analysis

Nanoparticle tracking analysis (NTA) was performed by Nanomedicines Characterization Core Facility (NCore), Center for Nanotechnology in Drug Delivery, NC using a NanoSight NS500 nanoparticle analyzer (Malvern). The average particle (size between 30∼150 nm) number of each experimental group was calculated using the data sheet generated from NTA 3.4 Build 3.4.4 software.

### Electron microscopy

Isolated exosomes were fixed with 2% glutaraldehyde plus 4% paraformaldehyde in 0.1M sodium cacodylate buffer (pH 7.4) for 1 h, washed in the 0.1M buffer 3 × 5 min. Exosomes post fixation were examined using a Hitachi H600 transmission electron microscopy. Images were captured using a Hamamatsu CCD camera and processed using an AMT image Capture Engine Software version 602.571. Particles larger than 30 nm were counted in each randomly taken images.

### Western blot analysis for exosomal protein characterization and Nrf-1 expression

The cell pellet and exosomes were homogenized in RIPA buffer. The homogenates were centrifuged, the supernatant was isolated, and immunoblotting was performed using exosomal positive and negative markers, including anti-CD63 (Abcam), anti-TSG101 (Abcam) and anti-β-actin (Cell Signaling Technology) to confirm the success of exosome extraction. The conditioned medium recovered after the exosome isolation procedure was used as a negative control. Immunoblot analysis was performed for Nrf-1 (Abcam) and band densities were normalized to GAPDH (Millipore Sigma).

### Immunoprecipitation and immunoblot assays on adipocyte lysates

For co-immunoprecipitation of CD36 and NKA α1, adipocytes were lysed in CelLytic Lysis Reagent (Sigma) with protease inhibitor cocktail (Roche) and phosphatase inhibitors (Sigma). Cell lysates were pre-cleared with agarose beads (Life Technologies) for 1 h at 4°C. Cleared supernatant with 1 mg protein was incubated with 2 μg of anti-CD36 IgA (Thermo Scientific) or 2 μg of non-specific IgA as negative control for 2 h at room temperature. Then agarose beads were added and incubated overnight at 4°C. Beads were extensively washed with the lysis reagent and boiled in SDS-PAGE loading buffer, and the bound proteins were analyzed by immunoblots using anti-NKA α1 (Developmental Studies Hybridoma Bank). The membranes were striped and re-probed with anti-CD36 (Novus). Total cell lysates were also probed with anti-NKA α1 and anti-CD36 as input control.

### Isolation of peritoneal murine macrophages and *in vitro* studies

Mice were injected with 1 ml 4% thioglycollate intraperitoneally and 4 days later were sacrificed with CO_2_. Peritoneal cavities were flushed and macrophages were then suspended in 10 ml PBS pre-warmed to 37°C, counted, and centrifuged at 250 x g for 5 min. Cells were re-suspended in the culture media and seeded into culture dishes for further analysis. Freshly isolated murine peritoneal macrophages were cultured in RPMI media (Gibco by Life Technologies) supplemented with 10% FBS, 100U/ml penicillin, and 100 μg/ml streptomycin (Sigma) in 6-well plates at 37°C in a humidified incubator with 5% CO_2_. All subsequent treatments were conducted in RPMI media in the presence of 10% FBS.

### *In vitro* studies with human monocyte-derived macrophages

Human monocytes were isolated from buffy coats and differentiated into macrophages by incubation in X-ViVo 10 hematopoietic media (Lonza) supplemented with 5% human serum (Sigma), 100U/ml penicillin and 100 μg/ml streptomycin (Sigma) at 37°C in a humidified incubator with 5% CO_2_ for 5 days. All following treatments were conducted in X-ViVo 10 media in the presence of 5% human serum.

### Macrophage energetics

Macrophages were seeded into the specialized XF96 cell culture microplate (Seahorse Bioscience), about at a density of 50,000–80,000 cells/well. Cells were exposed to Ad-Exo for 24 h and then oxygen consumption rate (OCR) and extracellular acidification rate (ECAR) were measured using the Seahorse Bioscience Extracellular Flux Analyzer (Agilent) as previously described (22). Both Mito Stress test and Glycolytic Stress test were conducted. Cells were then lysed in RIPA buffer and subjected to Bradford protein assay (Bio-Rad). OCR and ECAR values were normalized to protein content.

### *In-Situ* proximity ligation assay (PLA)

The PLA assay was conducted as previously reported (33). Briefly, WT adipocytes or CD36 knockout (CD36^−/−^) adipocytes (negative control) were plated in 6-well plates with coverslips and fixed in 4% paraformaldehyde for 15 min at room temperature. The cells were permeabilized with 0.2% Triton X-100 in PBS for 10 min and incubated with anti-CD36 and anti-NKA α1 for 1 h. Then the oligonucleotide-labelled PLA probes were added. Samples were mounted with the Duolink mounting medium and PLA images were acquired using a Leica laser-scanning confocal microscope.

### Immunofluorescence and flow cytometry

Mouse peritoneal macrophages were analyzed using the anti-CD36 APC (HM36; 1:500) from Biolegend (San Diego, CA). Nonspecific binding of antibodies was blocked using Fc-Block (Biolegend 93, 1:100). All flow cytometry experiments were performed with a BD LSR II instrument using FACSDiva software with optimal compensation and gain settings determined for each experiment based on unstained and single-color stained samples. Live cells were gated based on cell side and forward scatter. Doublets were excluded based on FSC-A vs. FSC-H plots.

FlowJo software 10.8.1 (Tree Star, OR) was used to analyze the data. To assess mitochondrial ROS cells were seeded into 36 mm cell culture dishes at 1.5 × 10^6^ cells/dish and incubated in RPMI1640/10% FBS in the presence of 5 μM MitoNeoD (University of Glasgow) for 15 min at 37°C as previously described (22). At the end of incubation, cells were lifted by scraping and suspended in 200 μl PBS with 5%FBS and immediately subjected to flow cytometry analysis.

### Statistical analysis

Data are presented as means ± SE of at least 3 independent experiments. Statistical analysis was performed using Student's t-test (two-tailed, unpaired, and equal variance) for comparisons between two groups or ANOVA followed by Bonferroni's multiple comparison test for comparison among more than two groups. All statistical analyses were performed using GraphPad Prism 9 software. Statistical significance was accepted at *P* < 0.05.

## Results

### oxLDL promotes metabolic changes and pro-inflammatory responses in 3T3-L1 adipocytes, which are attenuated by pNaKtide

**Figure 1:**
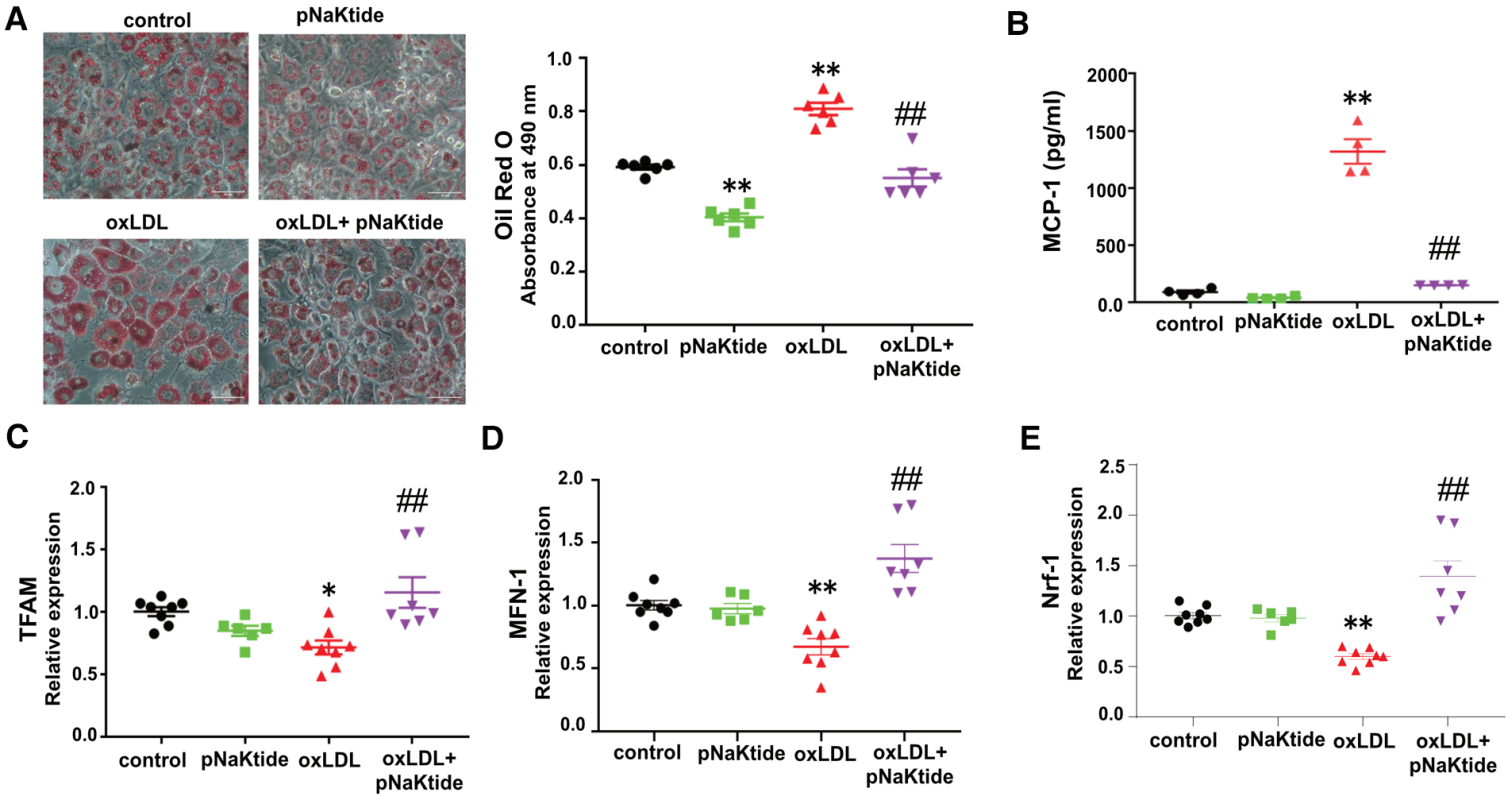
The effect of oxLDL treatment on lipid accumulation, inflammation, and mitochondrial dysfunction in 3T3-L1 murine adipocytes. Differentiated 3T3-L1 adipocytes were treated with 50 μg/ml oxLDL and/or 0.7 μM pNaKtide every 24 h for 2 days. (**A**) Representative images and quantitative data of lipid accumulation measured as relative absorbance of Oil Red O stain. Images were taken with a 20 × objective lens (*n* = 6). Scale bar: 50 μm. (**B**) ELISA for the quantitative analysis of pro-inflammatory cytokine MCP-1 (*n* = 4). (**C–E**) RT-PCR analysis for the relative mRNA expressions of TFAM, MFN-1, and Nrf-1 (*n* = 6–8). Values represent mean ± SEM. * *p* < 0.05 vs. control, ** *p* < 0.01 vs. control, ## *p* < 0.01 vs. oxLDL.

We showed previously that oxLDL-induced lipid accumulation in macrophages was dependent on CD36/NKA-mediated oxLDL uptake (21). To study the effect of oxLDL on adipocytes, 3T3-L1 murine adipocytes were subjected to Oil Red O staining after treatment with oxLDL in the presence and absence of pNaKtide. oxLDL increased lipid accumulation by ∼37% compared to control cells (Figure 1A). Administration of pNaKtide significantly reduced cellular neutral lipid levels and blocked the effect of oxLDL on elevated lipid accumulation (Figure 1A). The lipid accumulation/inhibition effects by either oxLDL or pNaKtide were not due to their influence on adipogenesis because the differentiation marker GAPDH (34) expression was not altered by either of the treatment (data not shown). As dysregulated lipid loading can lead to pro-inflammatory responses and mitochondrial dysfunction (35), we evaluated pro-inflammatory and mitochondrial markers in adipocytes exposed to oxLDL. MCP-1 levels were significantly increased in a conditioned medium of oxLDL-treated cells, compared to control cells (Figure 1B) and this was largely suppressed by pNaKtide (Figure 1B). Moreover, mRNA levels of mitochondrial marker genes TFAM, MFN-1, and NRF-1 were significantly down-regulated by oxLDL, and this too was reversed by pNaKtide co-treatment (Figure 1C–E). The results were further confirmed by the protein expression of Nrf-1 (Supplementary Material Figure S2). There were no significant changes in the protein expression of Nrf-1 in Control and oxLDL-treated groups, as shown in the previous report (36). However oxLDL + pNaKtide group showed a significant increase in Nrf-1 protein expression when compared to oxLDL alone. These results further confirmed the antioxidant protection offered by pNaKtide during the stress environment under oxLDL treatment.

### oxLDL stimulates Ad-Exo secretion from 3T3-L1 murine adipocytes, which is blocked by pNaKtide

**Figure 2:**
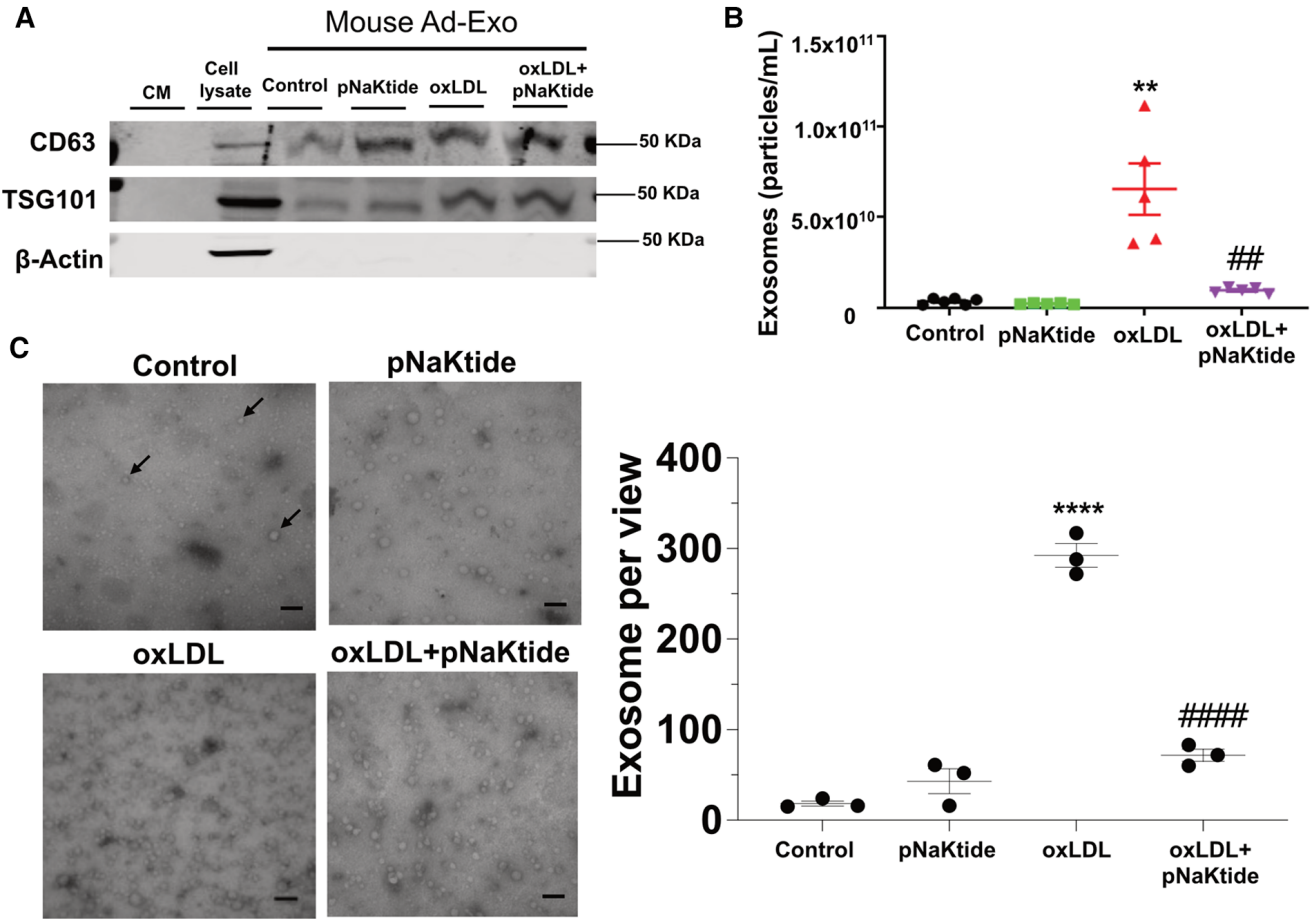
Characterization of the Ad-Exo isolated from 3T3-L1 murine adipocytes. (**A**) Immunoblot analysis of exosomal positive (CD63 and TSG101) and negative (β-actin) markers (*n* = 3). (**B**) NTA for the quantification of exosome particle number (*n* = 5–6). (**C**) Transmission electron microscopic images of the isolated exosomes. Black arrows point to the exosomes. Scale bar: 100 nm. The exosome number in each view was counted for each condition and shown in the bar graphs on the right. Values represent mean ± SEM. **p < 0.01, ****p < 0.0001 vs. control; ## *p* < 0.01, ####*p* < 0.0001 vs. oxLDL.

To test the effect of oxLDL on Ad-Exo secretion, we isolated and characterized exosomes from the conditioned media (CM) of 3T3-L1 adipocytes by size, structure, number and antigen expression. The specific exosome markers CD63 and TSG101 were detected in the isolated exosomes while the cytoplasmic marker β-actin was not seen, indicated high purity of the samples (Figure 2A). Nanoparticle tracking analysis (NTA) showed that oxLDL treatment induced adipocytes to secrete 18-fold more Ad-Exo than those secreted by control cells (Figure 2B). However, pNaKtide co-treatment significantly reduced oxLDL-induced exosome release to near baseline levels (Figure 2B). To corroborate the above findings, electron microscopic imaging studies were performed and showed typical exosome morphology and confirmed the presence of significantly more Ad-Exo in conditioned media from oxLDL-treated adipocytes compared to control cells (Figure 2C). Similar to the NTA results, the number of Ad-Exo in the conditioned medium was reduced when cells were co-treated by pNaKtide and oxLDL (Figure 2C).

### CD36 interacts with NKA α1 in adipocytes and is responsible for oxLDL-induced Ad-Exo secretion

**Figure 3:**
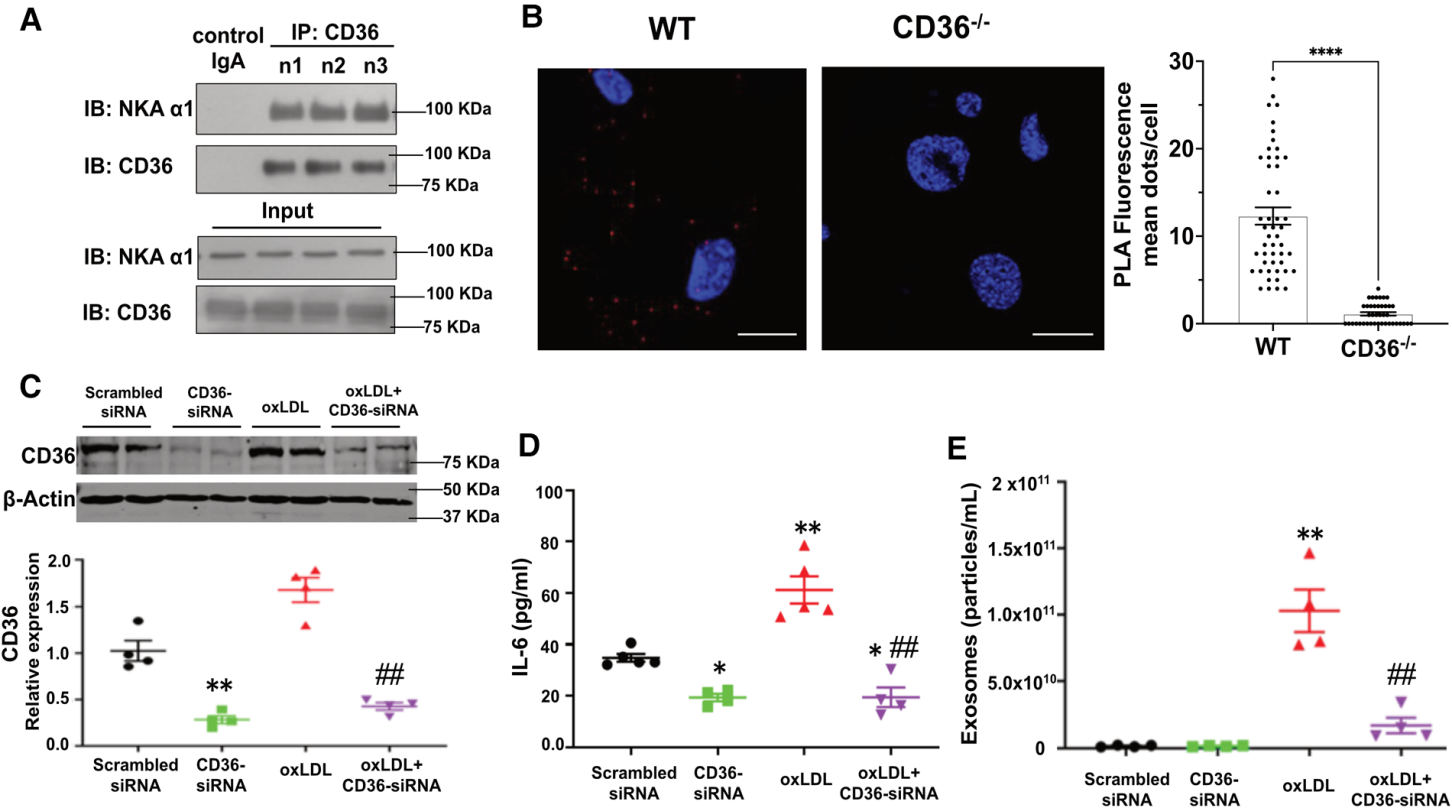
Formation of the CD36/NKA signaling complex in murine adipocytes. (**A**) WT murine adipocytes were lysed and immunoprecipitated with mouse anti-CD36 IgA (*n* = 3), followed by immunoblot for NKA α1 and CD36. Meanwhile, total cell lysates (input) were subjected to immunoblot for NKA α1 and CD36. An irrelevant mouse IgA was used as a negative control (first lane). Representative blot images are shown. (**B**) WT or CD36^−/−^ (negative control) murine adipocytes were subjected to *in-situ* PLA assay. PLA red fluorescence signals were captured by confocal imaging and representative images of each genotype are shown. Scale bar: 10 μm. PLA signals from five separate views of each genotype were counted and shown in the bar graphs. (**C**) 3T3 L1 murine adipocytes were transfected with scrambled siRNA (negative control) or CD36 siRNA and then treated with 50 μg/ml oxLDL for 24 h, followed by immunoblot for CD36. Representative blot images are shown and densitometry quantification is shown below (*n* = 4). (**D**) Same treatment as in (**C**), the cell culture medium was collected and subjected to ELISA assay for IL-6. Quantified data was shown (*n* = 5). (**E**) Same treatment as in (**C**), Ad-Exo were isolated from the cell culture medium and subjected to NTA. Quantified data was shown (*n* = 4). Values represent mean ± SEM. *p < 0.05, **p < 0.01 vs. scrambled siRNA, ##*p* < 0.01 vs. oxLDL.

To test whether the CD36/NKA signaling complex exists and functions in adipocytes, we utilized an *in vitro* murine adipocyte differentiation model using mesenchymal stem cells isolated from mouse external ear tissues (32). Successful adipocyte differentiation was demonstrated by positive Oil Red O staining (Supplementary Material Figure S1A) and upregulation of the adipocyte marker PPARγ (Supplementary Material Figure S1B). Stem cells from CD36 deficient (CD36^−/−^) mice showed normal adipocyte differentiation as detected by Oil Red O staining, but the differentiated cells had reduced palmitic acid uptake (Supplementary Material Figure S1C), consistent with the known role of CD36 in long-chain fatty acid uptake (25). We immunoprecipitated CD36 from differentiated cells using mouse anti-CD36 IgA followed by immunoblot with anti-NKA α1. NKA α1 co-precipitated with anti-CD36 from adipocyte cell lysates, while an irrelevant mouse IgA precipitated neither CD36 nor NKA α1 (Figure 3A). To confirm, we conducted an *in-situ* Proximity Ligation Assay (PLA), which demonstrates two proteins in close proximity (<40 nm) to each other (33). Consistently, we observed many positive PLA signals (bright red fluorescent dots) from WT adipocytes but very few from CD36^−/−^ adipocytes, further supporting a direct interaction between CD36 and NKA α1 (Figure 3B). Next, we tested whether CD36 expression was important for Ad-Exo secretion in response to oxLDL by specifically knocking down CD36 expression with siRNA before oxLDL treatment. As expected, CD36 siRNA led to a ∼70% reduction in CD36 expression in adipocytes. While oxLDL upregulated adipocyte CD36, a phenomenon also observed in macrophages (37), CD36 siRNA fully blocked the upregulation effect by oxLDL (Figure 3C). Knocking down adipocyte CD36 significantly also reduced oxLDL-stimulated IL-6 secretion (Figure 3D) and exosome secretion (Figure 3E). These results indicate an indispensable role of adipocyte CD36 in pro-inflammatory response and exosome secretion induced by oxLDL.

### oxLDL-Induced Ad-Exo promote a pro-atherogenic phenotype in macrophages

**Figure 4:**
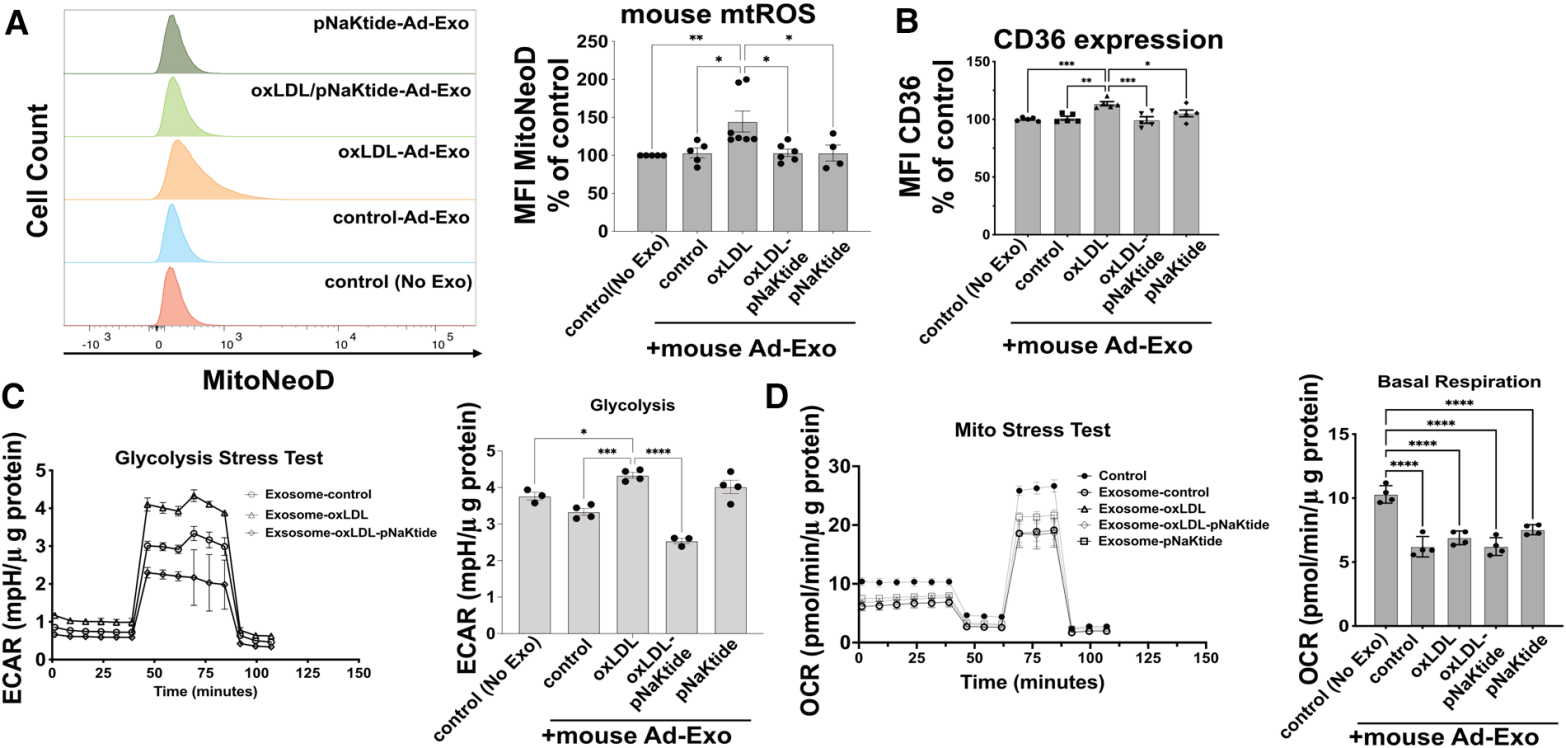
**oxLDL-Ad-Exo promote pro-atherogenic phenotypes in macrophages.** Ad-Exo were isolated from a conditioned medium of 3T3-L1 adipocytes subjected to 50 μg/ml oxLDL and/or 0.7 μM pNaKtide every 24 h for 2 days. For each condition, 1 ml of the conditioned medium was collected and Ad-Exo were isolated. Then isolated Ad-Exo were dissolved in a macrophage culture medium and incubated with WT murine peritoneal macrophages for 24 h. (**A**) Macrophage mtROS were assayed by MitoNeoD and signals were quantified by mean fluorescence intensity (MFI) of flow cytometry results. Histograms of different treatments are shown on the left and MFI bar graphs are shown on the right (*n* = 4–7). (**B**) Macrophage surface CD36 levels were quantified by MFI of flow cytometry results and are shown in the bar graphs (*n* = 5). (**C**) Glycolytic stress tests were conducted on treated macrophages. ECAR curves are shown on the left and maximum ECAR values are shown in the bar graphs on the right (*n* = 4). (**D**) Mito stress tests were conducted on treated macrophages. OCR curves are shown on the left and basal respiration values are shown in the bar graphs on the right (*n* = 4). Values represent mean ± SEM. *p < 0.05, **p < 0.01, ***p < 0.001, ****p < 0.0001.

To test the effect of Ad-Exo on macrophages, we isolated Ad-Exo from 3T3-L1 adipocyte conditioned medium and incubated them (using the doses as shown in Figure 2B) with murine peritoneal macrophages for 24 h. Using MitoNeoD, a cell-permeable probe specific for detecting mtROS (22, 38), we found that only Ad-Exo from oxLDL-treated adipocytes (oxLDL-Ad-Exo) significantly promoted mtROS production in macrophages with an approximate 50% increase in mean fluorescence (Figure 4A). Similarly, only oxLDL-Ad-Exo up-regulated macrophage surface CD36 (Figure 4B). We also tested the effect of Ad-Exo on macrophage bioenergetics and found that they significantly increased glycolysis (ECAR) (Figure 4C), while decreasing mitochondrial respiration (OCR) (Figure 4D), indicating that oxLDL-Ad-Exo induced a glycolytic switch in macrophages. pNaKtide co-treatment of adipocytes inhibited the above effects, except for mitochondrial respiration as Ad-Exo from all conditions downregulated this process (Figure 4D). Taken together, these data show that oxLDL-Ad-Exo promoted multiple phenotypic alterations in macrophages, all of which may facilitate atherogenesis (22).

### pNaKtide attenuates oxLDL-induced lipid accumulation, inflammation, exosome secretion and mitochondrial ROS in human MSC-derived adipocytes

**Figure 5:**
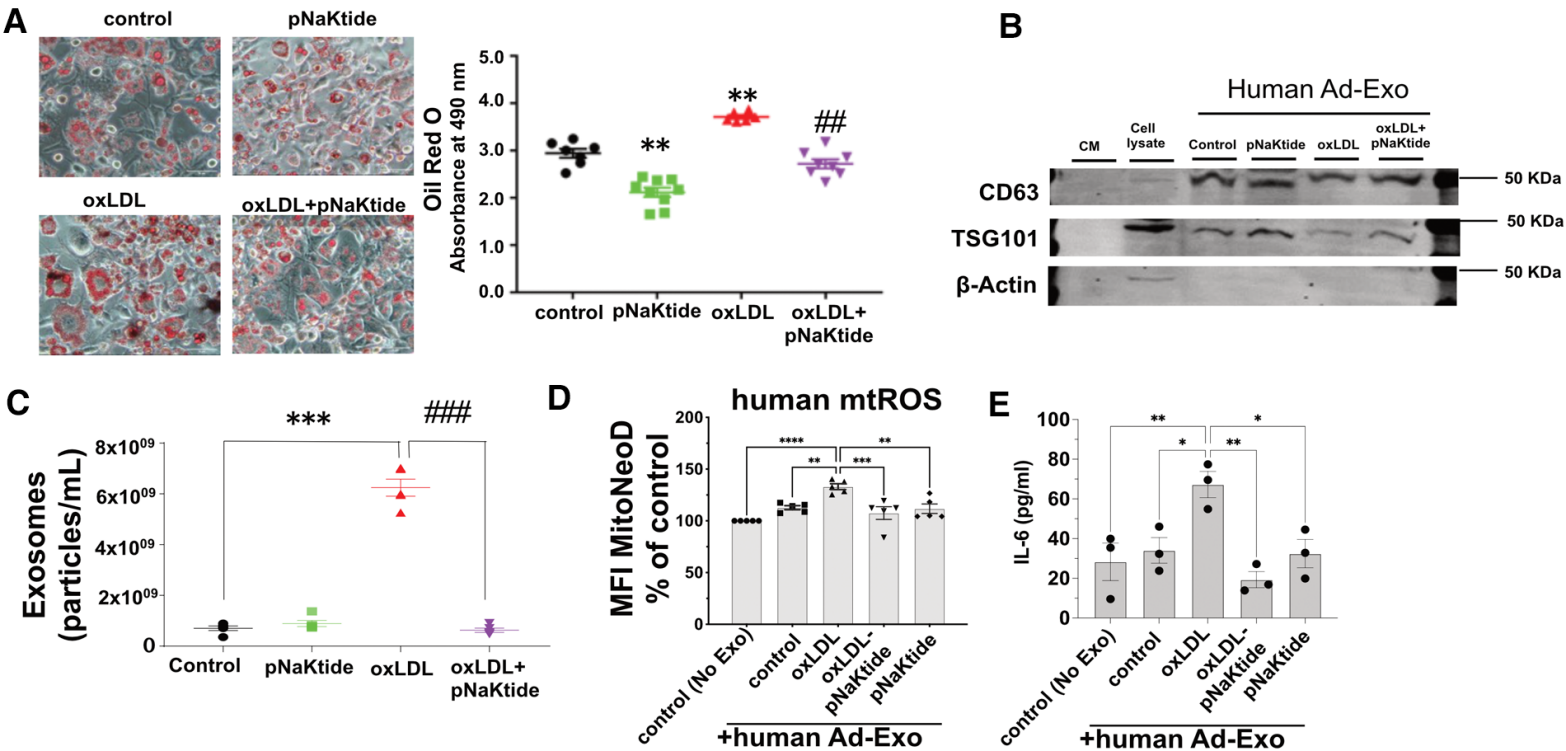
The effect of oxLDL treatment on lipid accumulation, inflammation, exosome secretion, and mitochondrial dysfunction in hMSC-derived adipocytes. (A) Representative images and quantitative data of lipid accumulation measured as relative absorbance of Oil Red O stain. Images were taken with a 20 × objective lens (*n* = 6-9)). (B) Immunoblot analysis of exosomal positive (CD63 and TSG101) and negative (β-actin) markers (*n* = 3) (C) NTA for the quantification of human exosome particle number (*n* = 5). *p < 0.05, **p < 0.01 vs. control, ##*p* < 0.01 vs. oxLDL. (D) Human monocyte-derived macrophages were treated with human Ad-Exo from the conditioned medium of human MSC-derived adipocytes for 24 h. Macrophage mtROS were assayed and shown in the bar graphs. (E) hMDM culture medium was subjected to ELISA assay for quantitative analysis of IL-6. **p < 0.01, ***p < 0.001, ****p < 0.0001 vs. oxLDL-Ad-Exo. Values represent mean ± SEM.

Oil Red O staining performed in hMSC-derived adipocytes showed similar results as those obtained for 3T3-L1 murine adipocytes. oxLDL-treated cells showed 26% increased lipid content as compared to control cells (Figure 5A) and reduced adipocyte lipid content to the control level with pNaKtide treatment. Human Ad-Exo isolated from a conditioned medium of hMSC-derived adipocytes expressed exosomal protein markers CD63 and TSG101 but not the cytoplasmic marker β-actin (Figure 5B). Moreover, NTA showed a significant 8.8-fold increase in exosome particle number from the conditioned medium of oxLDL-treated cells compared to the conditioned medium of control cells, which was blocked by pNaKtide co-treatment (Figure 5C). Co-incubating oxLDL-induced human Ad-Exo with human monocyte-derived macrophages (hMDM) led to a 33% elevation in hMDM mtROS, a phenomenon notobserved in human macrophages treated by Ad-Exo from other conditions (i.e., control, pNaKtide, oxLDL + pNaKtide) (Figure 5D). Culture media collected from hMDM 24 h after Ad-Exo treatment showed more than 2-fold increase in IL-6 secretion compared to control cells, while Ad-Exo isolated from oxLDL-treated adipocytes co-treated with pNaKtide did not stimulate IL-6 secretion (Figure 5E).

## Discussion

Here, we report that oxLDL induced metabolic changes, pro-inflammatory responses, and exosome secretion from mouse and human adipocytes. Moreover, oxLDL-induced Ad-Exo facilitated pro-atherogenic phenotypes in macrophages including upregulation of cell surface CD36, metabolic switch from mitochondrial oxidative phosphorylation to glycolysis, and mitochondrial ROS production (Figure 4). Mechanistically, oxLDL-stimulated Ad-Exo secretion was mediated by a signaling complex between the oxLDL receptor CD36 and its membrane signaling partner NKA (Figure 3). Either knocking down CD36 expression by siRNA or inhibiting NKA signaling by pNaKtide (28) blocked oxLDL-induced Ad-Exo secretion. Additionally, adipocyte treatment with pNaKtide significantly suppressed the pro-atherogenic features of oxLDL-induced Ad-Exo (Figures 4, 5).

Obesity is a risk factor for atherosclerosis and multiple mechanisms link obesity to atherosclerosis including elevated blood pressure, dysregulated blood glucose/lipid levels, and chronic inflammation (39). Many of these effects are mediated by secreted molecules/vesicles from the adipocytes. The adipocyte secretome not only regulates surrounding endothelial cells and immune cells but also travels in the circulation and directly impacts the functions of various cells at distant locations (12, 14, 39). Among the diversity of adipocyte-secreted molecules/vesicles, Ad-Exo have been reported to mediate pro-atherogenic effects *in vivo* through macrophages (6). Nevertheless, molecular mechanisms underlying Ad-Exo production and secretion under atherogenic conditions remain poorly understood.

We previously reported that NKA amplified oxidant signaling in adipocytes and administration of pNaKtide alleviated oxidant stress in adipocytes and improved insulin sensitivity in mice on high fat diet (26). Interestingly, we found that oxLDL reduced the expression of TFAM, MFN-1, and Nrf-1 (Figure 1C–E), which are responsible for mitochondrial biosynthesis, DNA stability, and respiration (40–42). These downregulation effects by oxLDL were reversed by pNaKtide, which suggests that oxLDL interrupts adipocyte mitochondrial functions through NKA signaling. These findings further support a pro-oxidant role of NKA signaling by disrupting mitochondrial respiration and stability, potentially leading to more ROS production. Moreover, using next-generation RNA sequencing technology, we showed that oxLDL treatment lead to a profound alteration in adipocyte transcriptome including metabolic pathways, oxidant signaling and stress, and inflammatory signaling pathways, which was attenuated by pNaKtide co-treatment (27). As extracellular vesicle production and ROS signaling can affect each other (43), in this work, we tested the hypothesis that NKA signaling contributes to oxLDL-induced secretion of pro-atherogenic Ad-Exo. Our results support the hypothesis as we observed more than 18-fold elevation in Ad-Exo secretion from cells treated with oxLDL compared to control cells, and this was almost fully blocked by pNaKtide (Figure 2). Thus, we propose that oxLDL binds to CD36 on the adipocyte surface and may cause a conformational change in the CD36/NKA signaling complex, which subsequently relays signals intracellularly potentially through src-family kinases and downstream ROS generation (26). This model explains why pNaKtide, by mimicking an intracellular domain within the NKA α1 subunit and suppressing src-family kinase activity (28), is able to block the effects of extracellular oxLDL. Interestingly, we reported the presence of CD36/NKA signaling complex in other cell types including renal cells (33) and macrophages (21), which facilitated oxidative stress and pro-inflammatory phenotypes under atherogenic conditions. Our findings here have further indicated that CD36 and NKA coordinate in a variety of cell types to regulate cellular redox status as well as metabolism and inflammation. It will be highly interesting to further explore the crosstalk of their downstream signaling pathways in different cells and how they communicate leading to chronic inflammation and abnormal lipid metabolism during atherogenesis.

**Figure 6:**
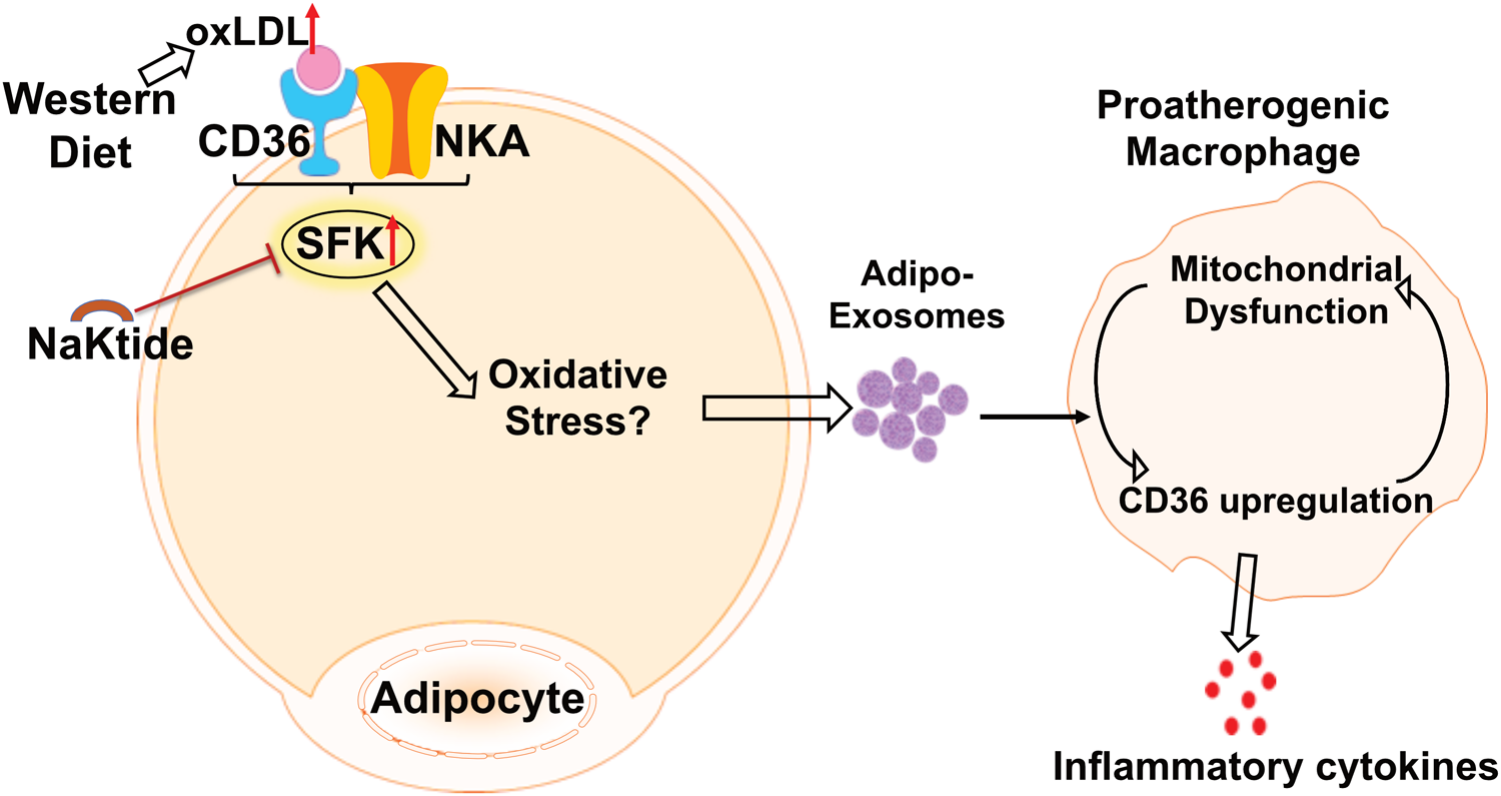
The schematic model of adipocyte CD36/NKA signaling that facilitate Ad-Exo secretion, which promote proatherogenic macrophage phenotypes.

Ad-Exo represent an important mechanism for adipose tissue communication with other cells/tissues and they may drive disease progression under pathological conditions (2). Consistent with this notion, we show here that oxLDL-induced Ad-Exo stimulated many pro-atherogenic phenotypes in macrophages (22). In addition, pNaKtide co-treatment of adipocytes largely abolished the pro-atherogenic features of Ad-Exo (Figure 4). As oxLDL significantly induced Ad-Exo number, which was largely prevented by pNaKtide (Figure 2), it is very likely that the high number of Ad-Exo induced by oxLDL is a major contributor to macrophage proatherogenic activation. However, it should be noted that the quality of the Ad-Exo (i.e., molecular composition of the Ad-Exo) may also change *via* different treatments to adipocytes. As an example, Ad-Exo contain microRNAs that are able to regulate macrophage polarization and inflammatory activation (13). Under various conditions, the Ad-Exo microRNA species may change, which subsequently impose distinct effects on macrophages and other cells. Alternatively, Ad-Exo could carry different proteins or lipid species to regulate cellular metabolism and functions (Figure 6). In addition to adipocyte-macrophage cross-talk, adipocytes may influence endothelial cell functions, which also play a critical role in the development of atherosclerosis (11). In fact, adipocyte-endothelium cross-talk has been widely recognized and studied in obesity (8), but the molecular mechanisms by which the two cell types regulate each other under atherogenic conditions remain poorly understood. Exosomes may represent one of the major intercellular communication mechanisms as they could facilitate exchange of intracellular content. Undoubtedly, further studies are required to explore the above possibilities for a better understanding of the molecular mechanisms of how adipocytes/adipose tissues communicate with various cell types under physiological and atherogenic conditions.

## Data Availability

The raw data supporting the conclusions of this article will be made available by the authors, without undue reservation.
