# Supplementary material for: Contribution of adipocyte Na/K-ATPase α1/CD36 signaling induced exosome secretion in response to oxidized LDL
Source: Front Cardiovasc Med. 2023 Apr 27;10:1046495. doi: 10.3389/fcvm.2023.1046495 (PMC10174328; doi:10.3389/fcvm.2023.1046495)
Supplement: Supplementary file 2 [file Datasheet2.zip › Figure 2/3T3L1 Exosome - WB/3T3L1 Exosomes - WB.pptx]

## Slide 1
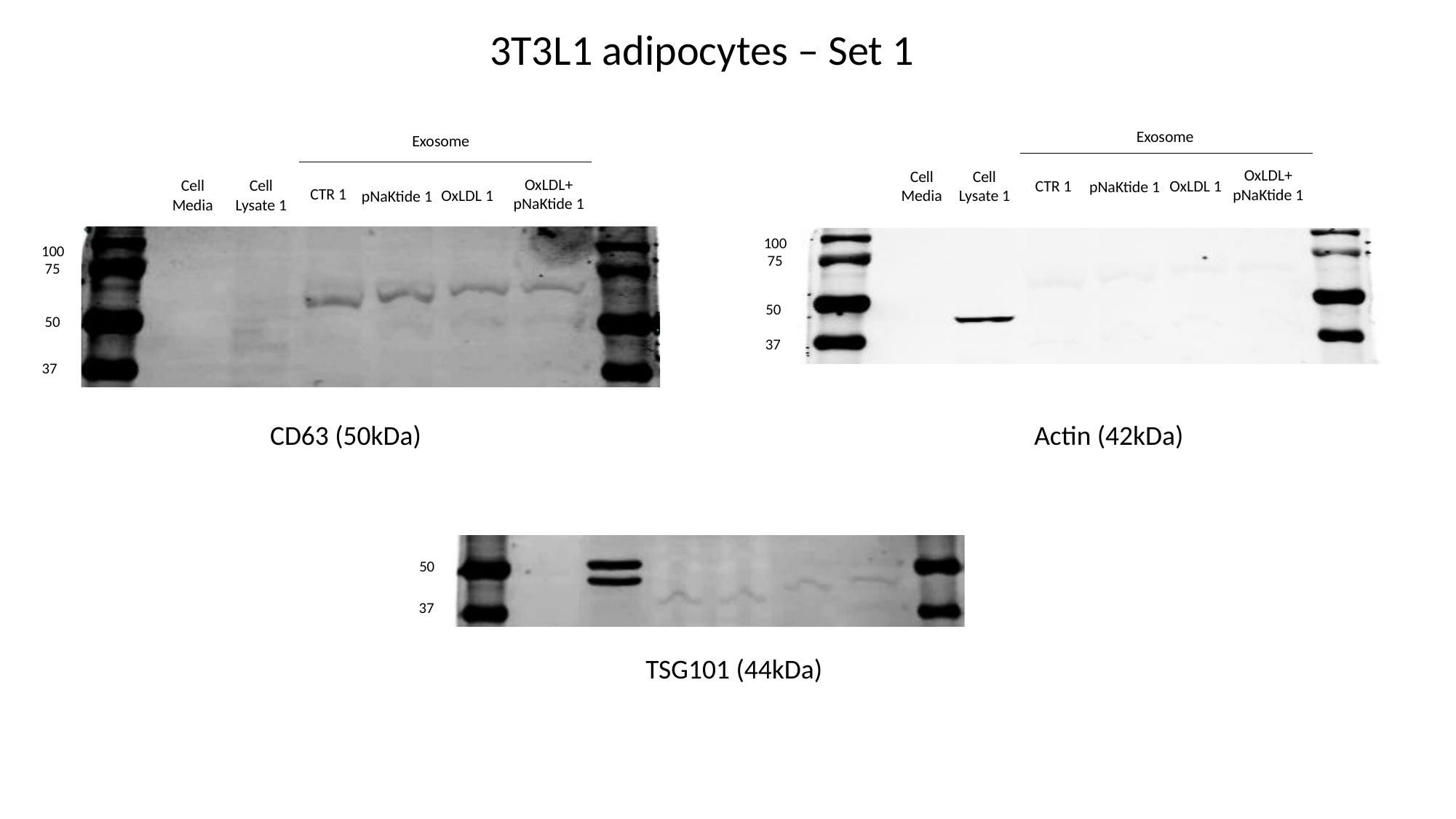

3T3L1 adipocytes – Set 1
Exosome
Exosome
OxLDL+
pNaKtide 1
Cell Media
Cell Lysate 1
OxLDL+
pNaKtide 1
Cell Media
Cell Lysate 1
CTR 1
OxLDL 1
pNaKtide 1
CTR 1
OxLDL 1
pNaKtide 1
100
100
75
75
50
50
37
37
CD63 (50kDa)
Actin (42kDa)
50
37
TSG101 (44kDa)

## Slide 2
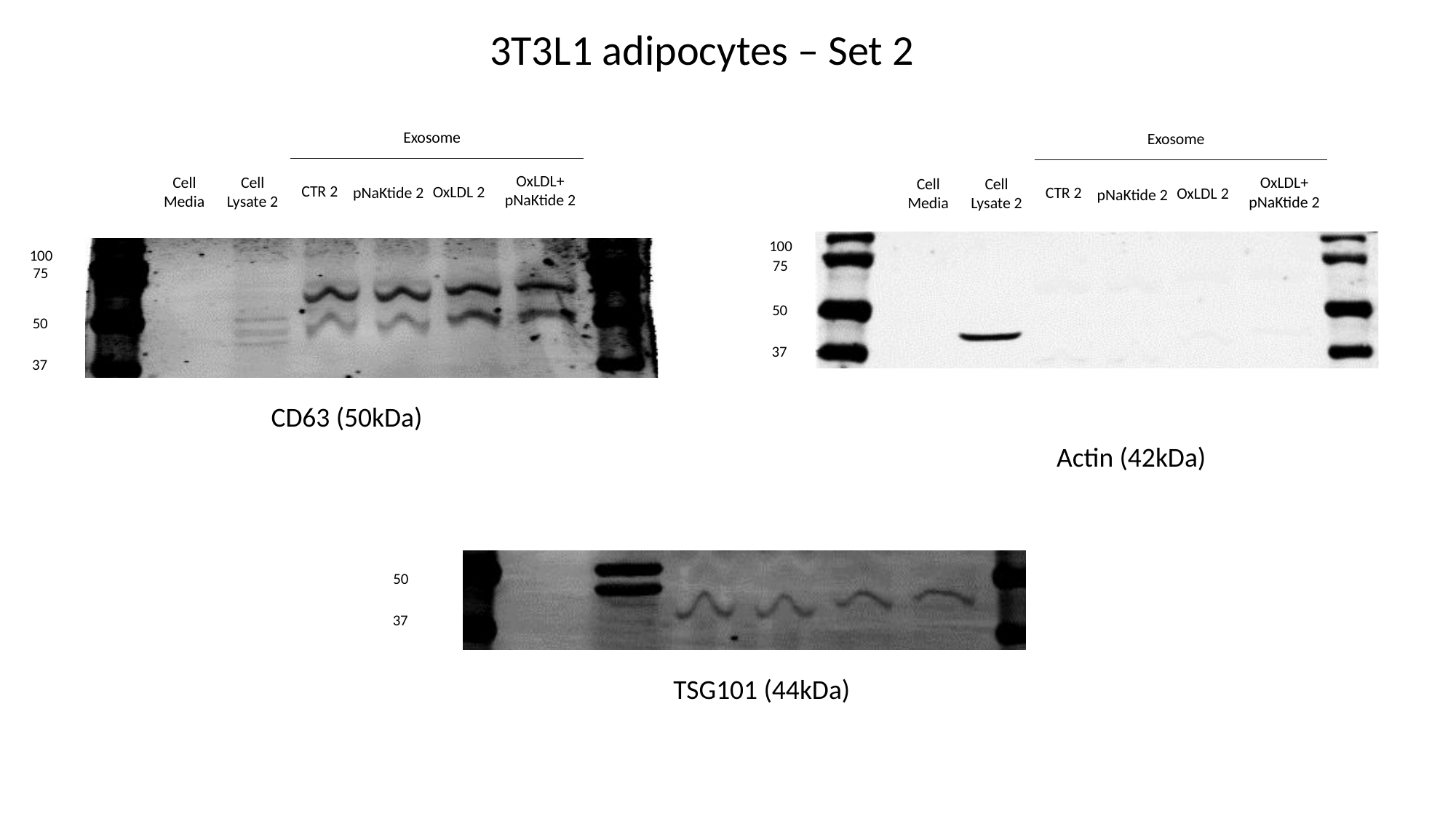

3T3L1 adipocytes – Set 2
Exosome
Exosome
OxLDL+
pNaKtide 2
Cell Media
Cell Lysate 2
OxLDL+
pNaKtide 2
Cell Media
Cell Lysate 2
CTR 2
OxLDL 2
CTR 2
pNaKtide 2
OxLDL 2
pNaKtide 2
100
100
75
75
50
50
37
37
CD63 (50kDa)
Actin (42kDa)
50
37
TSG101 (44kDa)

## Slide 3
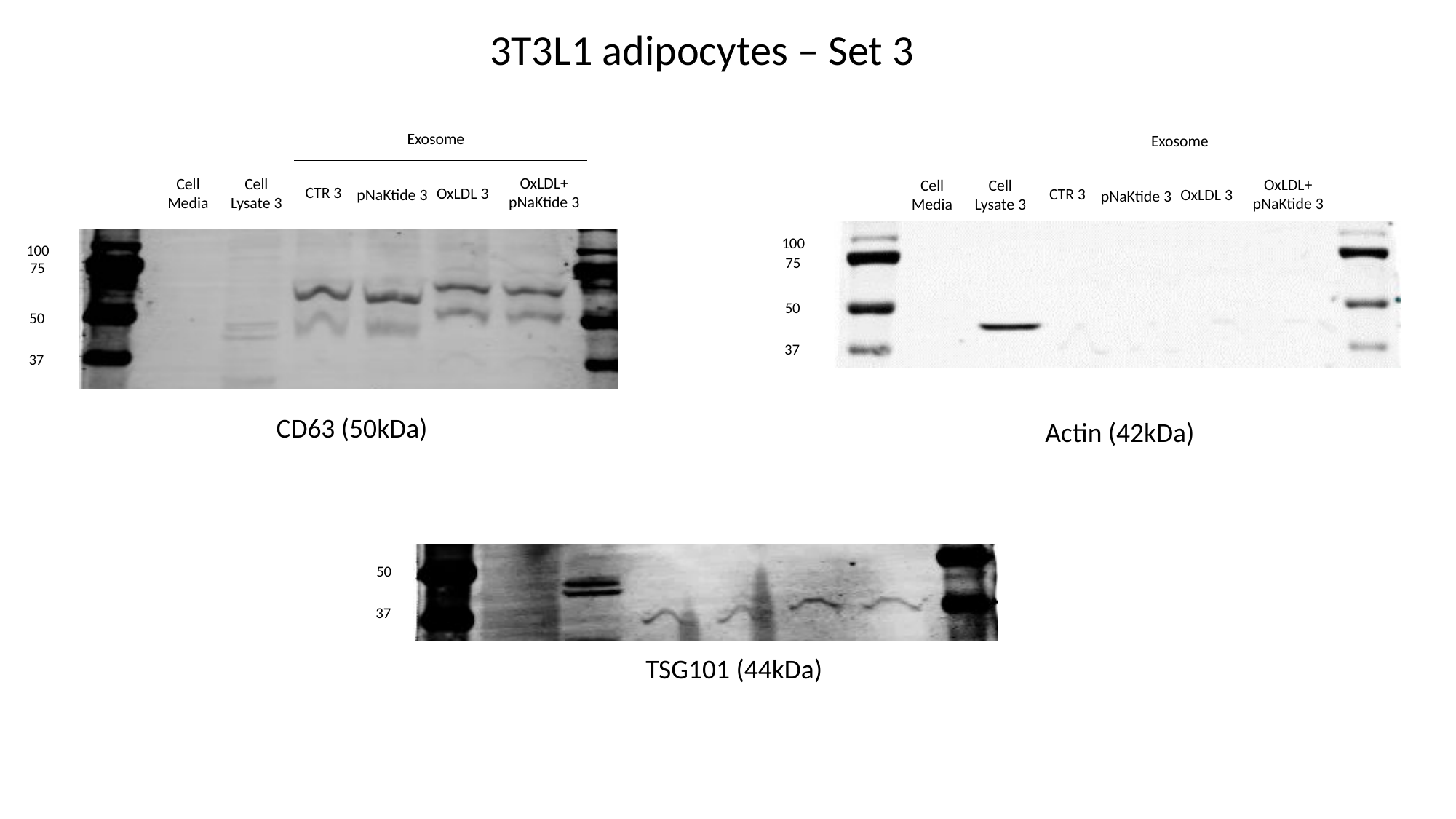

3T3L1 adipocytes – Set 3
Exosome
Exosome
OxLDL+
pNaKtide 3
Cell Media
Cell Lysate 3
OxLDL+
pNaKtide 3
Cell Media
Cell Lysate 3
CTR 3
OxLDL 3
CTR 3
pNaKtide 3
OxLDL 3
pNaKtide 3
100
100
75
75
50
50
37
37
CD63 (50kDa)
Actin (42kDa)
50
37
TSG101 (44kDa)

## Slide 4
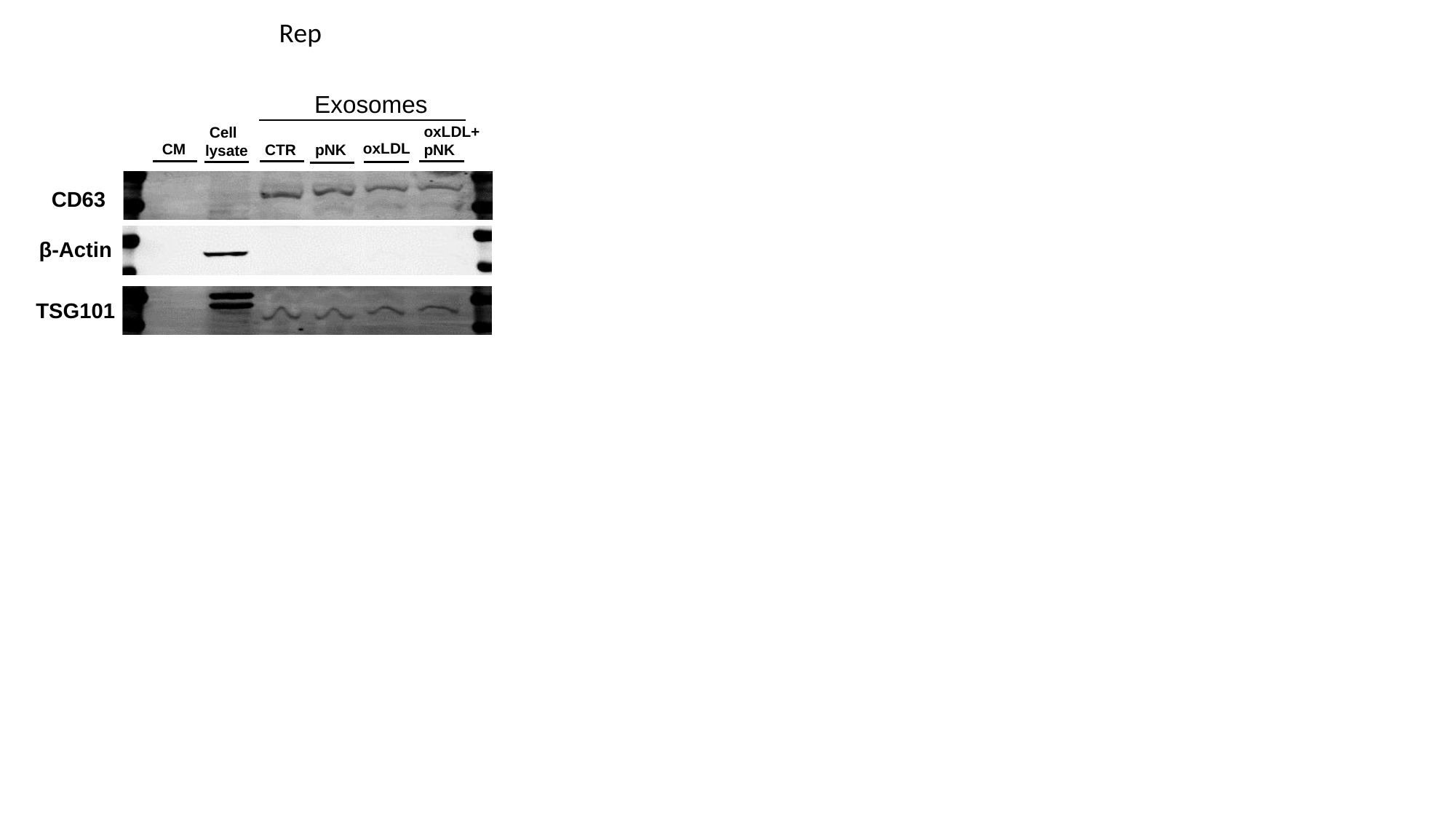

Rep
Exosomes
oxLDL+pNK
 Cell
lysate
oxLDL
CM
pNK
CTR
CD63
β-Actin
TSG101
