# Supplementary material for: Contribution of adipocyte Na/K-ATPase α1/CD36 signaling induced exosome secretion in response to oxidized LDL
Source: Front Cardiovasc Med. 2023 Apr 27;10:1046495. doi: 10.3389/fcvm.2023.1046495 (PMC10174328; doi:10.3389/fcvm.2023.1046495)
Supplement: Supplementary file 2 [file Datasheet2.zip › Figure 2/NTA 3T3 adipo exosomes/oxLDL Exosome concentration nanotracking.pptx]

## Slide 1
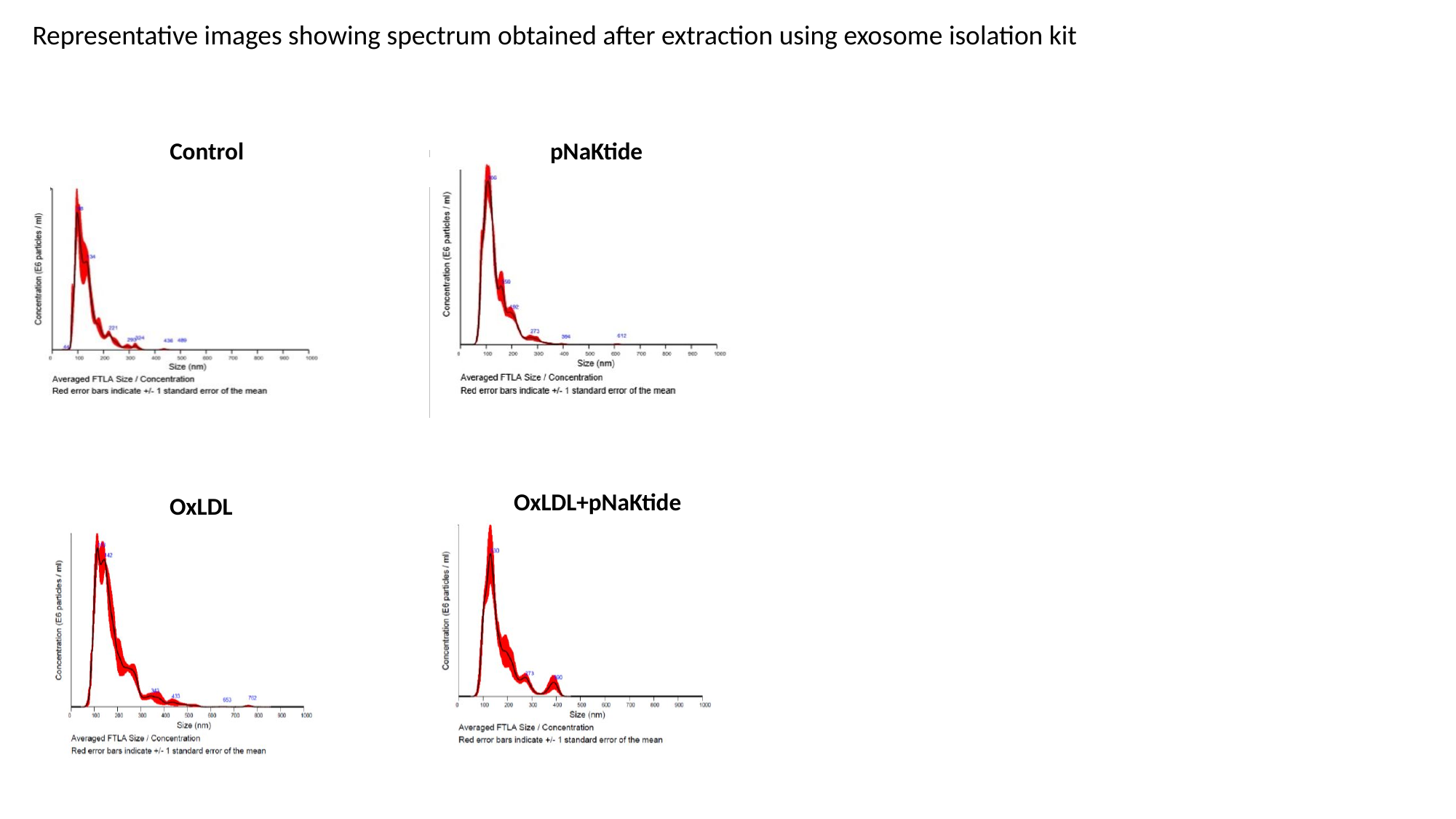

Representative images showing spectrum obtained after extraction using exosome isolation kit
Control
pNaKtide
OxLDL+pNaKtide
OxLDL
