# Supplementary figures and images for: Contribution of adipocyte Na/K-ATPase α1/CD36 signaling induced exosome secretion in response to oxidized LDL
Source: Front Cardiovasc Med. 2023 Apr 27;10:1046495. doi: 10.3389/fcvm.2023.1046495 (PMC10174328; doi:10.3389/fcvm.2023.1046495)

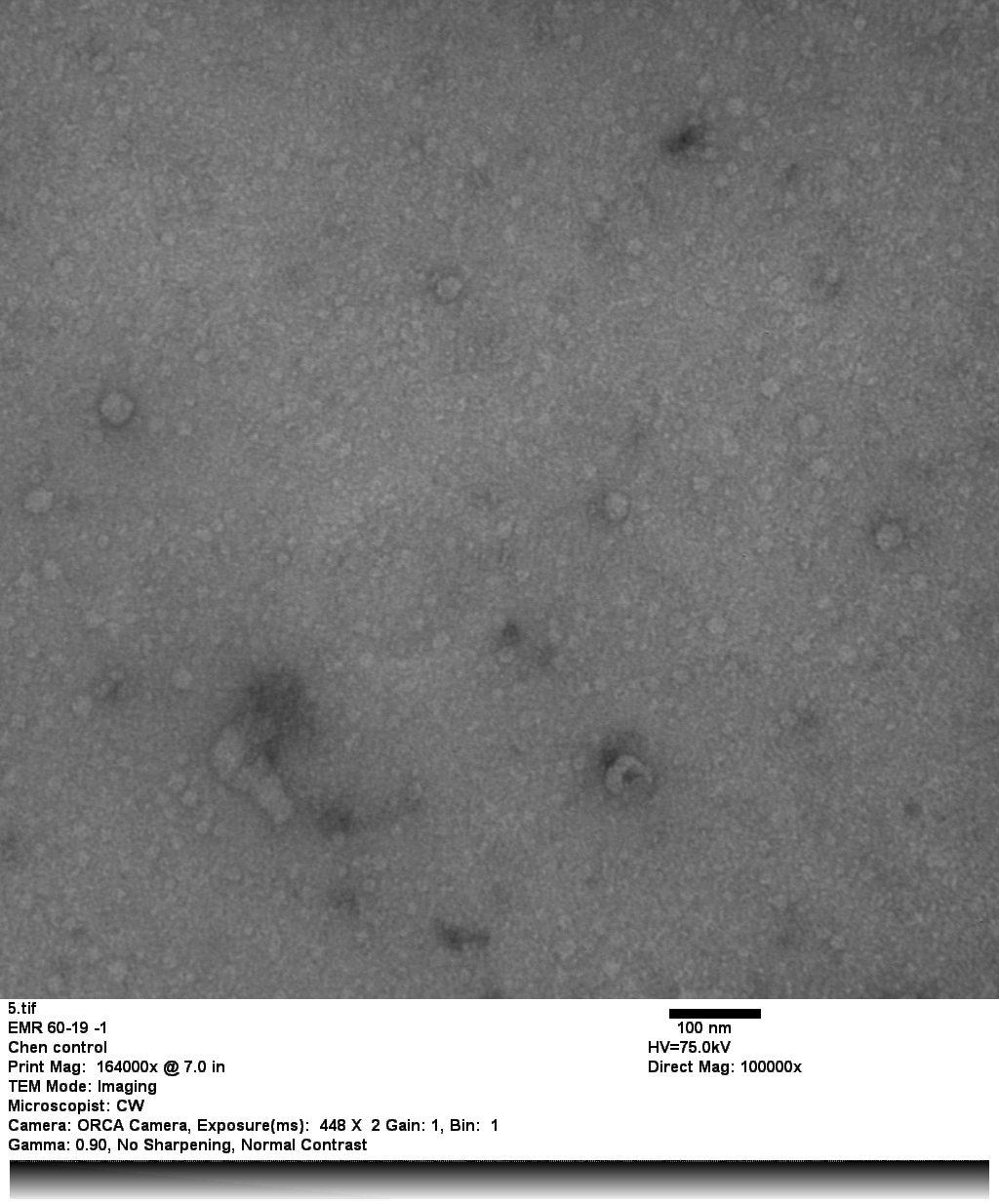

Supplement: Supplementary file 2 [file Datasheet2.zip › Figure 2/20191112_EM-3T3L1-adipo exosomes/control2-100nm.tif]

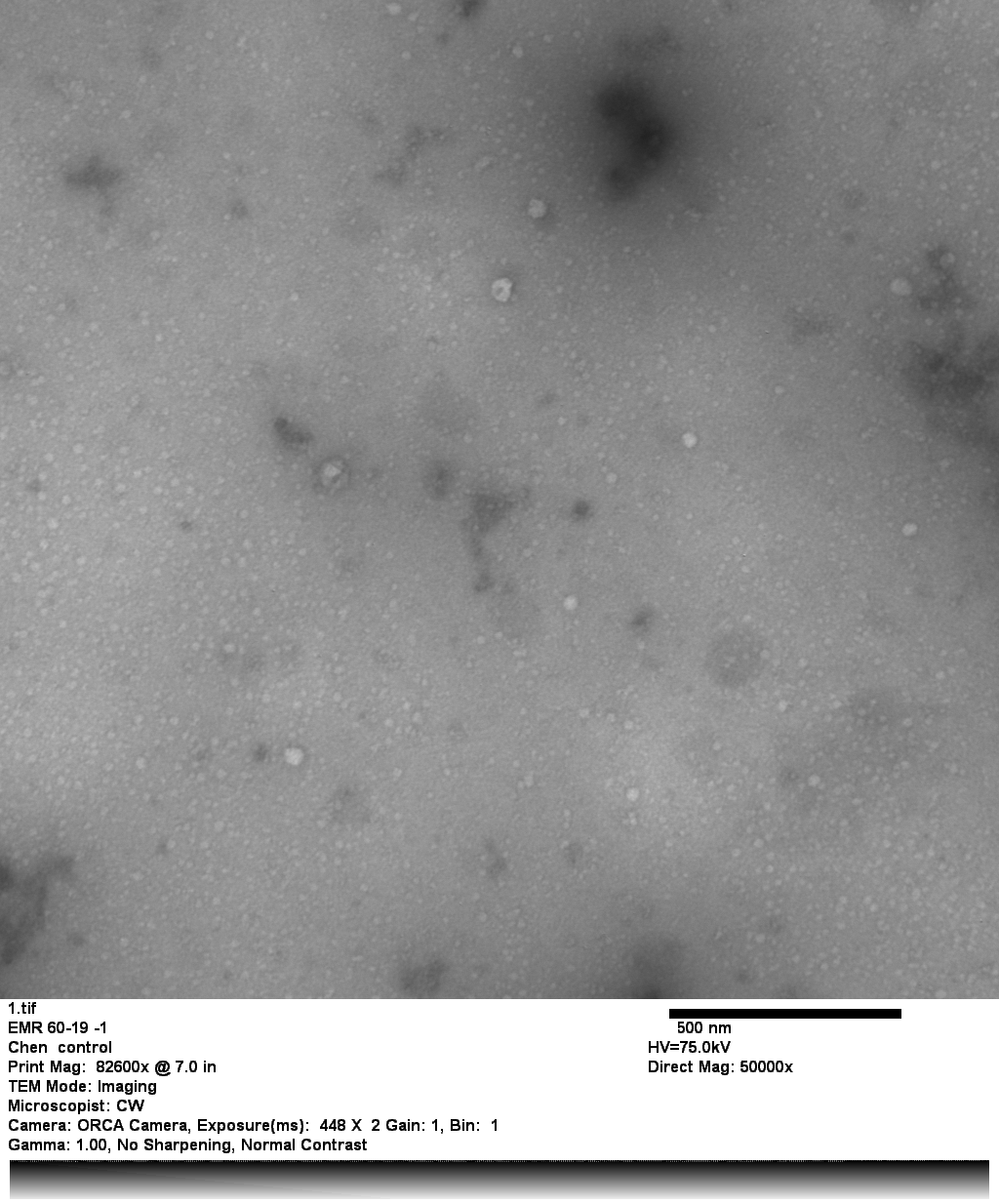

Supplement: Supplementary file 2 [file Datasheet2.zip › Figure 2/20191112_EM-3T3L1-adipo exosomes/control1-500nm.tif]

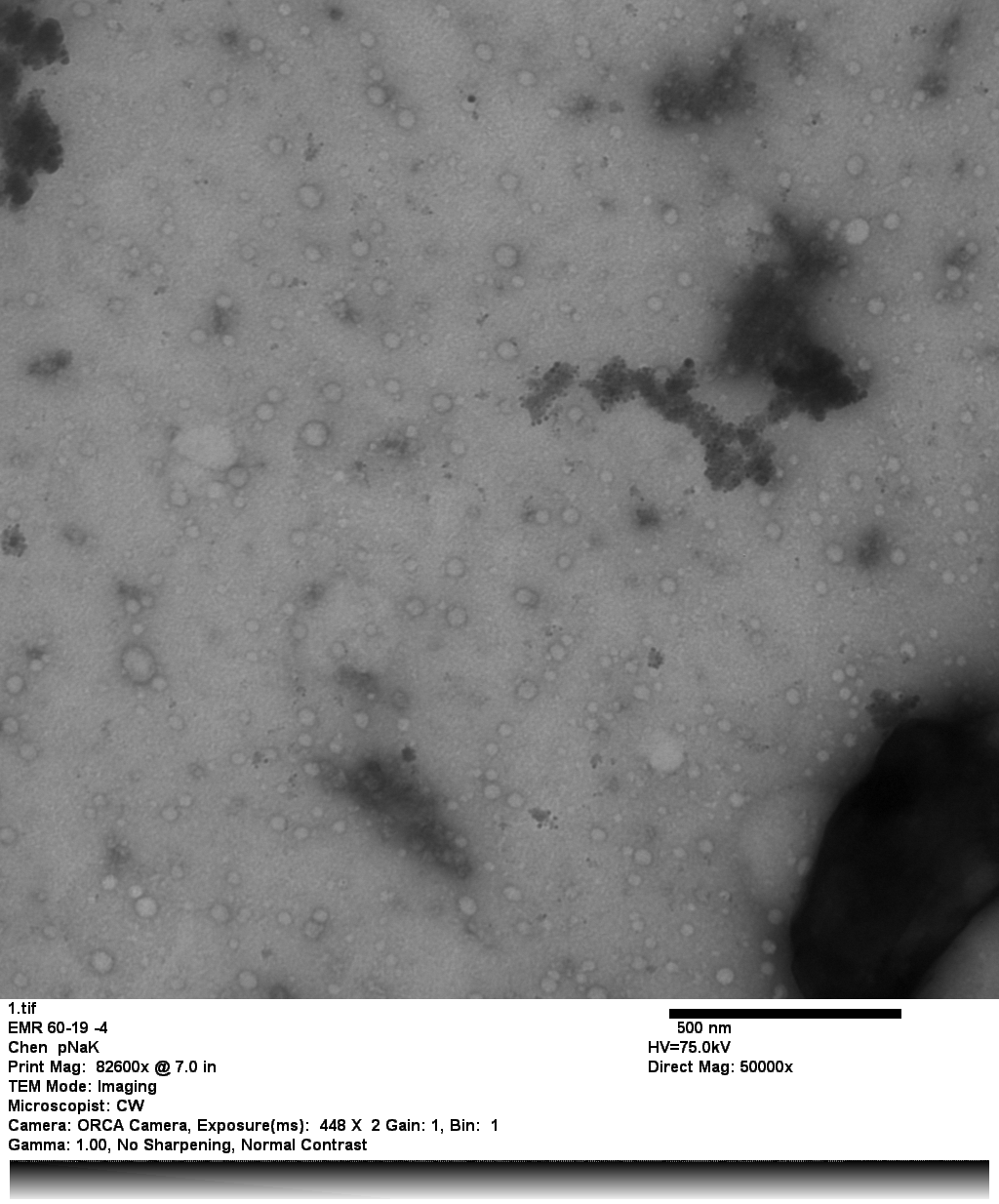

Supplement: Supplementary file 2 [file Datasheet2.zip › Figure 2/20191112_EM-3T3L1-adipo exosomes/pNak-500nm.tif]

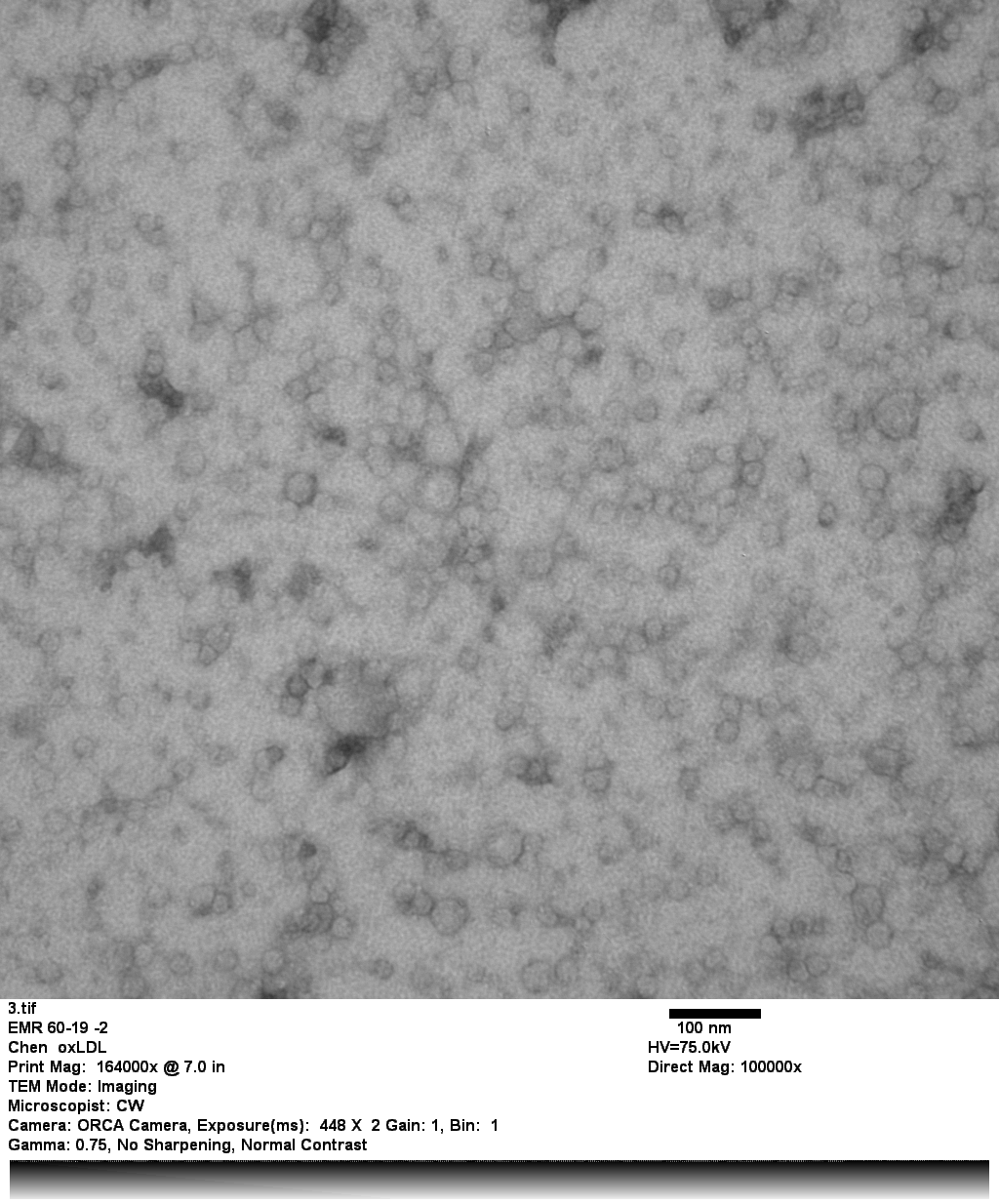

Supplement: Supplementary file 2 [file Datasheet2.zip › Figure 2/20191112_EM-3T3L1-adipo exosomes/oxLDL2-100nm.tif]

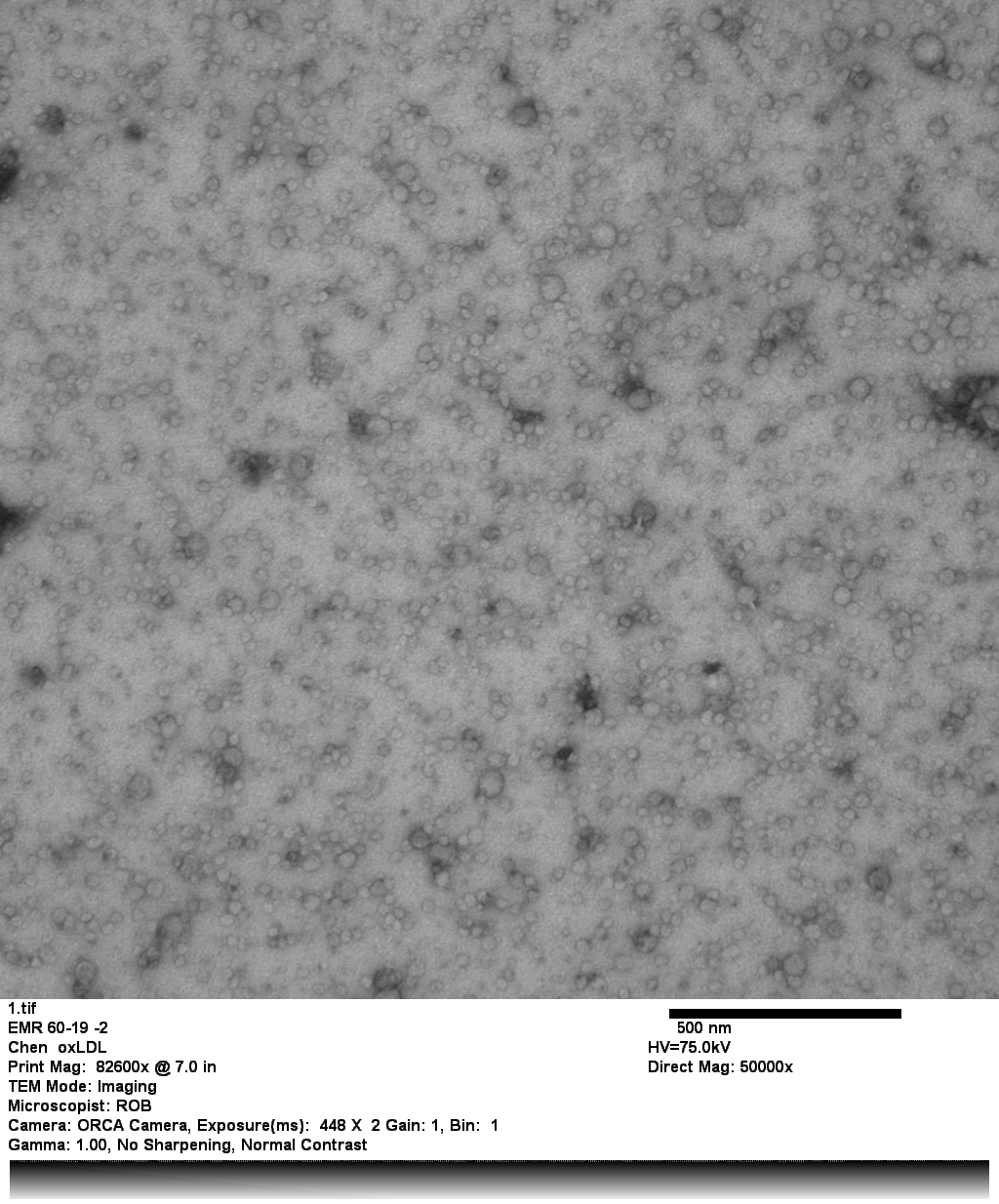

Supplement: Supplementary file 2 [file Datasheet2.zip › Figure 2/20191112_EM-3T3L1-adipo exosomes/oxLDL-500nm.tif]

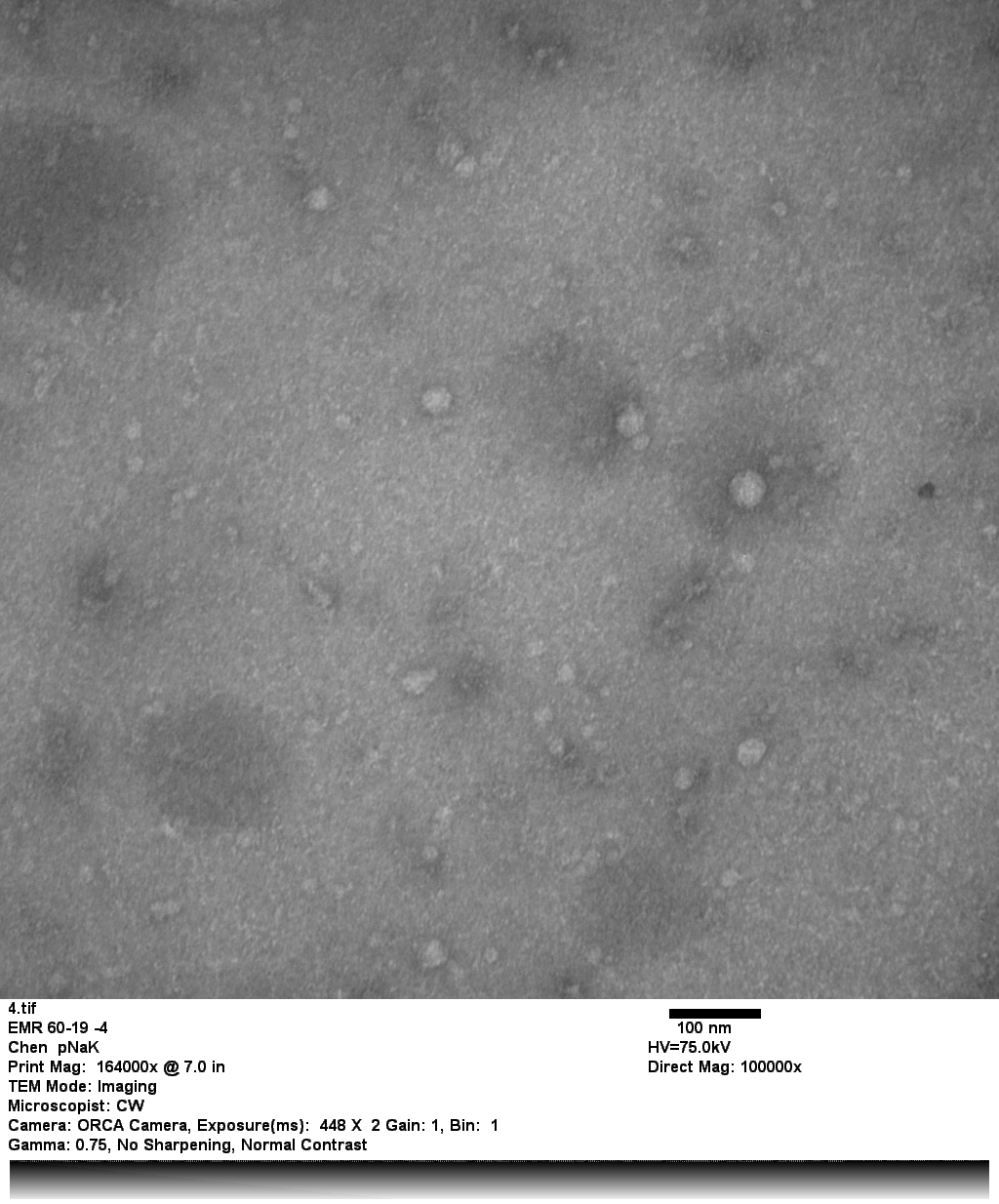

Supplement: Supplementary file 2 [file Datasheet2.zip › Figure 2/20191112_EM-3T3L1-adipo exosomes/pNaK3-100nm.tif]

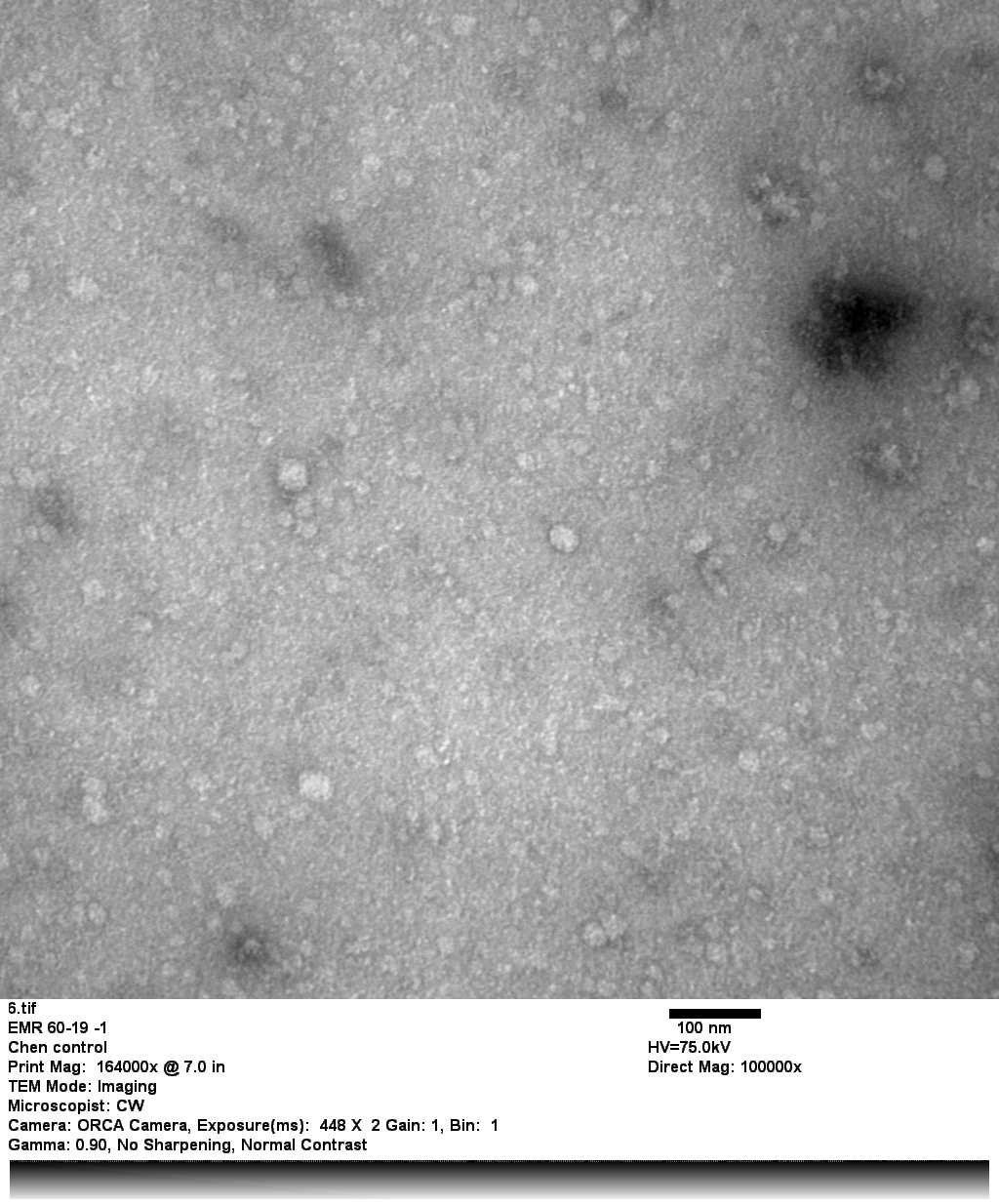

Supplement: Supplementary file 2 [file Datasheet2.zip › Figure 2/20191112_EM-3T3L1-adipo exosomes/control3-100nm.tif]

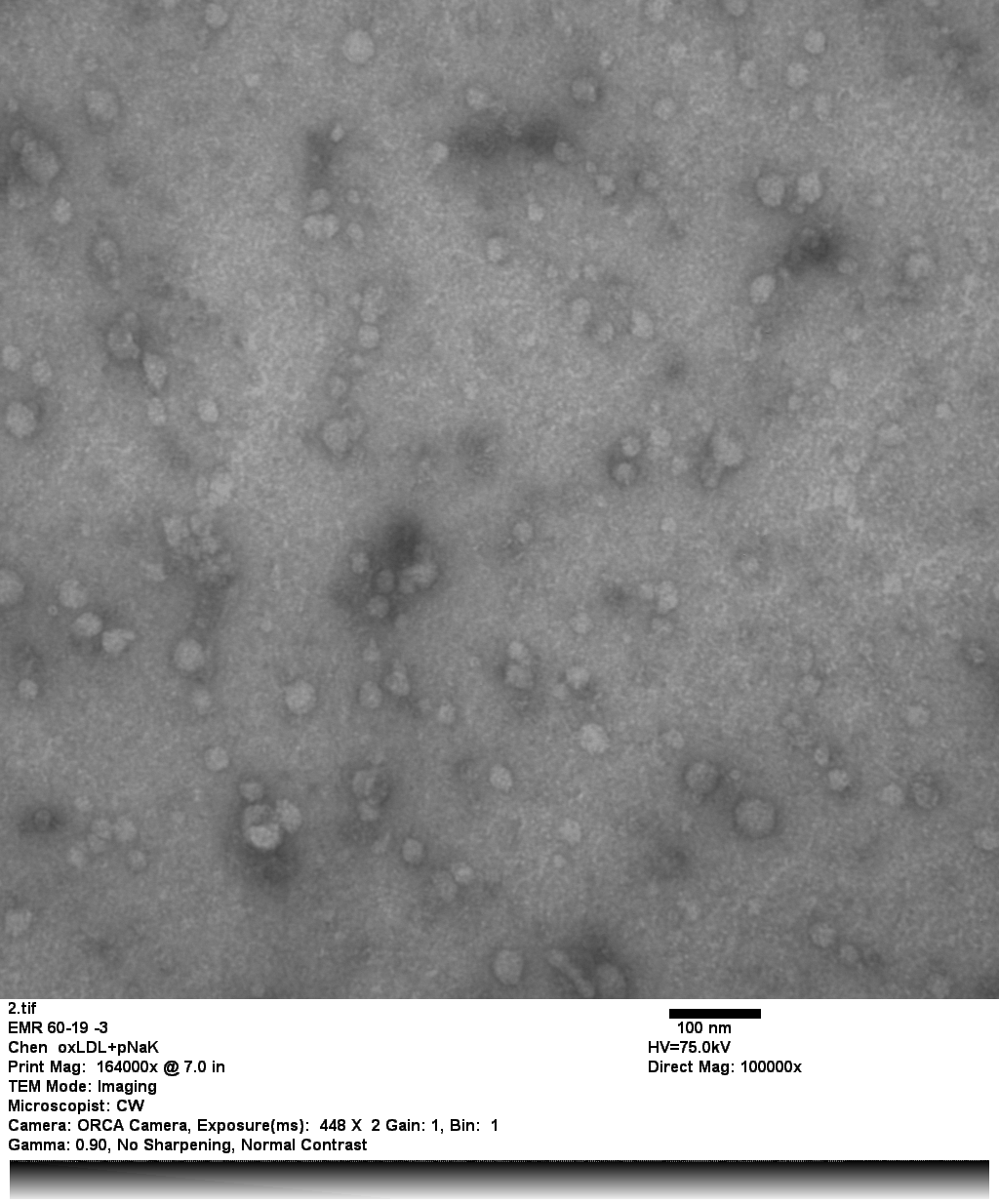

Supplement: Supplementary file 2 [file Datasheet2.zip › Figure 2/20191112_EM-3T3L1-adipo exosomes/oxLDL-pNaK-100nm.tif]

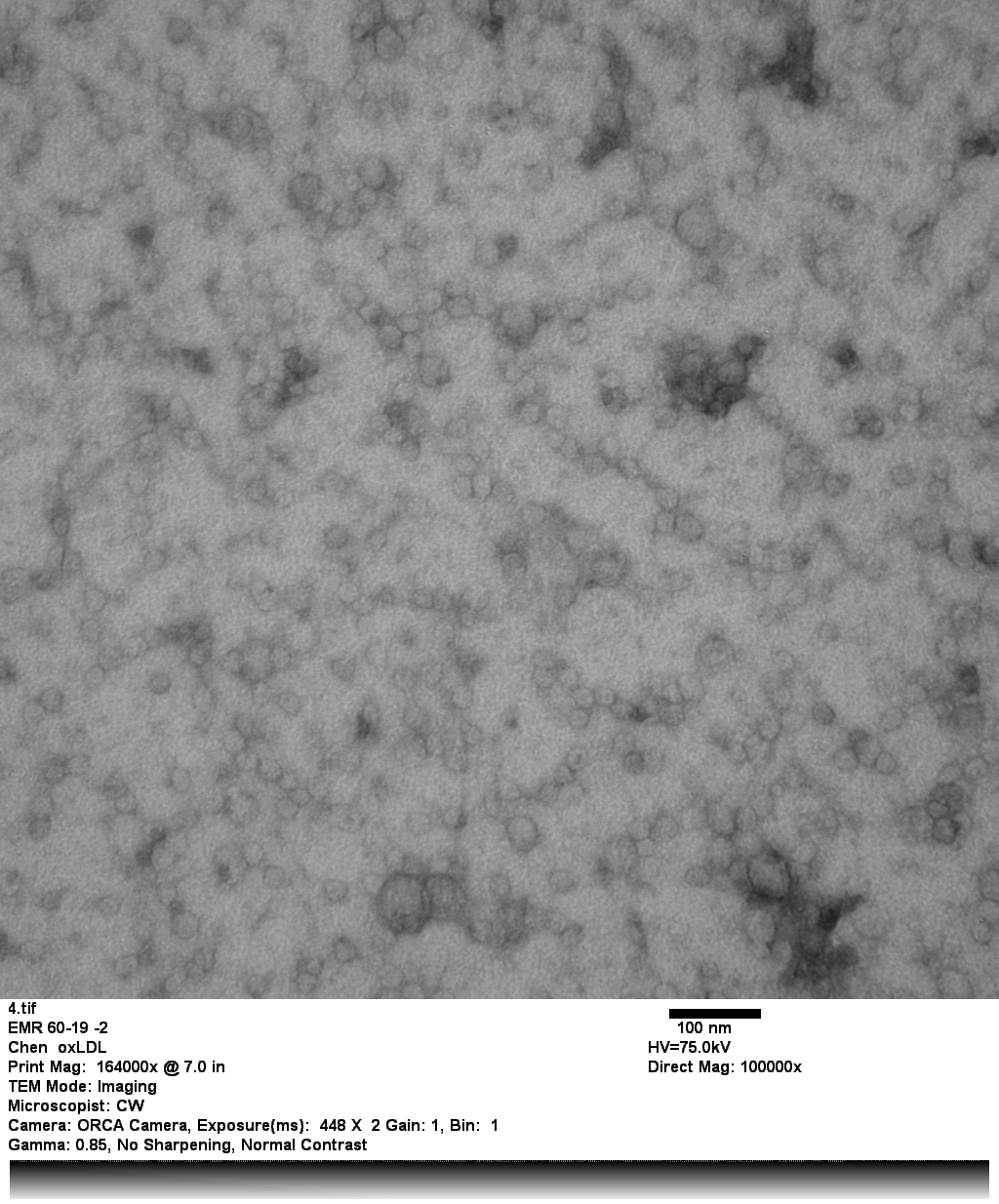

Supplement: Supplementary file 2 [file Datasheet2.zip › Figure 2/20191112_EM-3T3L1-adipo exosomes/oxLDL3-100nm.tif]

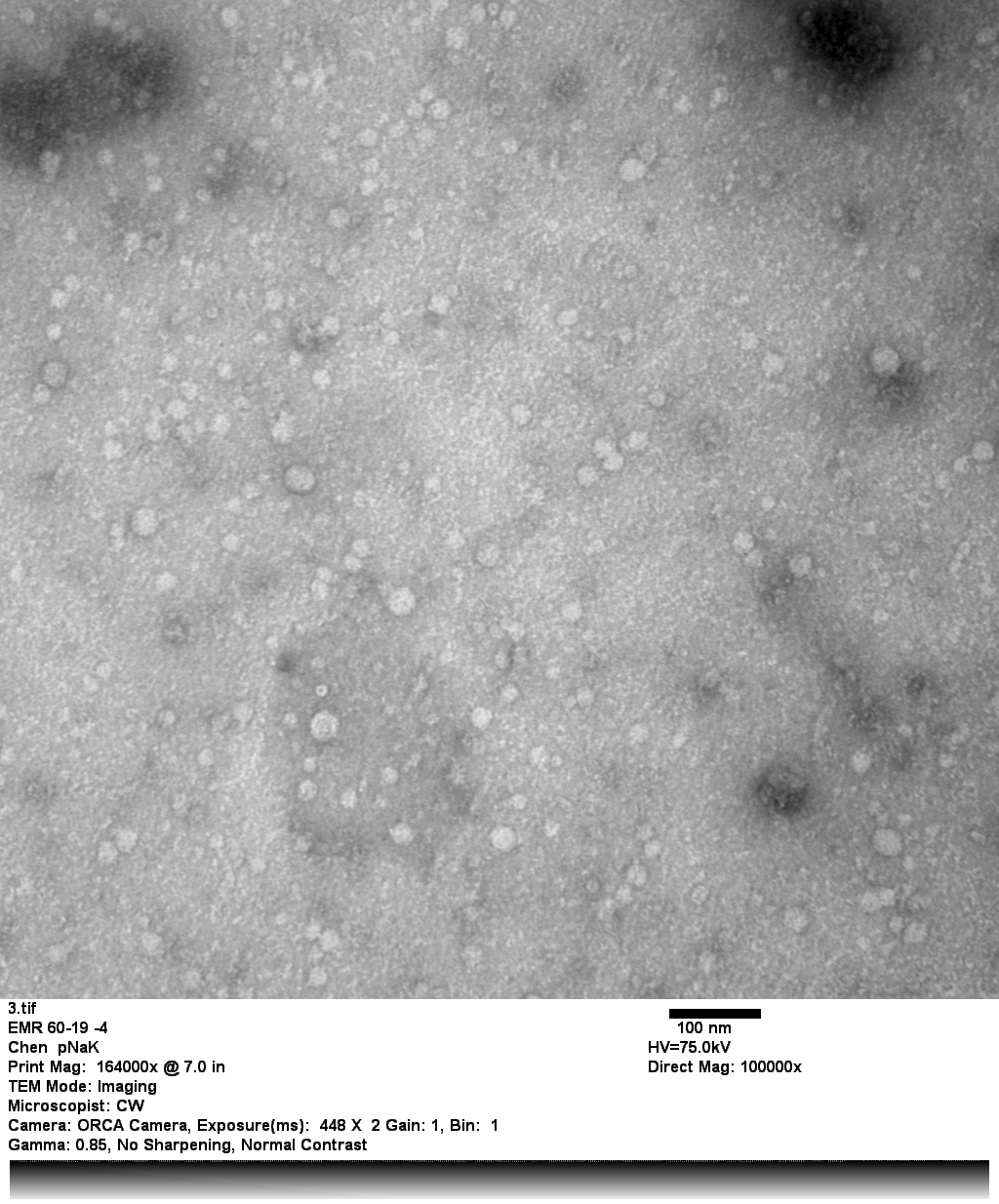

Supplement: Supplementary file 2 [file Datasheet2.zip › Figure 2/20191112_EM-3T3L1-adipo exosomes/pNaK2-100nm.tif]

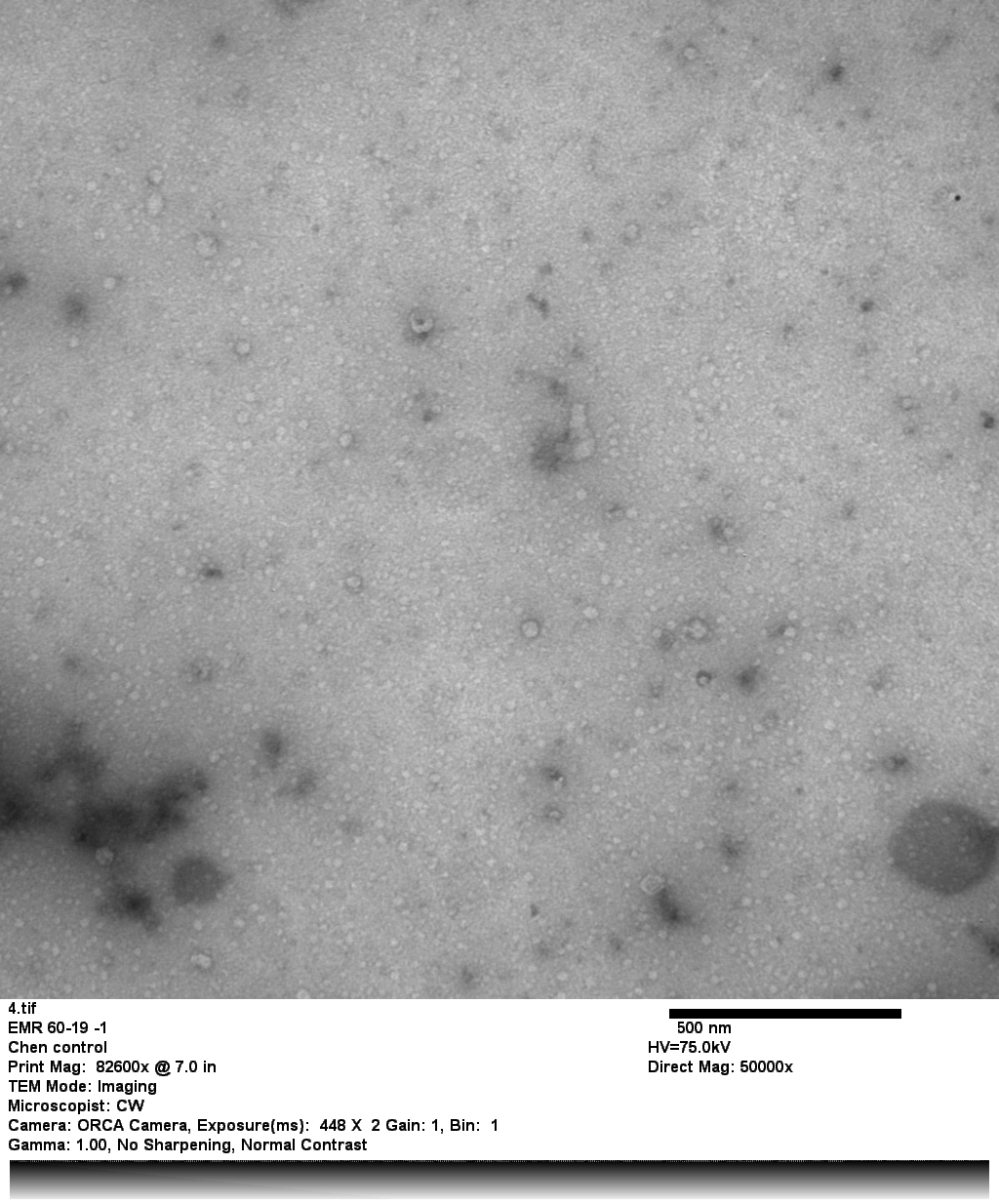

Supplement: Supplementary file 2 [file Datasheet2.zip › Figure 2/20191112_EM-3T3L1-adipo exosomes/control3-500nm.tif]

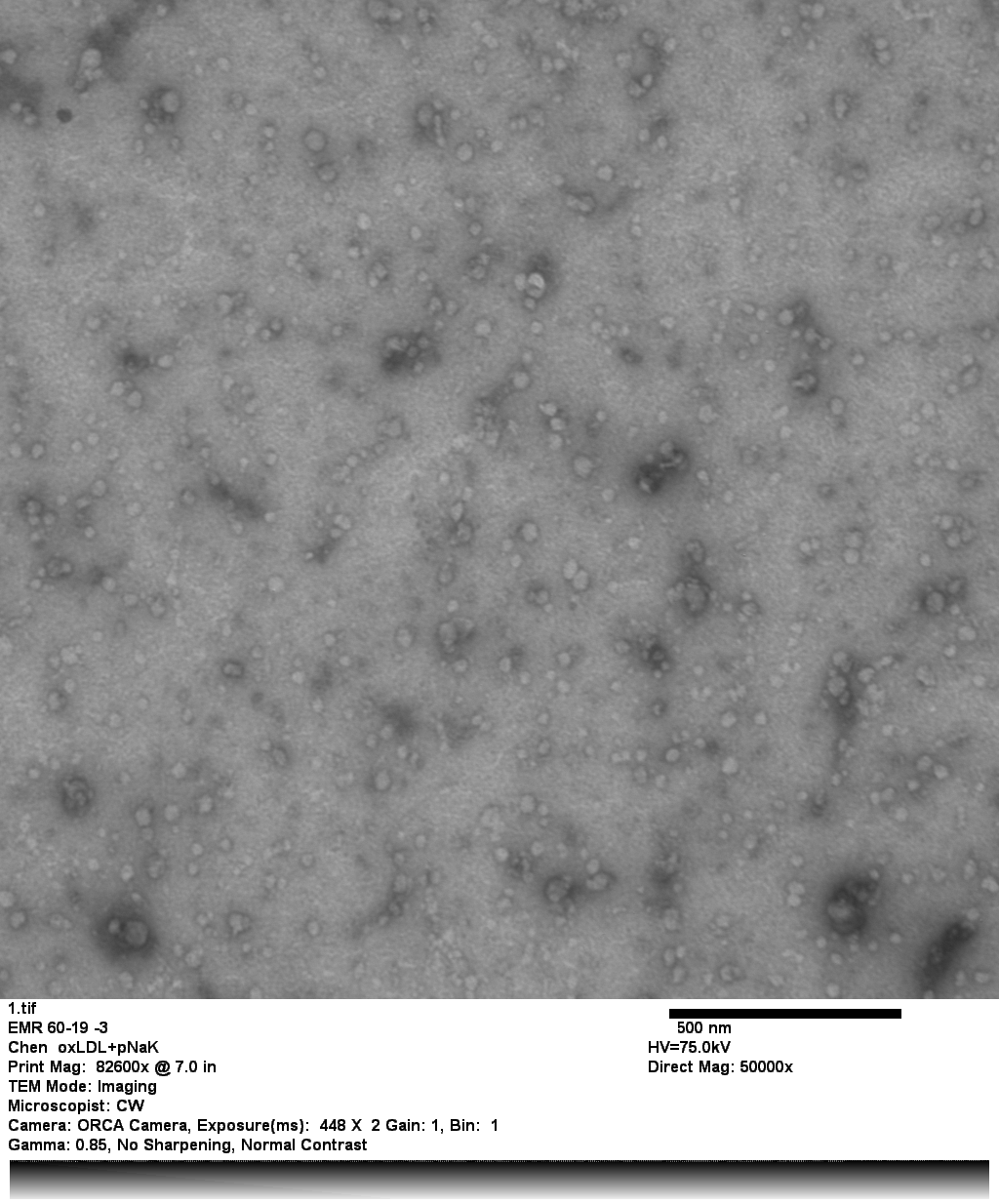

Supplement: Supplementary file 2 [file Datasheet2.zip › Figure 2/20191112_EM-3T3L1-adipo exosomes/oxLDL-pNaK-500nm.tif]

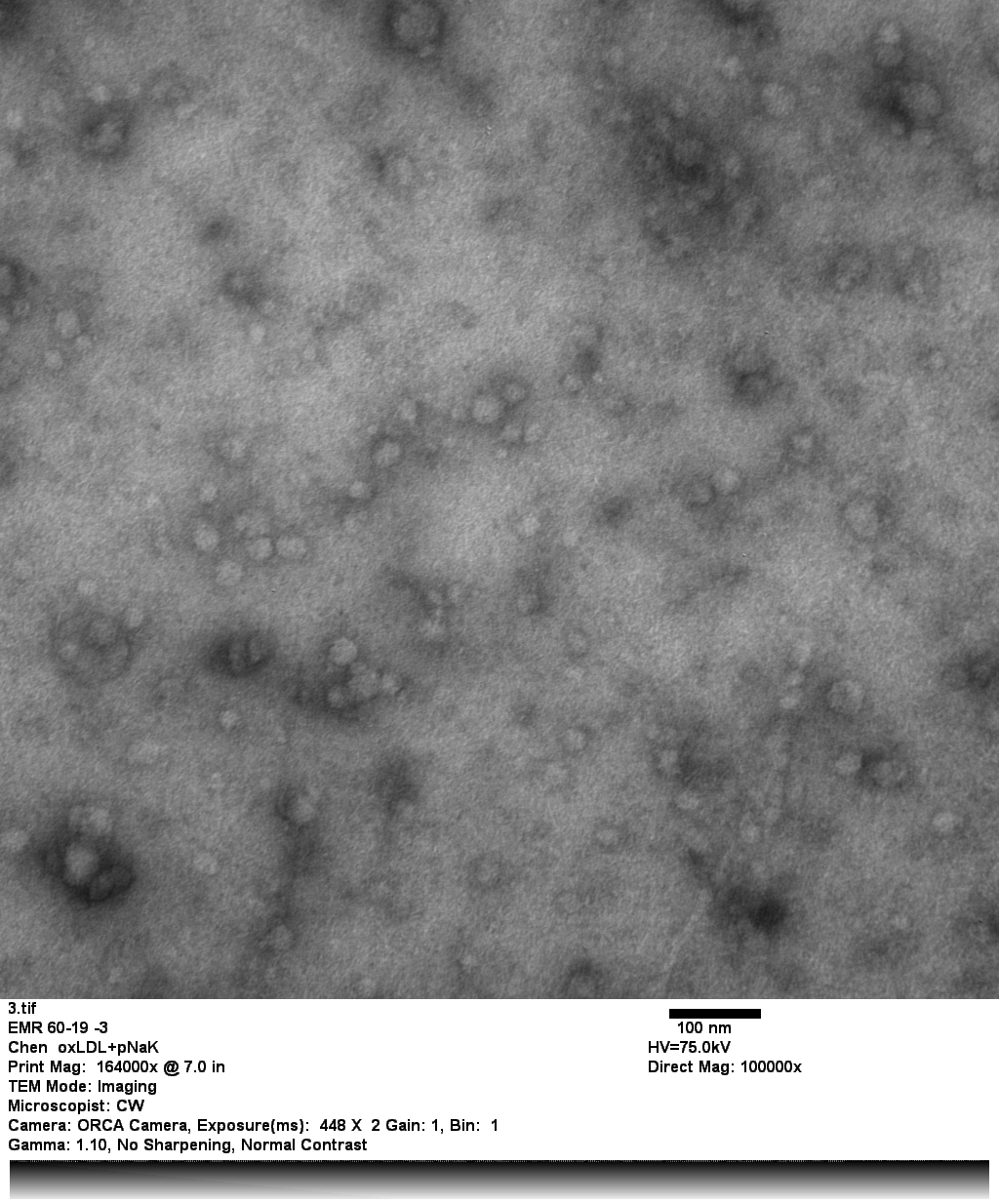

Supplement: Supplementary file 2 [file Datasheet2.zip › Figure 2/20191112_EM-3T3L1-adipo exosomes/oxLDL-pNaK2-100nm.tif]

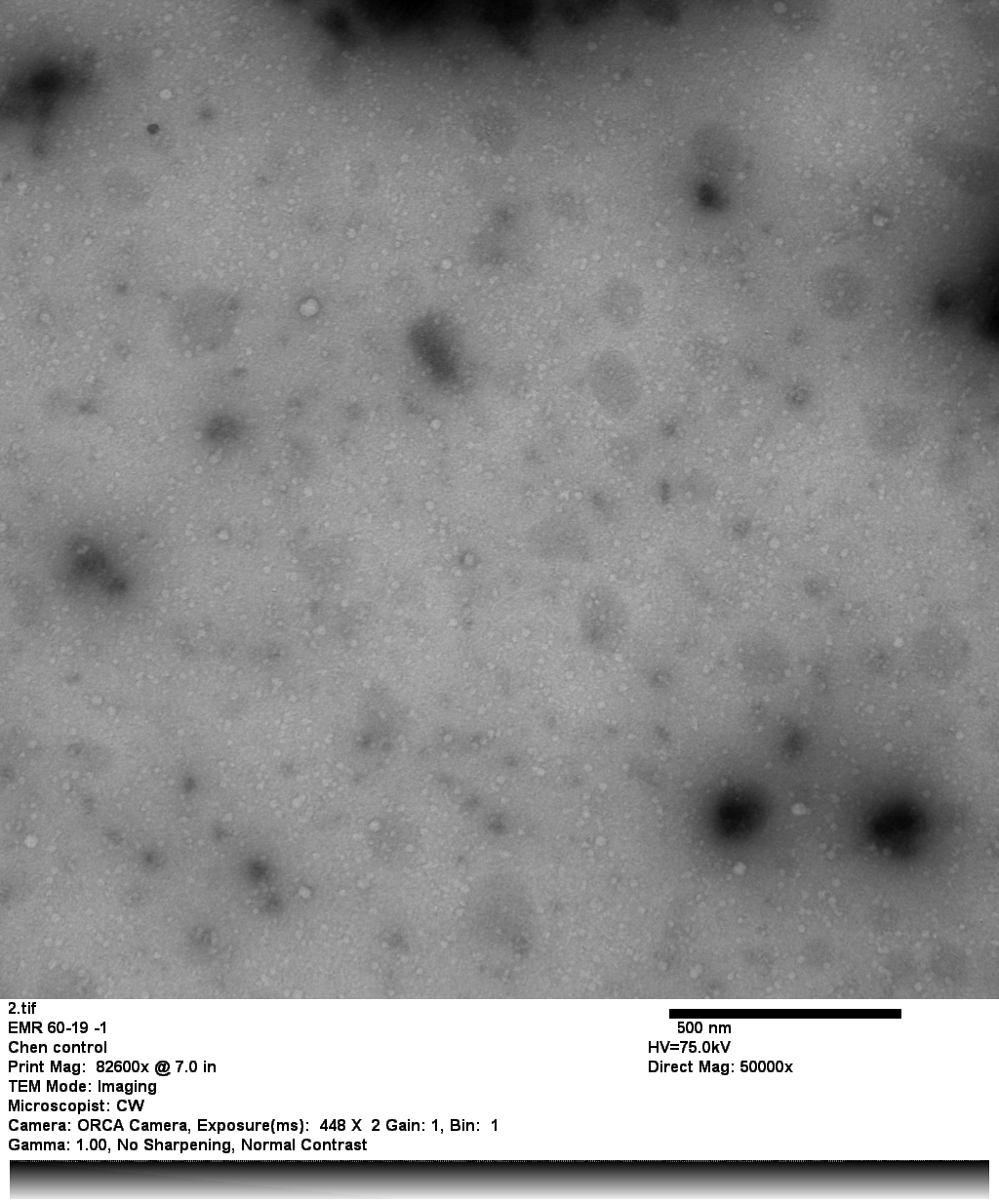

Supplement: Supplementary file 2 [file Datasheet2.zip › Figure 2/20191112_EM-3T3L1-adipo exosomes/control2-500nm.tif]

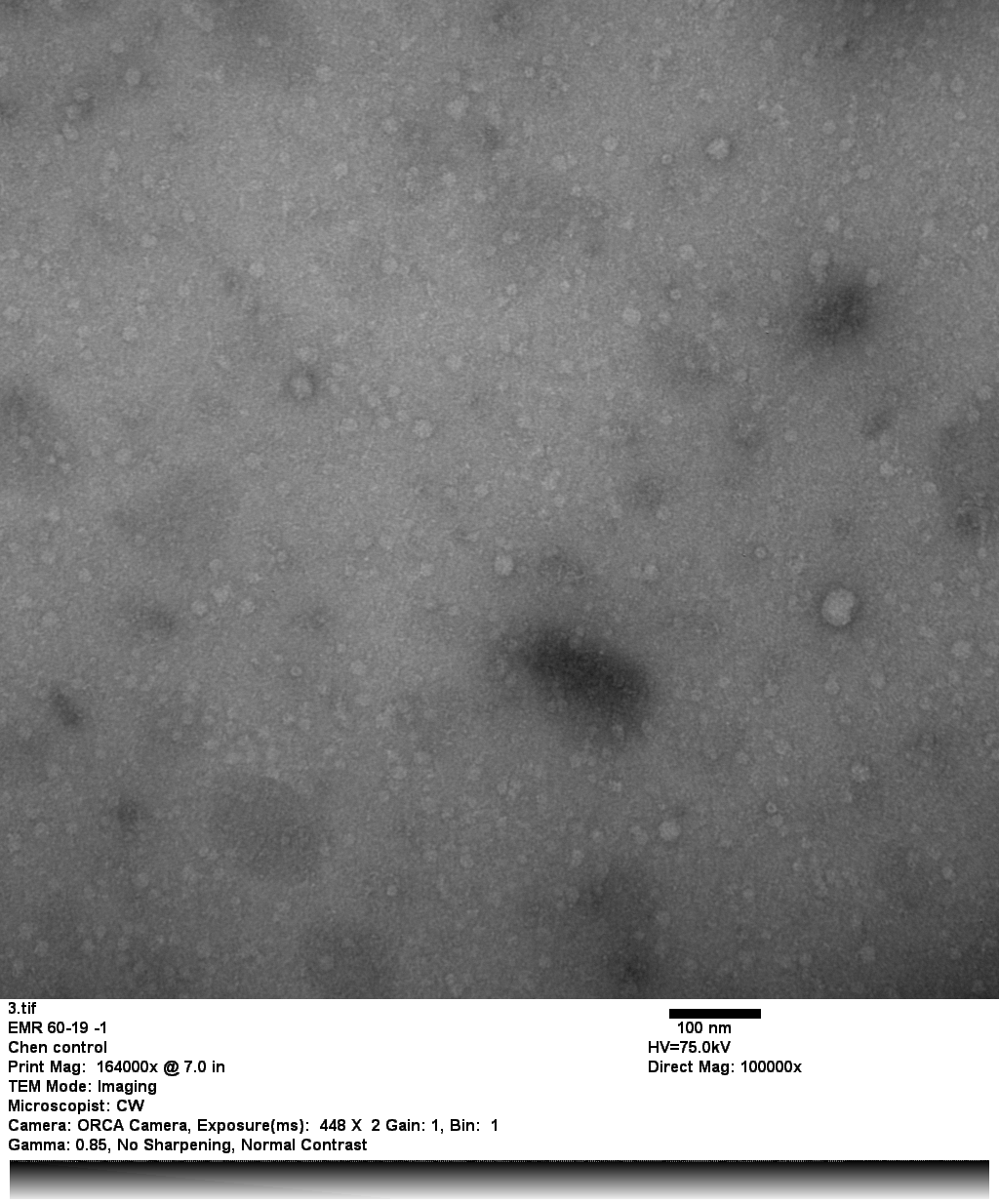

Supplement: Supplementary file 2 [file Datasheet2.zip › Figure 2/20191112_EM-3T3L1-adipo exosomes/control1-100nm.tif]

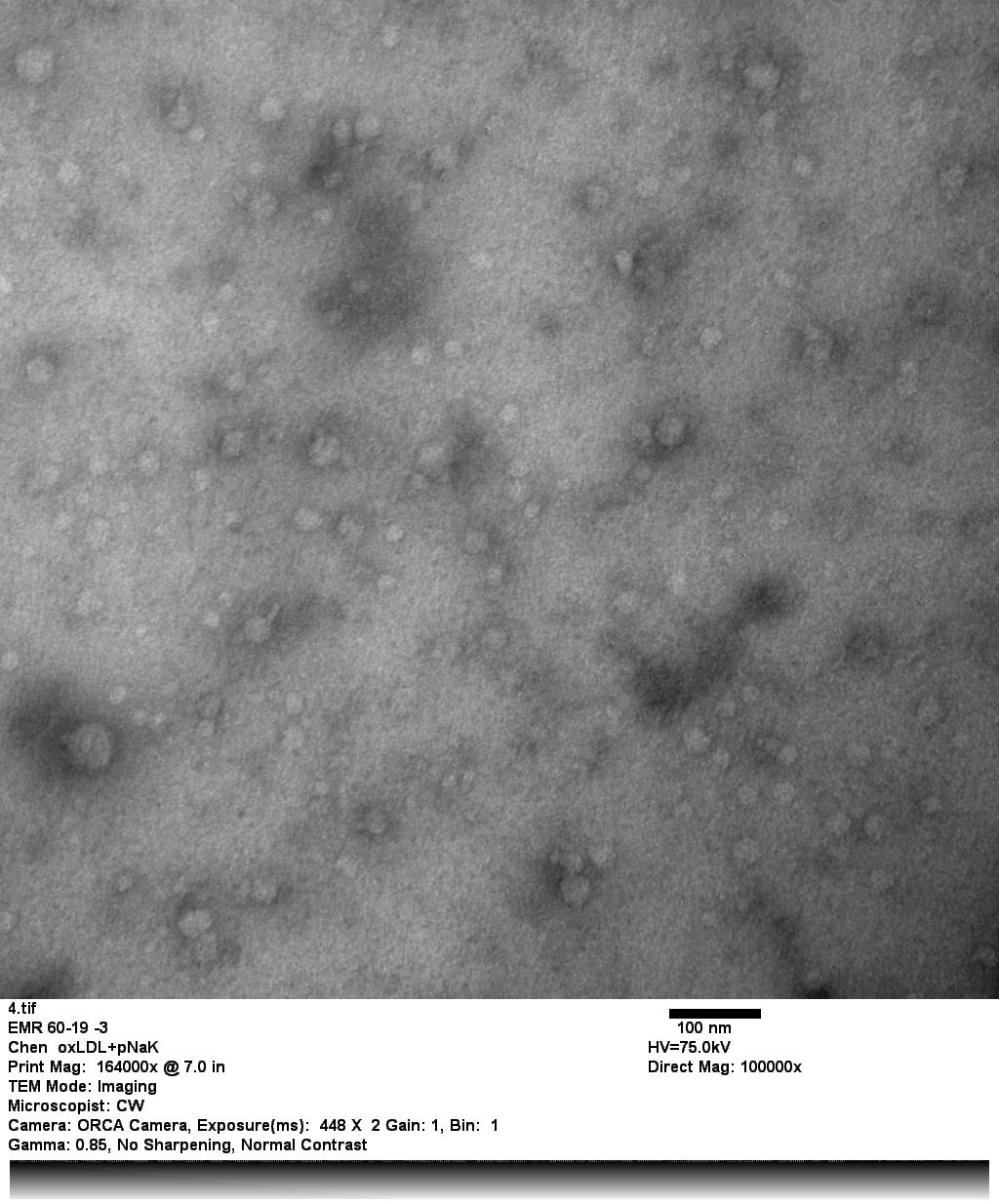

Supplement: Supplementary file 2 [file Datasheet2.zip › Figure 2/20191112_EM-3T3L1-adipo exosomes/oxLDL-pNaK3-100nm.tif]

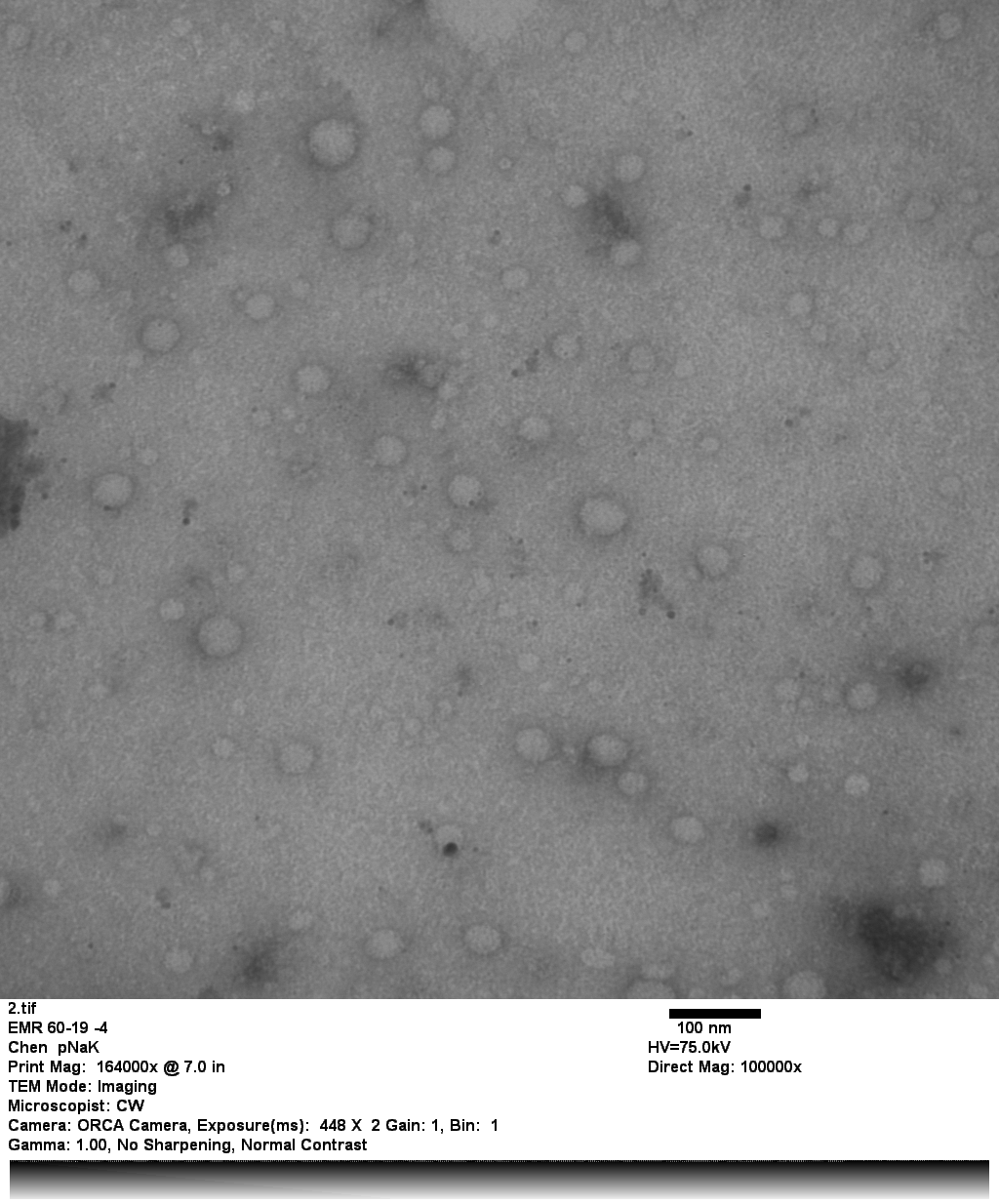

Supplement: Supplementary file 2 [file Datasheet2.zip › Figure 2/20191112_EM-3T3L1-adipo exosomes/pNaK-100nm.tif]

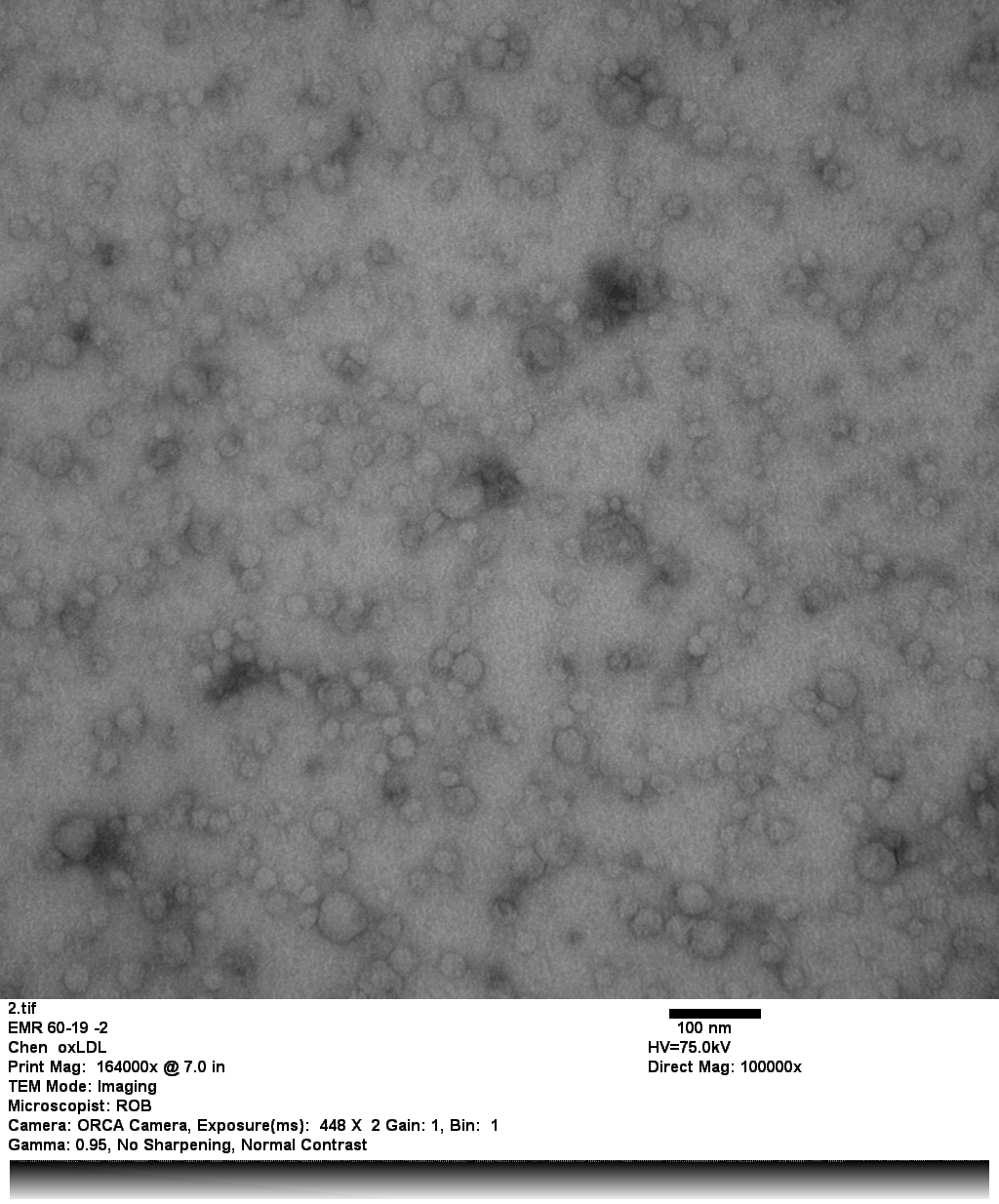

Supplement: Supplementary file 2 [file Datasheet2.zip › Figure 2/20191112_EM-3T3L1-adipo exosomes/oxLDL-100nm.tif]

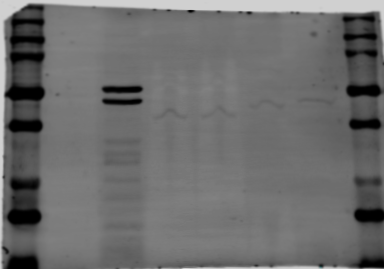

Supplement: Supplementary file 2 [file Datasheet2.zip › Figure 2/3T3L1 Exosome - WB/Tiff images/TSG101/TSG - M7 - 3T3L1 - Set 1.tif]

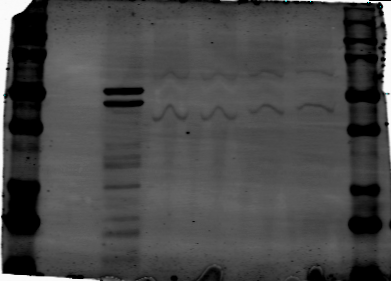

Supplement: Supplementary file 2 [file Datasheet2.zip › Figure 2/3T3L1 Exosome - WB/Tiff images/TSG101/TSG - M9 - 3T3L1 - Set 2 - Rep.tif]

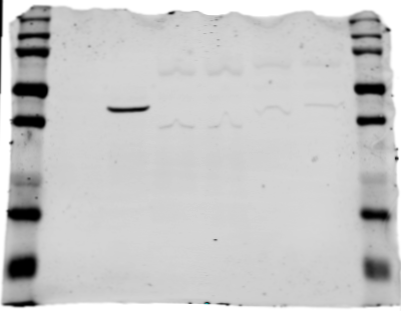

Supplement: Supplementary file 2 [file Datasheet2.zip › Figure 2/3T3L1 Exosome - WB/Tiff images/Actin/Actin - M3 3T3L1 - Set 2 (07-21-22).tif]

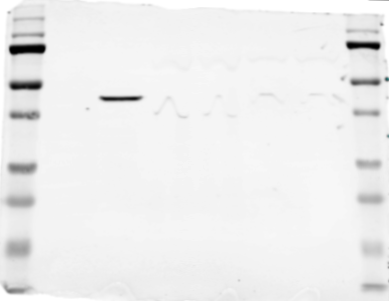

Supplement: Supplementary file 2 [file Datasheet2.zip › Figure 2/3T3L1 Exosome - WB/Tiff images/Actin/Actin - M6 - 3T3L1 - Set 3.tif]

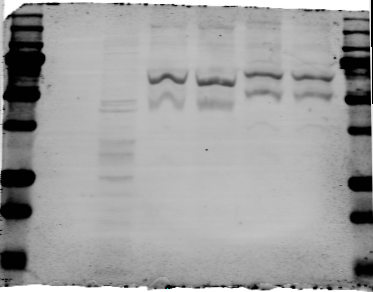

Supplement: Supplementary file 2 [file Datasheet2.zip › Figure 2/3T3L1 Exosome - WB/Tiff images/CD63/CD63 - M5 - 3T3L1 - Set 3 (08-05-22).tif]

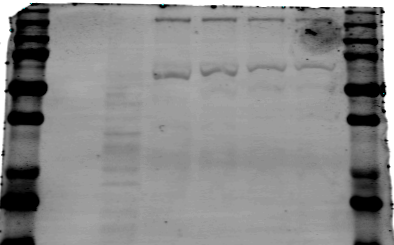

Supplement: Supplementary file 2 [file Datasheet2.zip › Figure 2/3T3L1 Exosome - WB/Tiff images/CD63/CD63 - M1 3T3L1 - Set 1 (07-15-22) - Rep.tif]

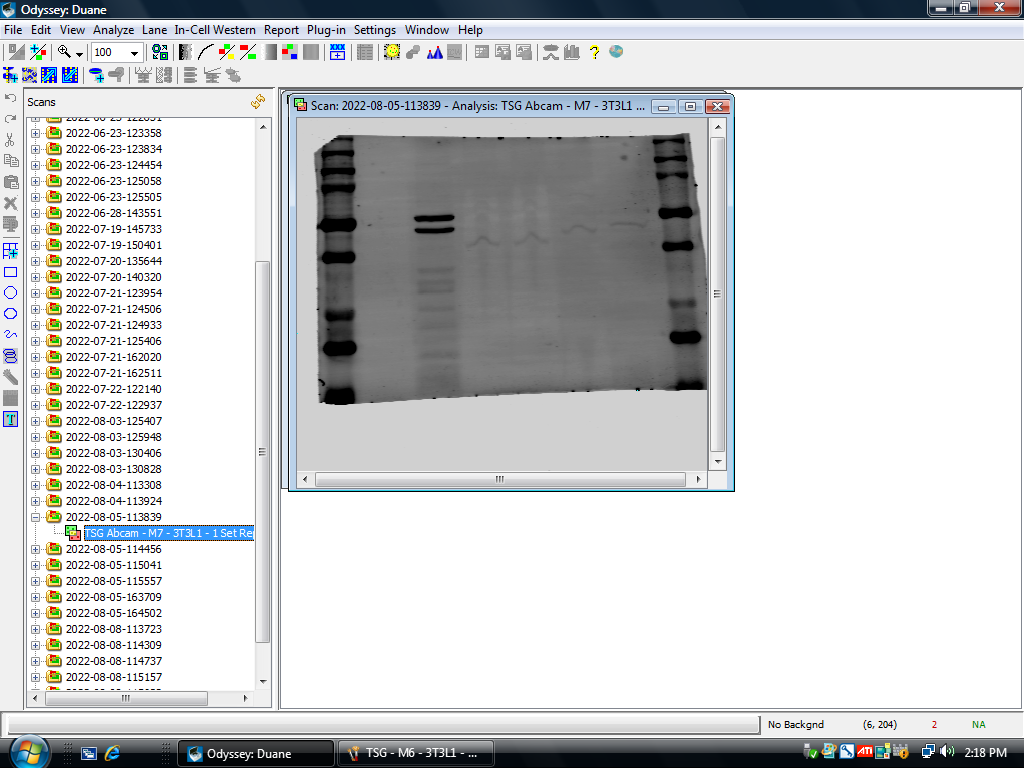

Supplement: Supplementary file 2 [file Datasheet2.zip › Figure 2/3T3L1 Exosome - WB/Raw images/TSG101/TSG - M7 - 3T3L1 - Set 1.tif]

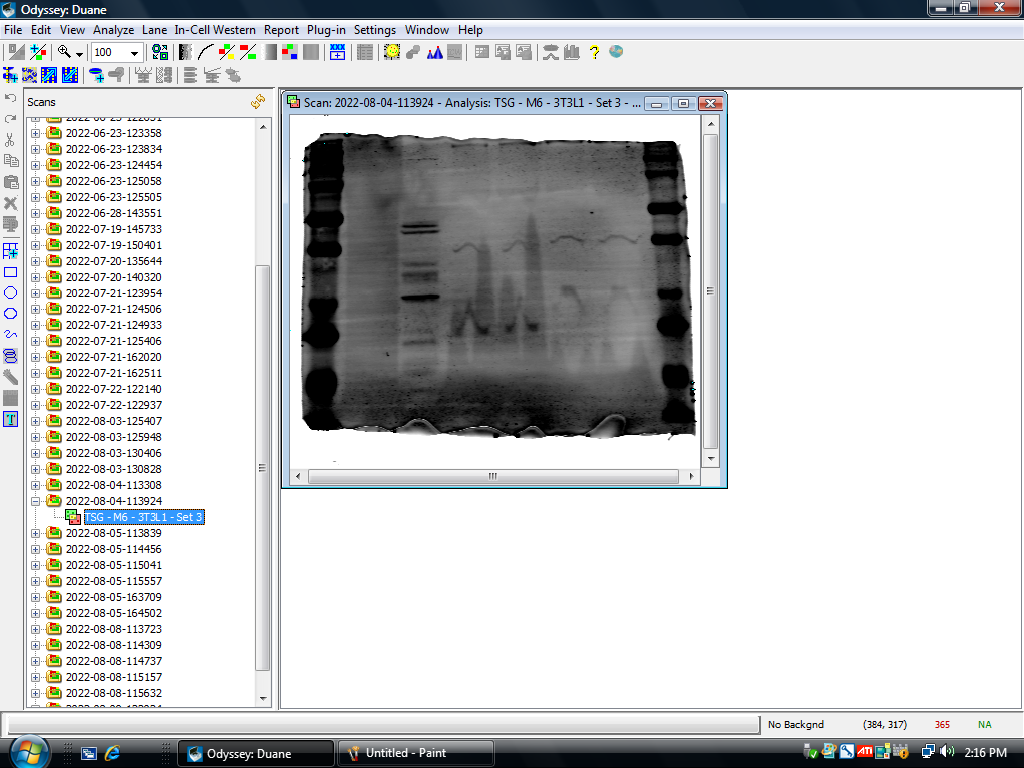

Supplement: Supplementary file 2 [file Datasheet2.zip › Figure 2/3T3L1 Exosome - WB/Raw images/TSG101/TSG - M6 - 3T3L1 - Set 3.tif]

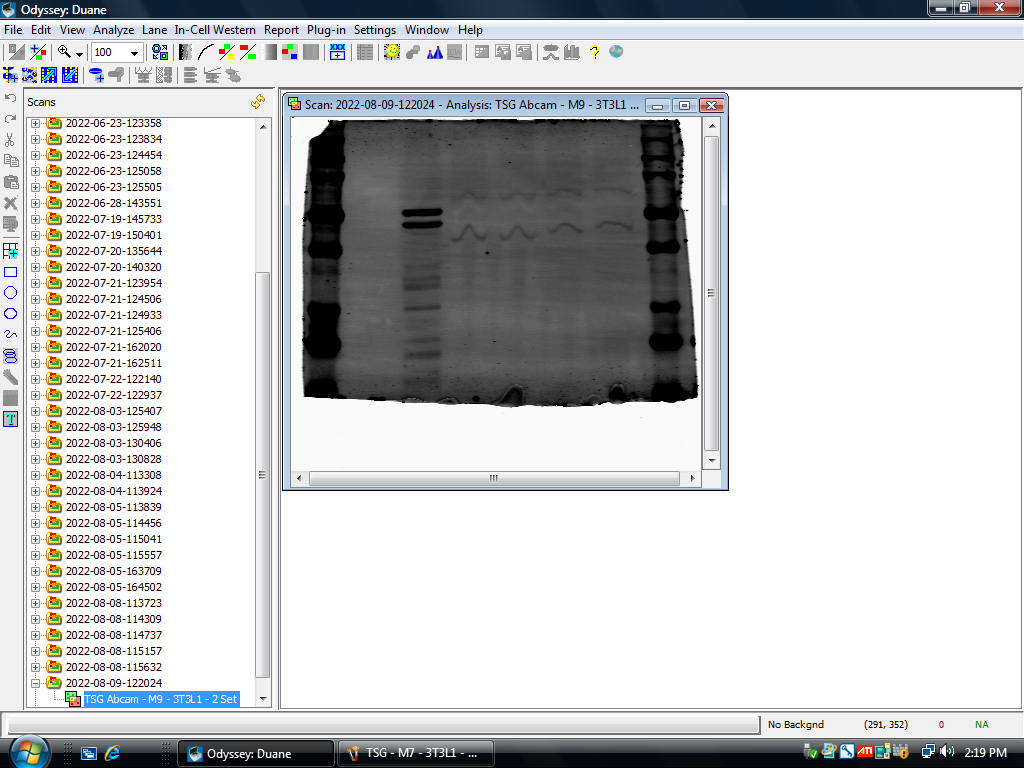

Supplement: Supplementary file 2 [file Datasheet2.zip › Figure 2/3T3L1 Exosome - WB/Raw images/TSG101/TSG - M9 - 3T3L1 - Set 2 - Rep.tif]

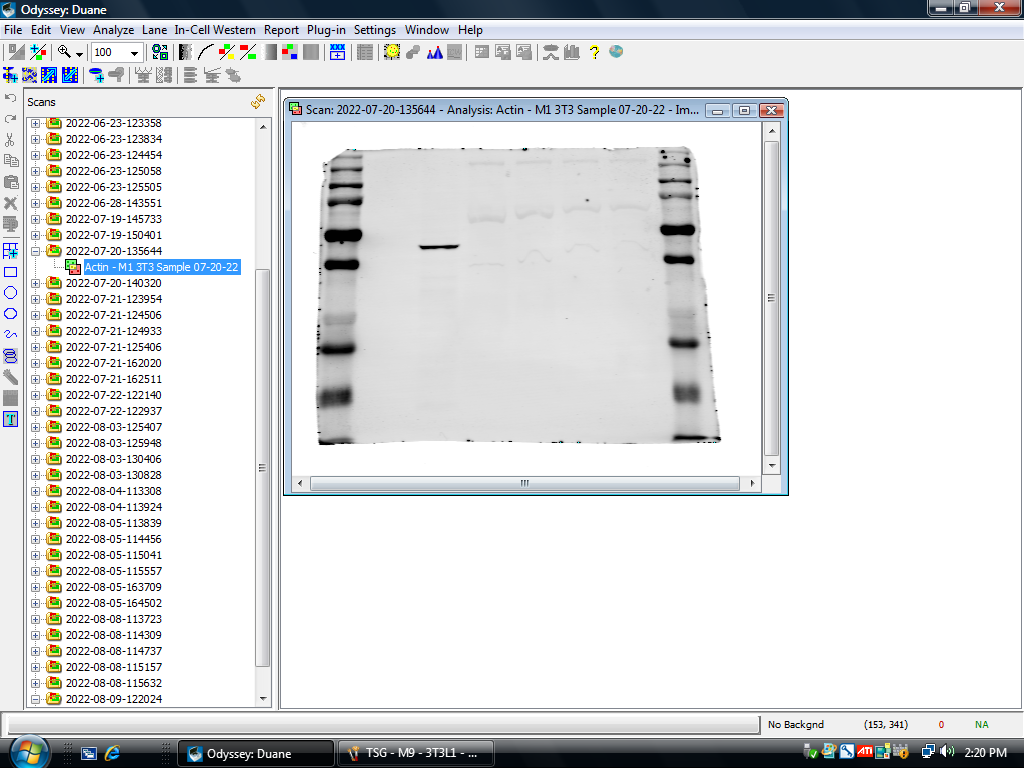

Supplement: Supplementary file 2 [file Datasheet2.zip › Figure 2/3T3L1 Exosome - WB/Raw images/Actin/Actin - M1 - 3T3L1 - Set 1 - Rep.tif]

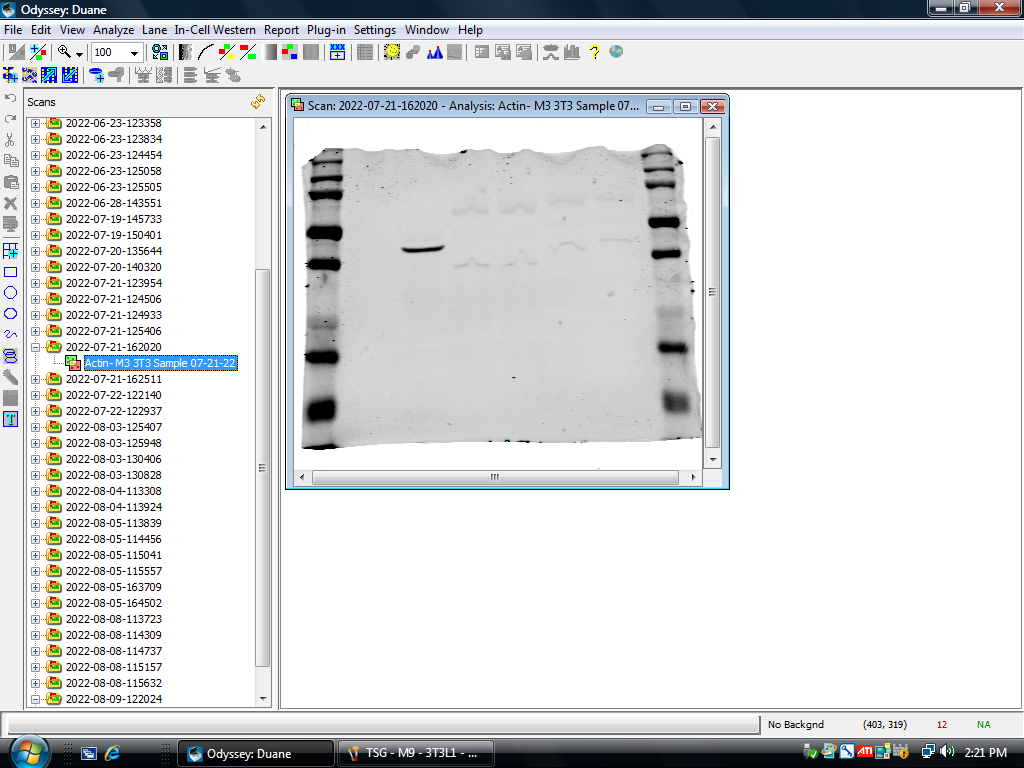

Supplement: Supplementary file 2 [file Datasheet2.zip › Figure 2/3T3L1 Exosome - WB/Raw images/Actin/Actin - M3 - 3T3L1 - Set 2.tif]

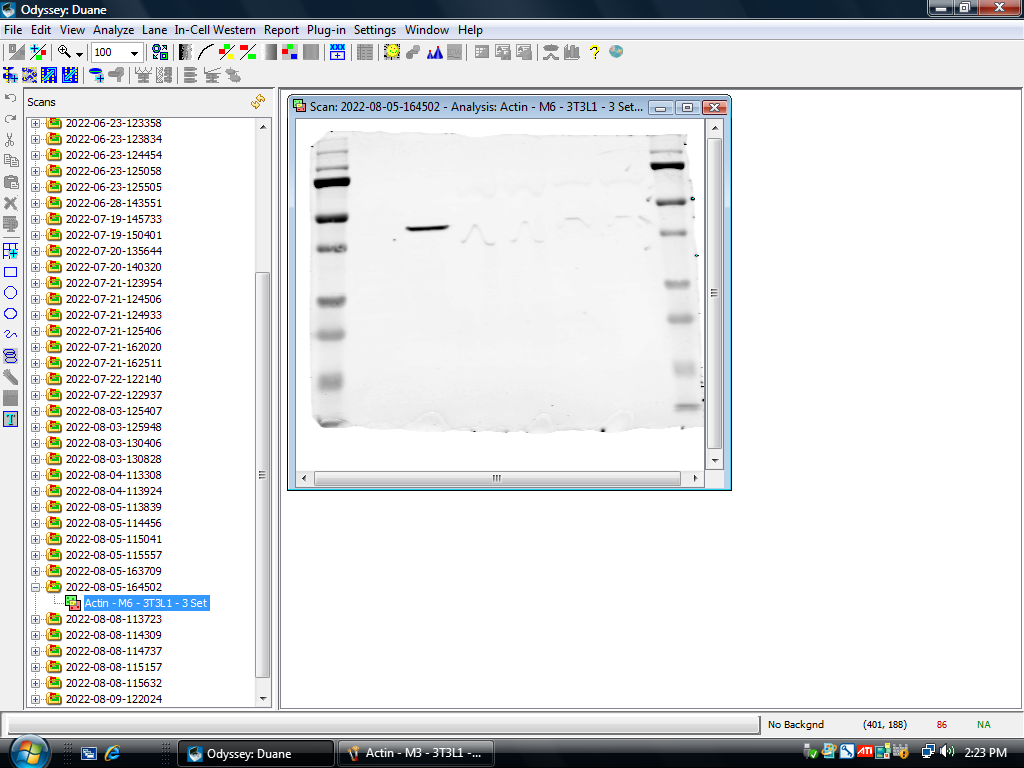

Supplement: Supplementary file 2 [file Datasheet2.zip › Figure 2/3T3L1 Exosome - WB/Raw images/Actin/Actin - M6 - 3T3L1 - Set 3.tif]

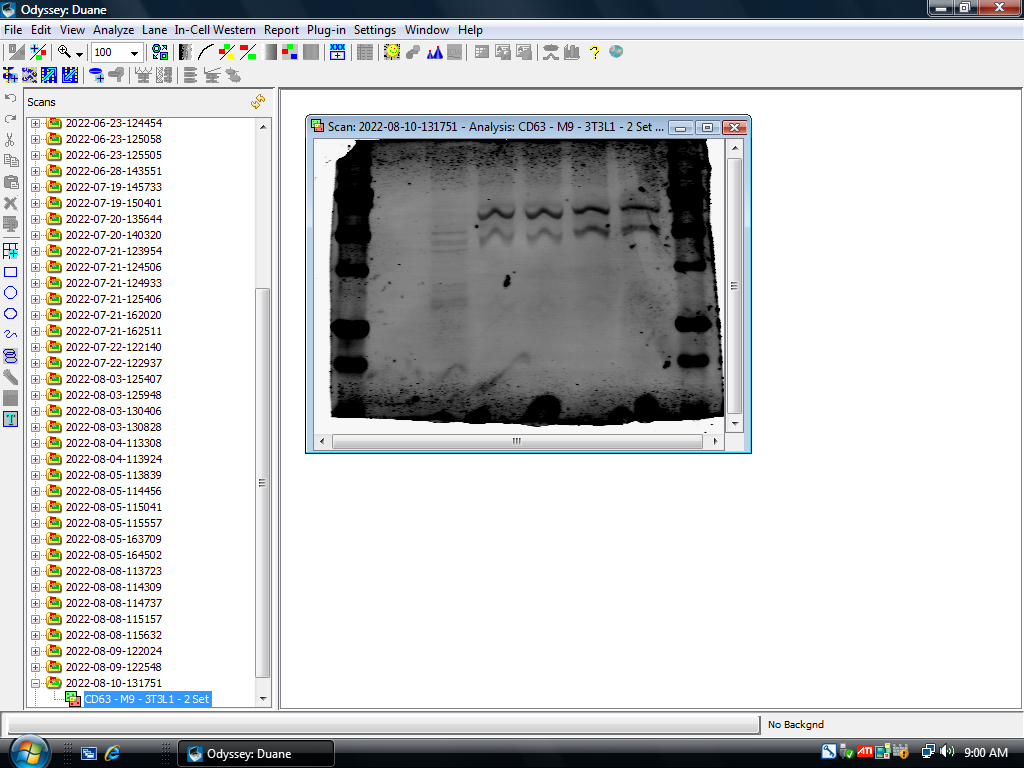

Supplement: Supplementary file 2 [file Datasheet2.zip › Figure 2/3T3L1 Exosome - WB/Raw images/CD63/CD63 - M9 - 3T3L1 - Set 2.tif]

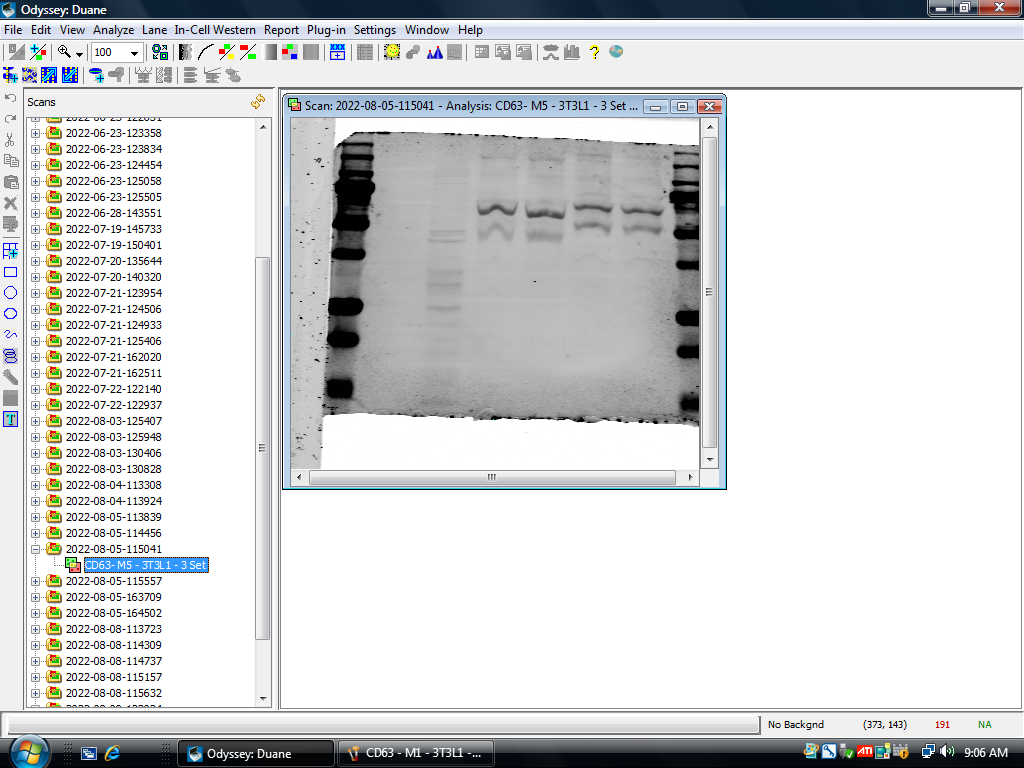

Supplement: Supplementary file 2 [file Datasheet2.zip › Figure 2/3T3L1 Exosome - WB/Raw images/CD63/CD63 - M5 - 3T3L1 - Set 3.tif]

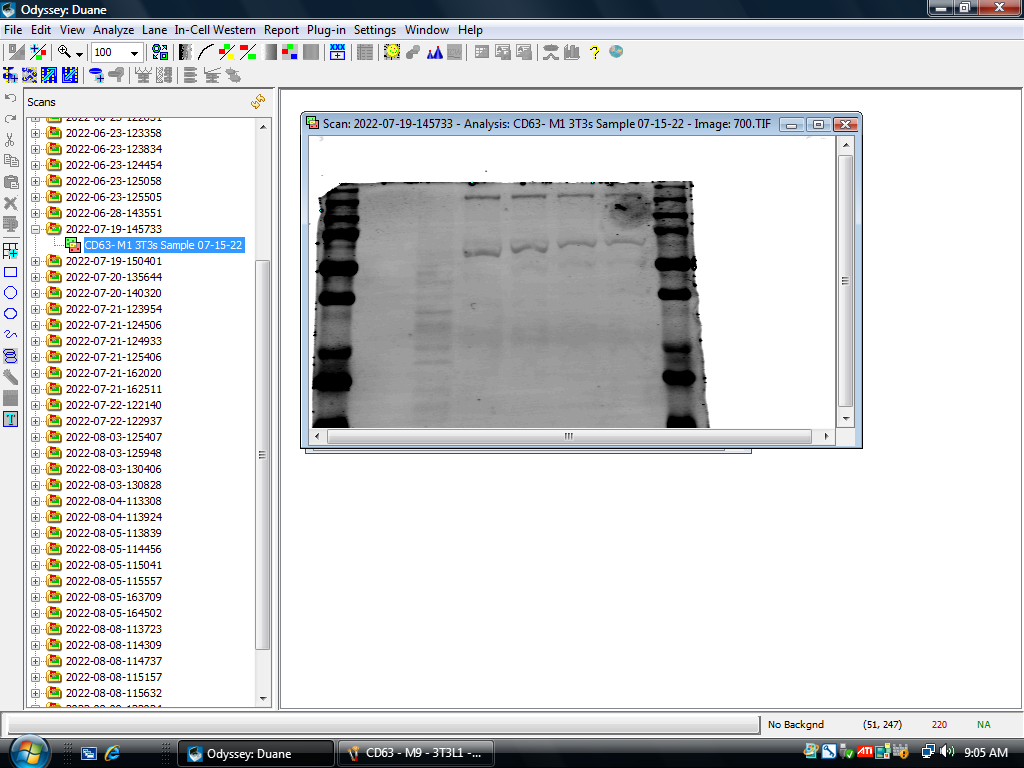

Supplement: Supplementary file 2 [file Datasheet2.zip › Figure 2/3T3L1 Exosome - WB/Raw images/CD63/CD63 - M1 - 3T3L1 - Set 1 - Rep.tif]

## Slide 1
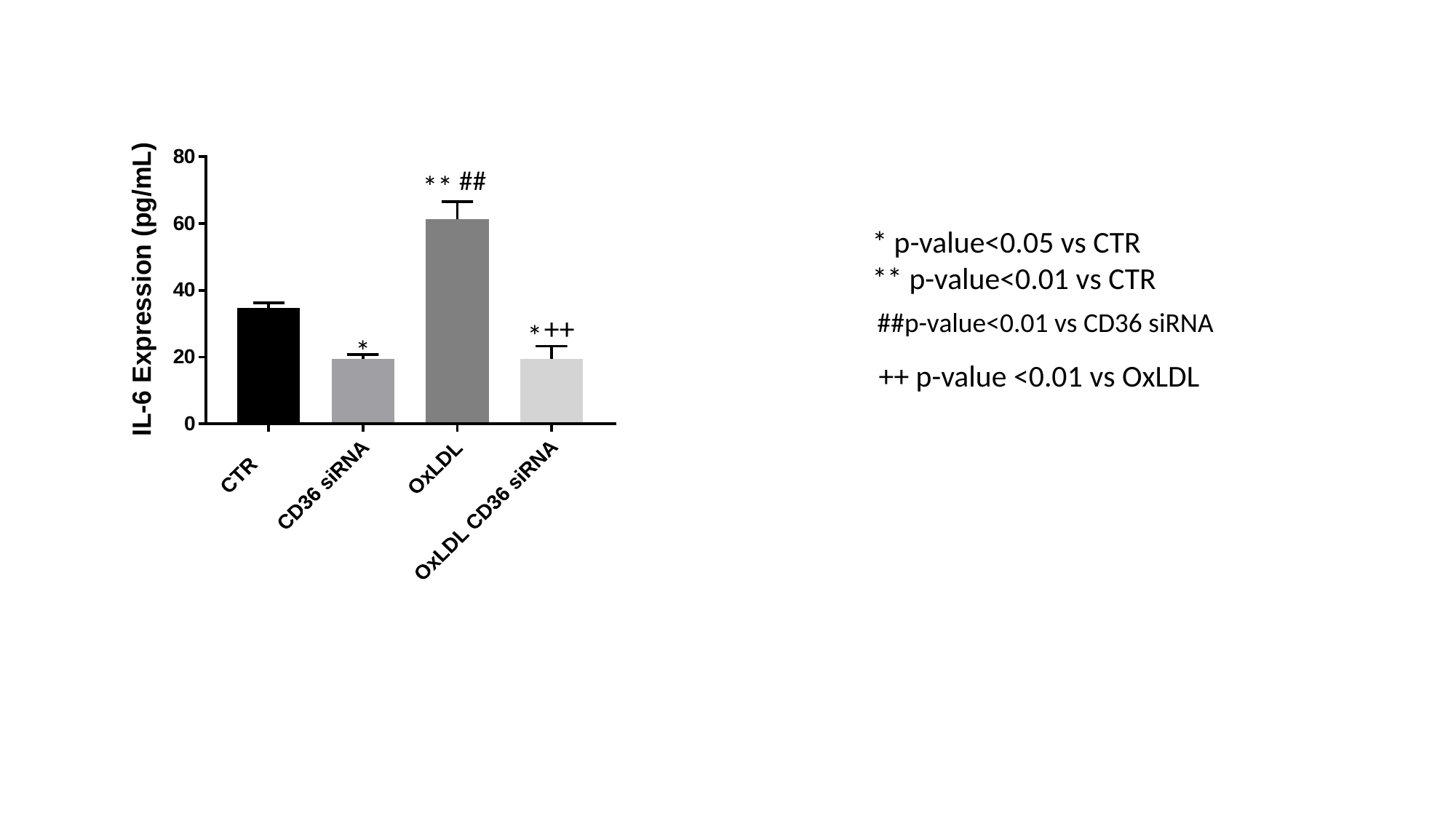

##
**
* p-value<0.05 vs CTR
** p-value<0.01 vs CTR
##p-value<0.01 vs CD36 siRNA
++
*
*
++ p-value <0.01 vs OxLDL

Supplement: Supplementary file 3 [file Datasheet3.zip › Figure 3D IL6/Il6 elisa cd36 sirna.pptx]

## Slide 1
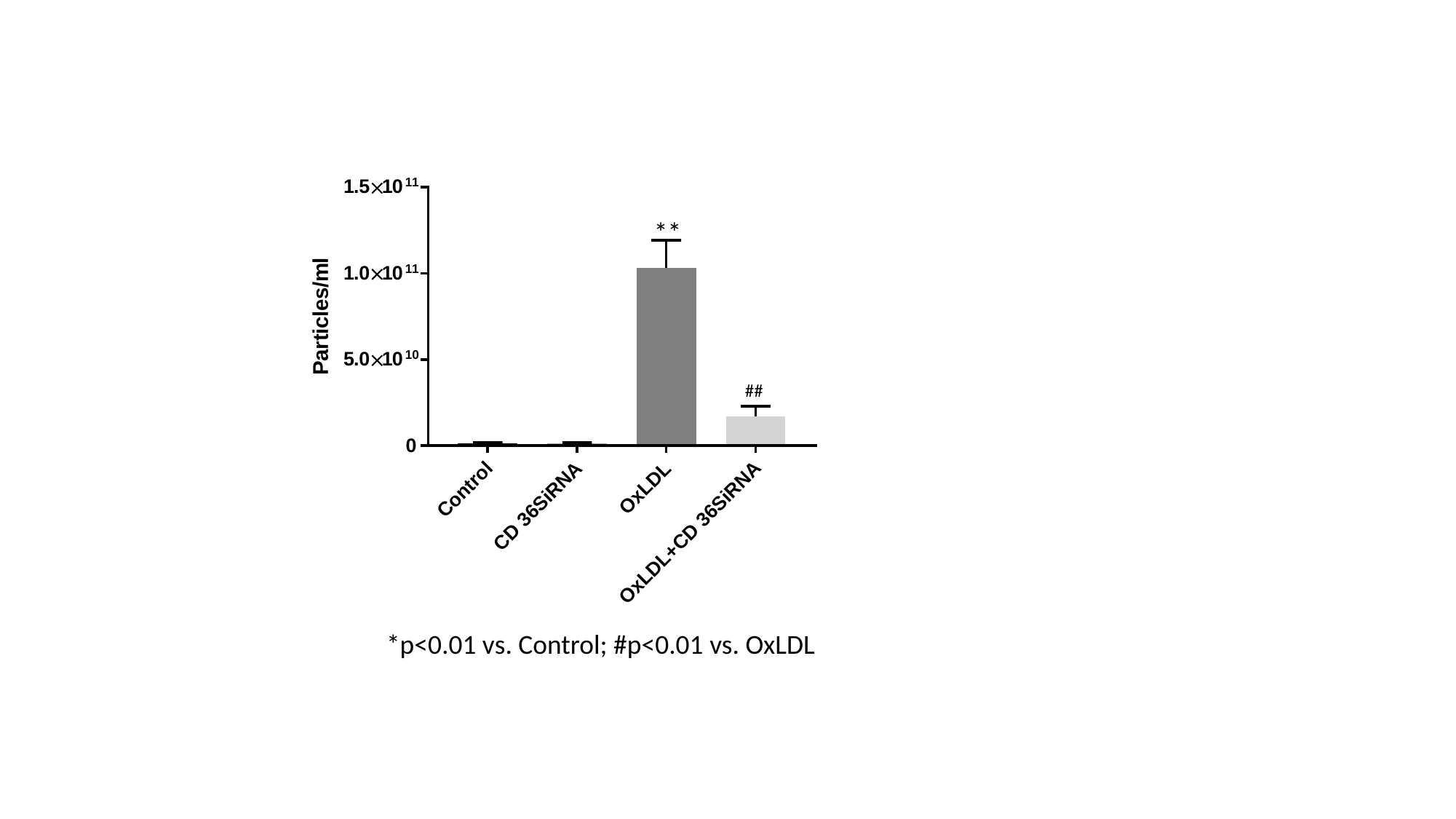

**
##
*p<0.01 vs. Control; #p<0.01 vs. OxLDL

Supplement: Supplementary file 3 [file Datasheet3.zip › Figure 3E/NTA-Exosome-3T3L1-CD 36 SiRNA Treatment/NTA-Exosome 3T3L1-CD 36 SiRNA Treatment.pptx]

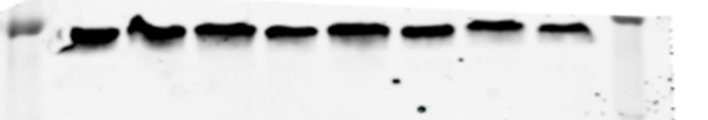

Supplement: Supplementary file 3 [file Datasheet3.zip › WB CD36/GAPDH Gel-1.tif]

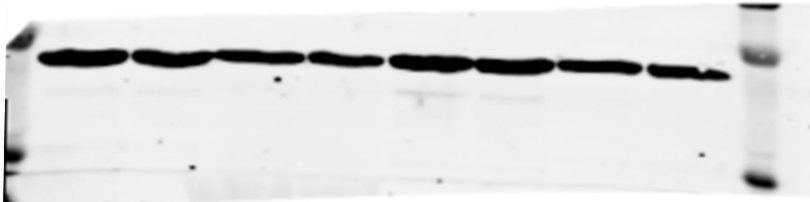

Supplement: Supplementary file 3 [file Datasheet3.zip › WB CD36/GAPDH Gel-2.tif]

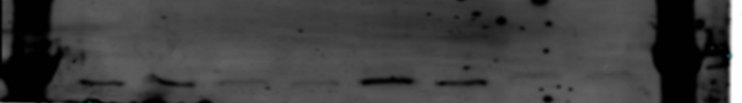

Supplement: Supplementary file 3 [file Datasheet3.zip › WB CD36/CD36 Gel-1.tif]

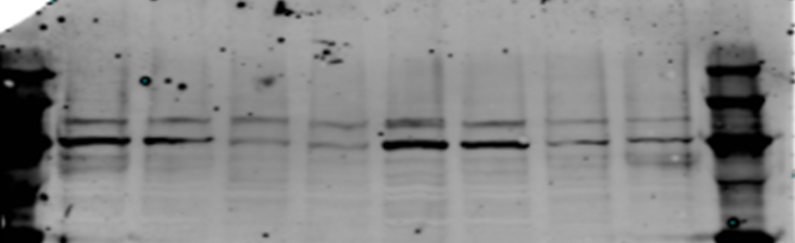

Supplement: Supplementary file 3 [file Datasheet3.zip › WB CD36/CD36 Gel-2.tif]

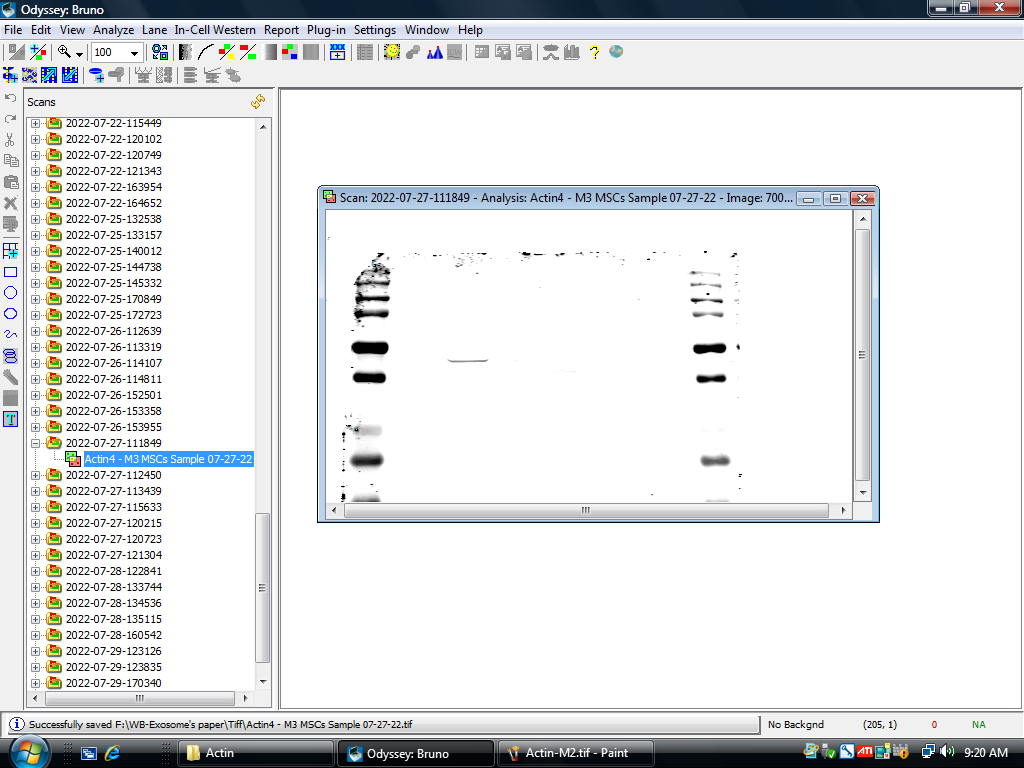

Supplement: Supplementary file 5 [file Datasheet5.zip › WB-MSC adipocytes Exosomes/Raw images/Actin/Actin-M3.tif]

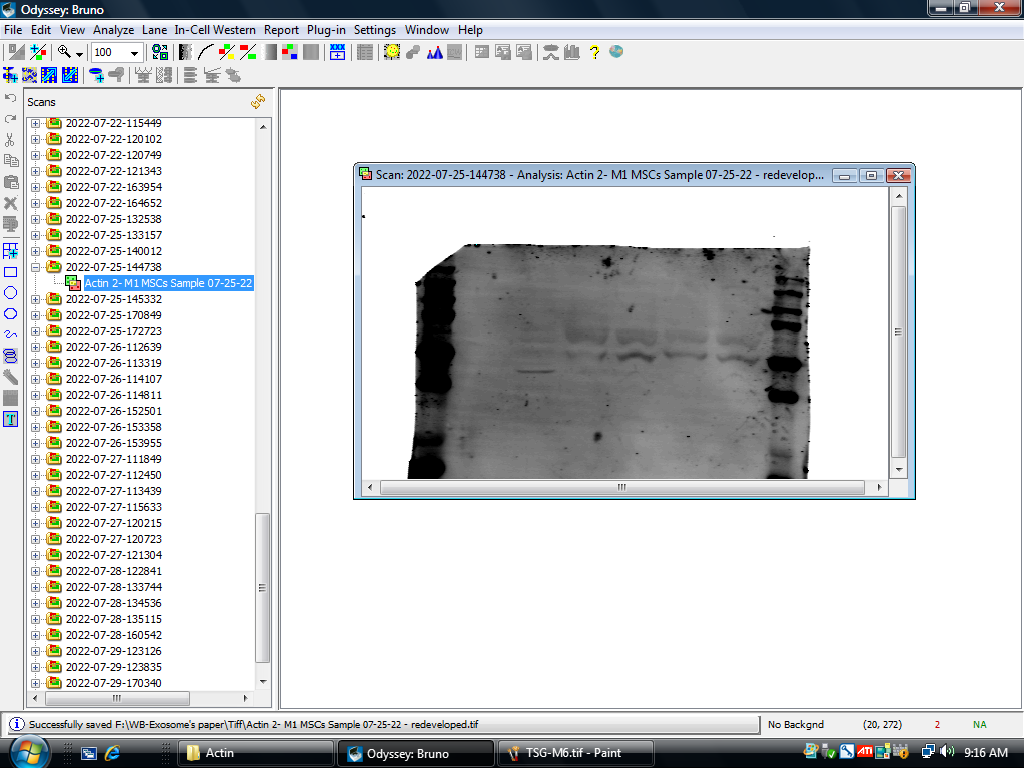

Supplement: Supplementary file 5 [file Datasheet5.zip › WB-MSC adipocytes Exosomes/Raw images/Actin/Actin-M1.tif]

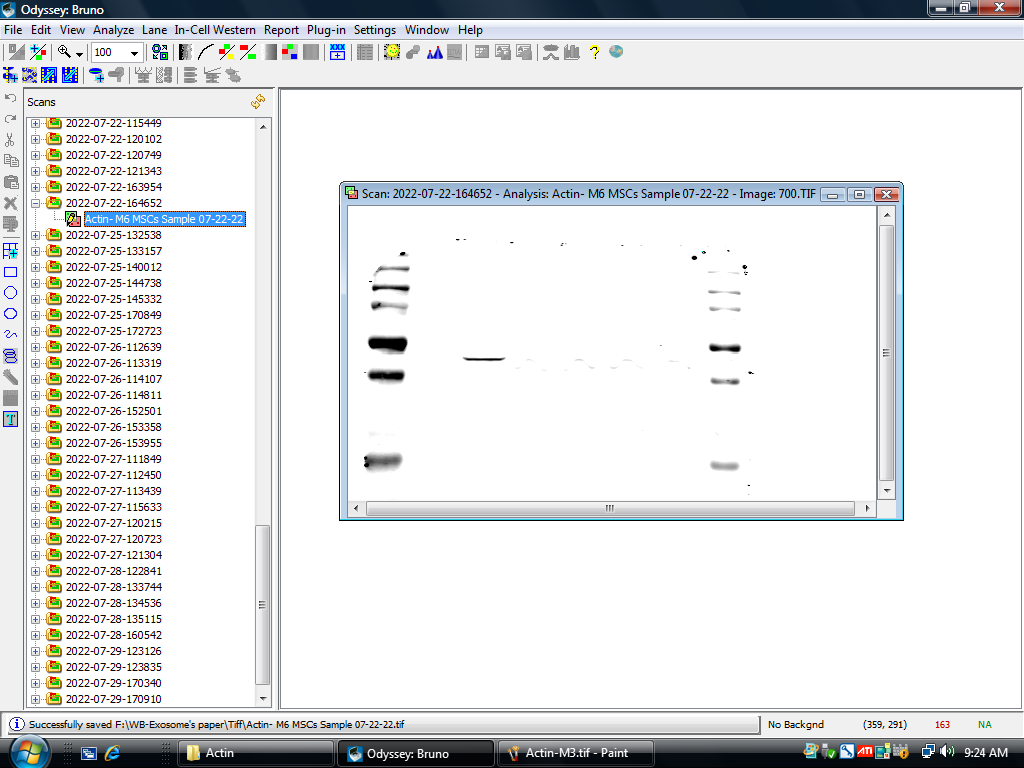

Supplement: Supplementary file 5 [file Datasheet5.zip › WB-MSC adipocytes Exosomes/Raw images/Actin/Actin-M6.tif]

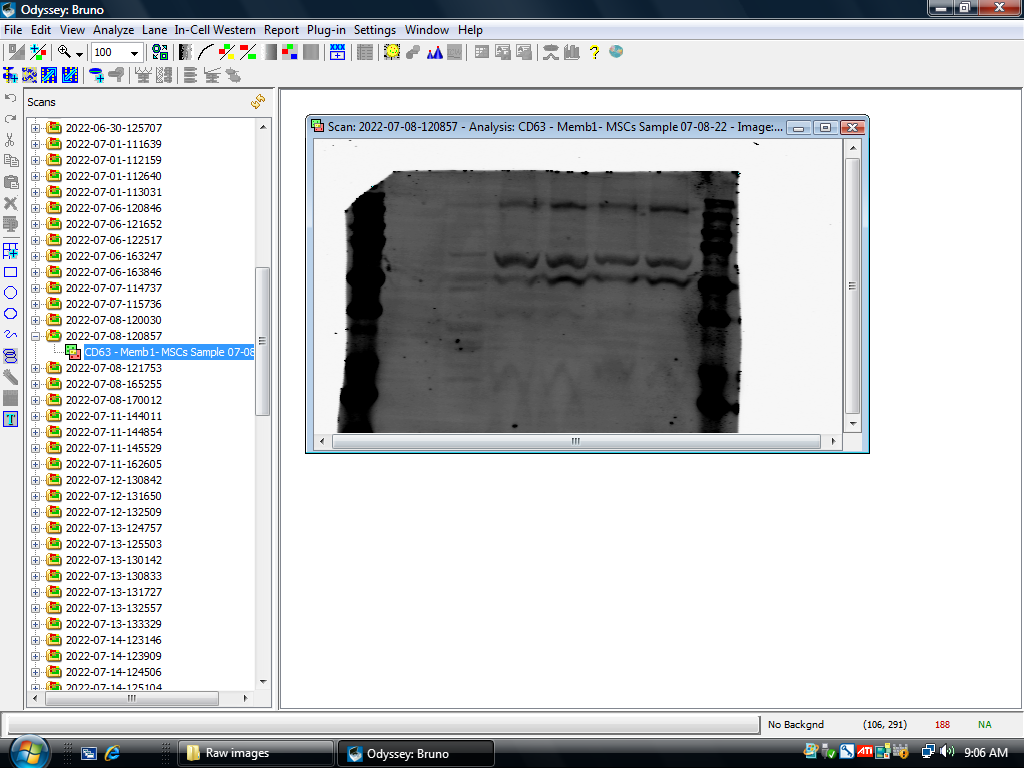

Supplement: Supplementary file 5 [file Datasheet5.zip › WB-MSC adipocytes Exosomes/Raw images/CD63/CD63-M1tif.tif]

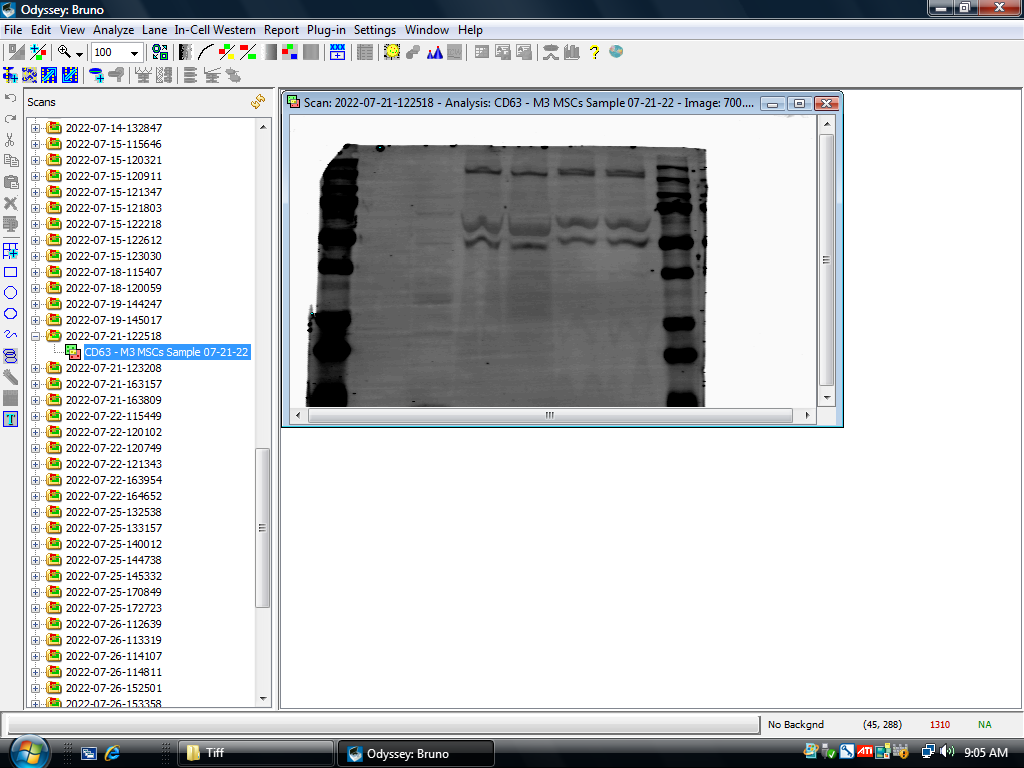

Supplement: Supplementary file 5 [file Datasheet5.zip › WB-MSC adipocytes Exosomes/Raw images/CD63/CD63-M3.tif]

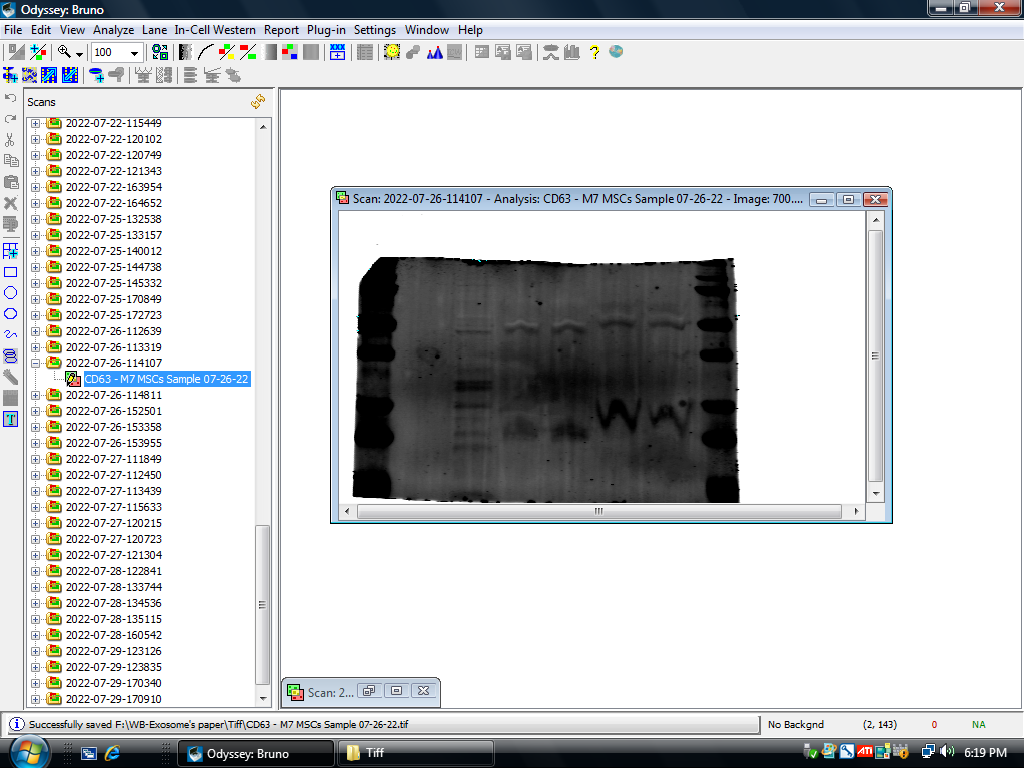

Supplement: Supplementary file 5 [file Datasheet5.zip › WB-MSC adipocytes Exosomes/Raw images/CD63/CD63-M7.tif]

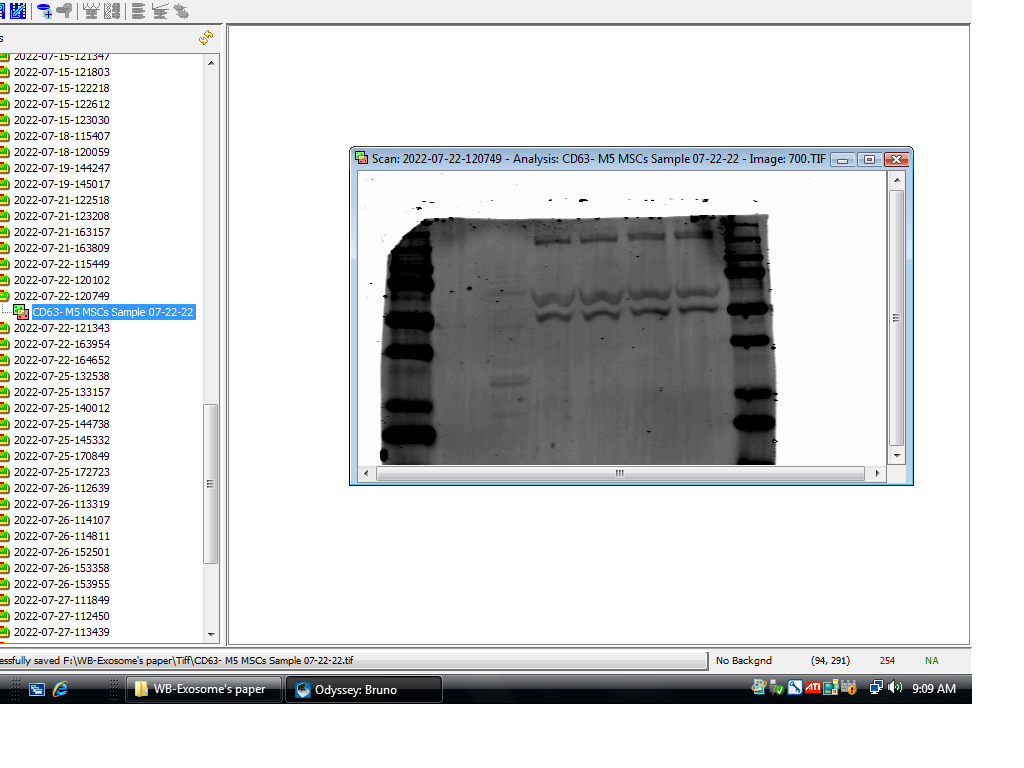

Supplement: Supplementary file 5 [file Datasheet5.zip › WB-MSC adipocytes Exosomes/Raw images/CD63/CD63-M5.tif]

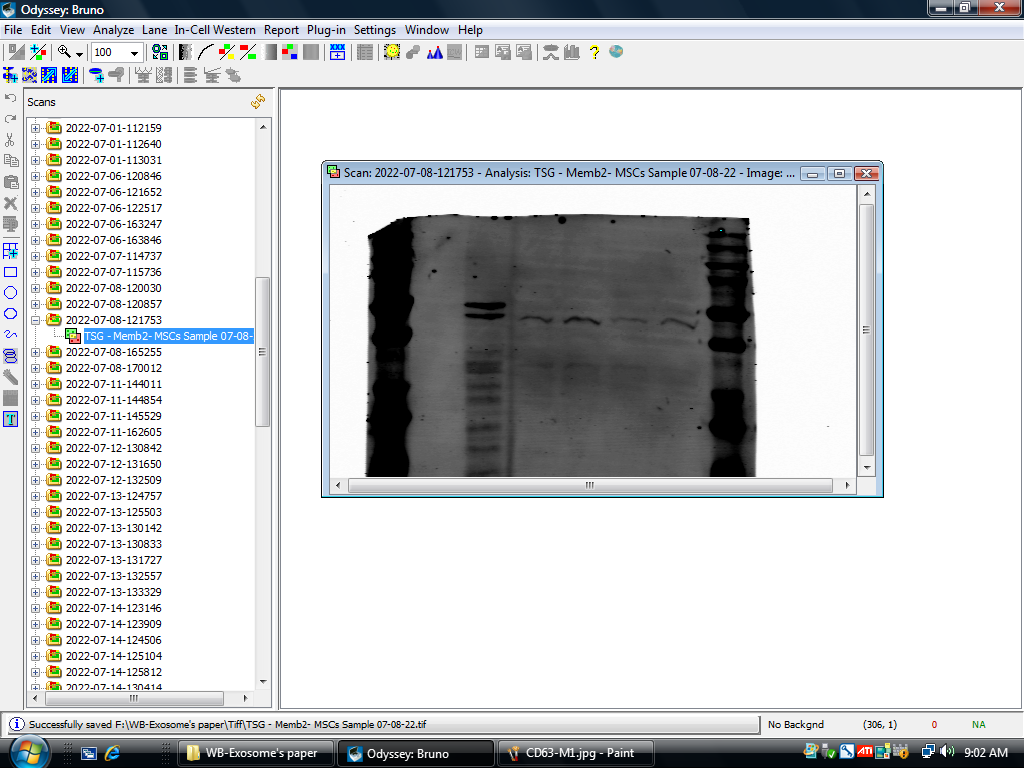

Supplement: Supplementary file 5 [file Datasheet5.zip › WB-MSC adipocytes Exosomes/Raw images/TSG/TSG-M2.tif]

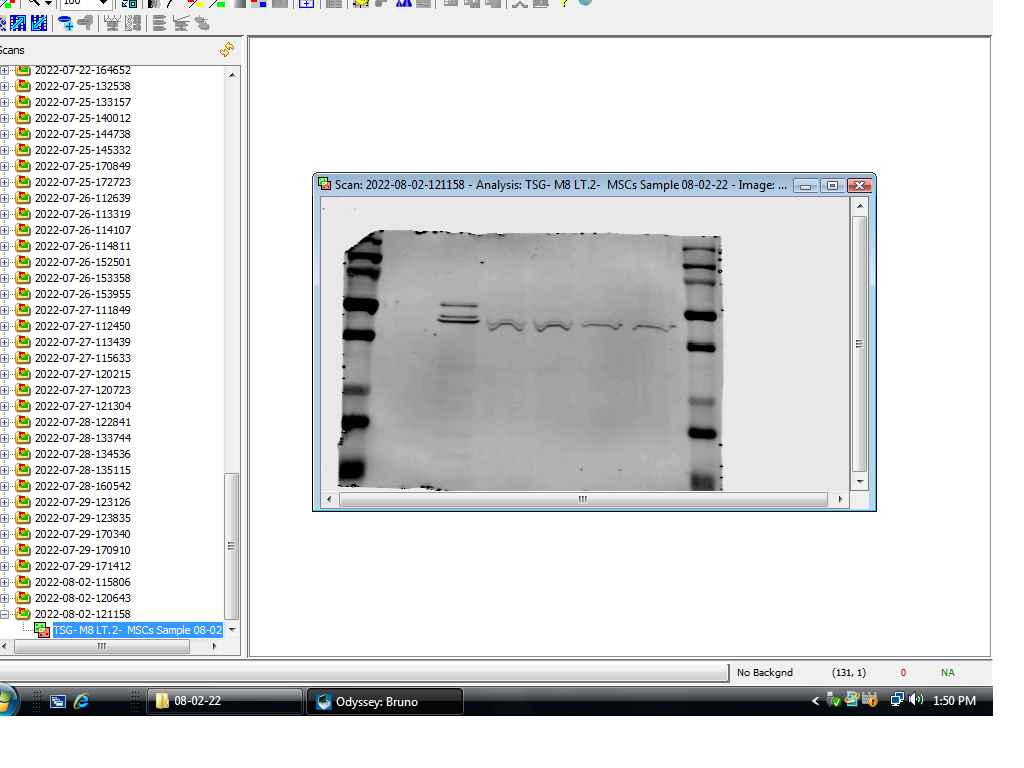

Supplement: Supplementary file 5 [file Datasheet5.zip › WB-MSC adipocytes Exosomes/Raw images/TSG/TSGM8.tif]

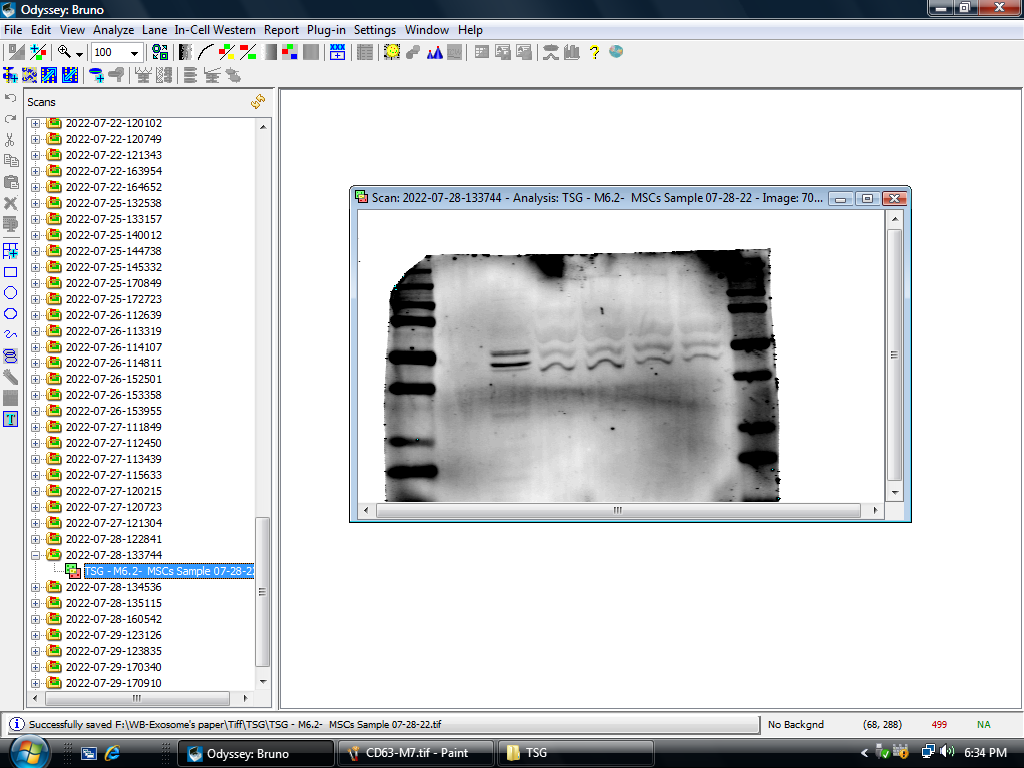

Supplement: Supplementary file 5 [file Datasheet5.zip › WB-MSC adipocytes Exosomes/Raw images/TSG/TSG-M6.2.tif]
