# Supplementary material for: Contribution of adipocyte Na/K-ATPase α1/CD36 signaling induced exosome secretion in response to oxidized LDL
Source: Front Cardiovasc Med. 2023 Apr 27;10:1046495. doi: 10.3389/fcvm.2023.1046495 (PMC10174328; doi:10.3389/fcvm.2023.1046495)
Supplement: Supplementary file 3 [file Datasheet3.zip › WB CD36/CD36 Blot 1.pptx]

## Slide 1
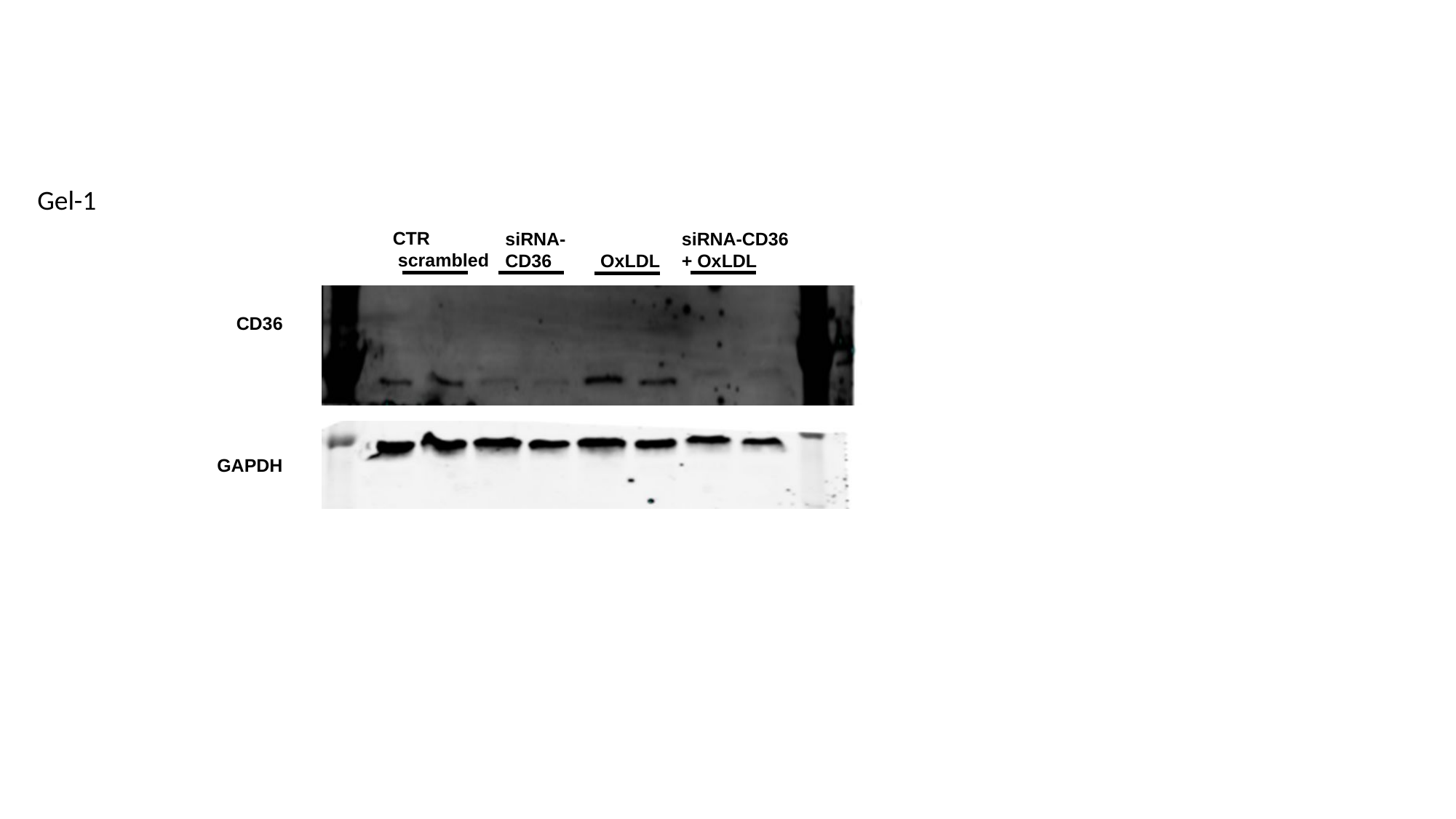

Gel-1
CTR
 scrambled
siRNA-
CD36
siRNA-CD36
+ OxLDL
OxLDL
CD36
GAPDH

## Slide 2
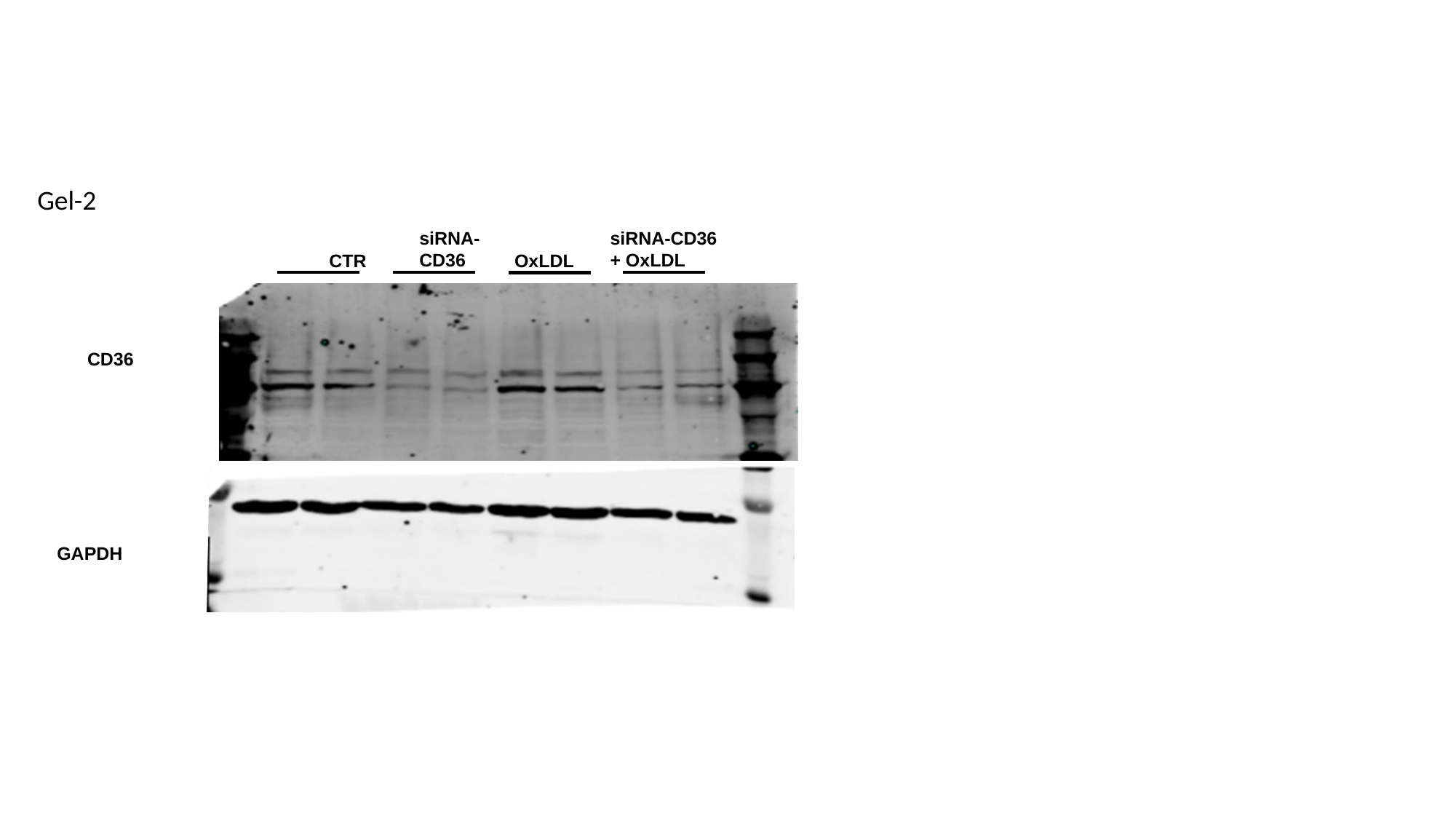

Gel-2
siRNA-
CD36
siRNA-CD36
+ OxLDL
CTR
OxLDL
CD36
GAPDH

## Slide 3
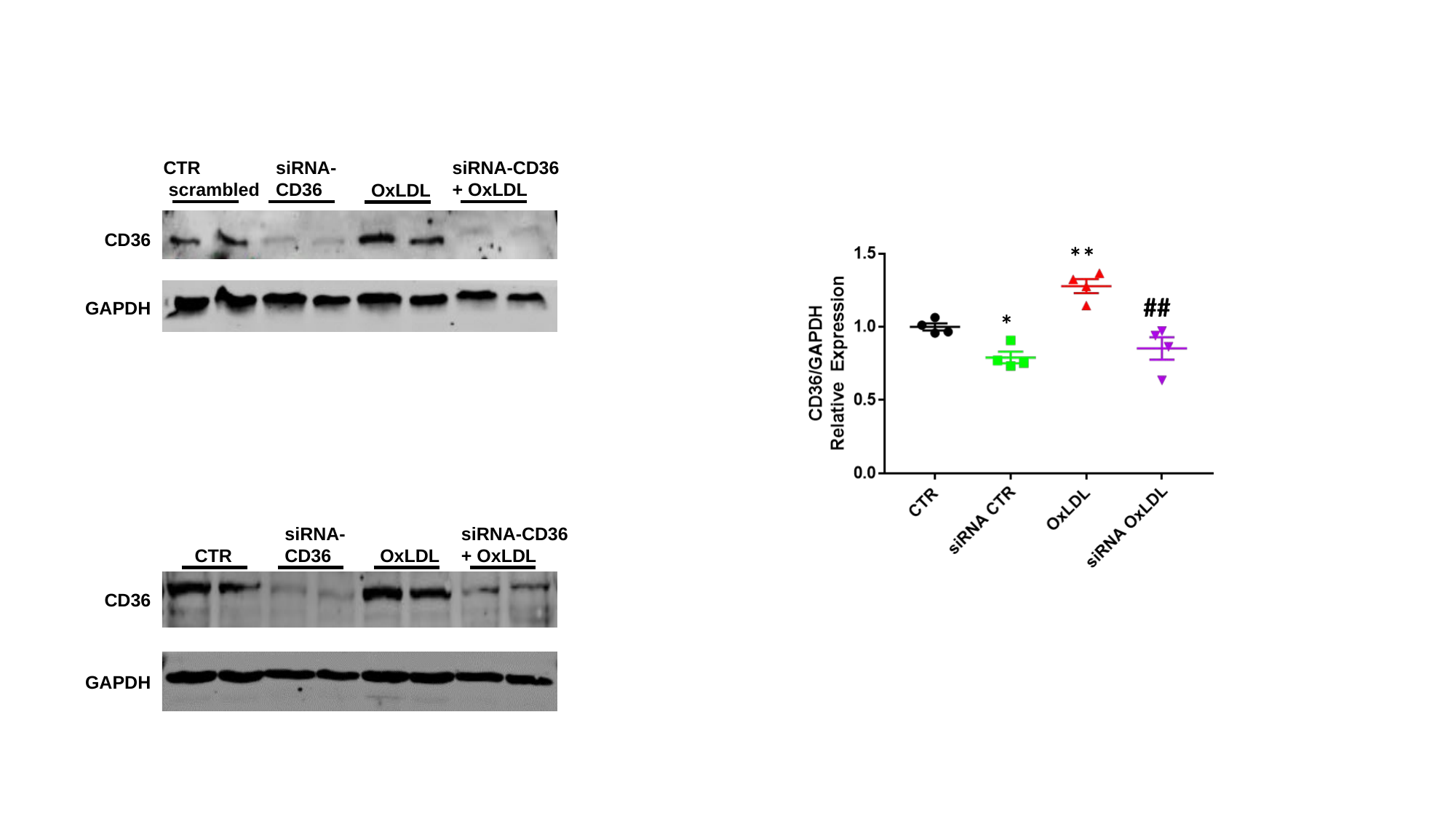

CTR
 scrambled
siRNA-
CD36
siRNA-CD36
+ OxLDL
OxLDL
CD36
**
##
GAPDH
*
siRNA-
CD36
siRNA-CD36
+ OxLDL
CTR
OxLDL
CD36
GAPDH
