# Supplementary material for: Contribution of adipocyte Na/K-ATPase α1/CD36 signaling induced exosome secretion in response to oxidized LDL
Source: Front Cardiovasc Med. 2023 Apr 27;10:1046495. doi: 10.3389/fcvm.2023.1046495 (PMC10174328; doi:10.3389/fcvm.2023.1046495)
Supplement: Supplementary file 5 [file Datasheet5.zip › WB-MSC adipocytes Exosomes/Final Adipocytes derived from MSCs-Results.pptx]

## Slide 1
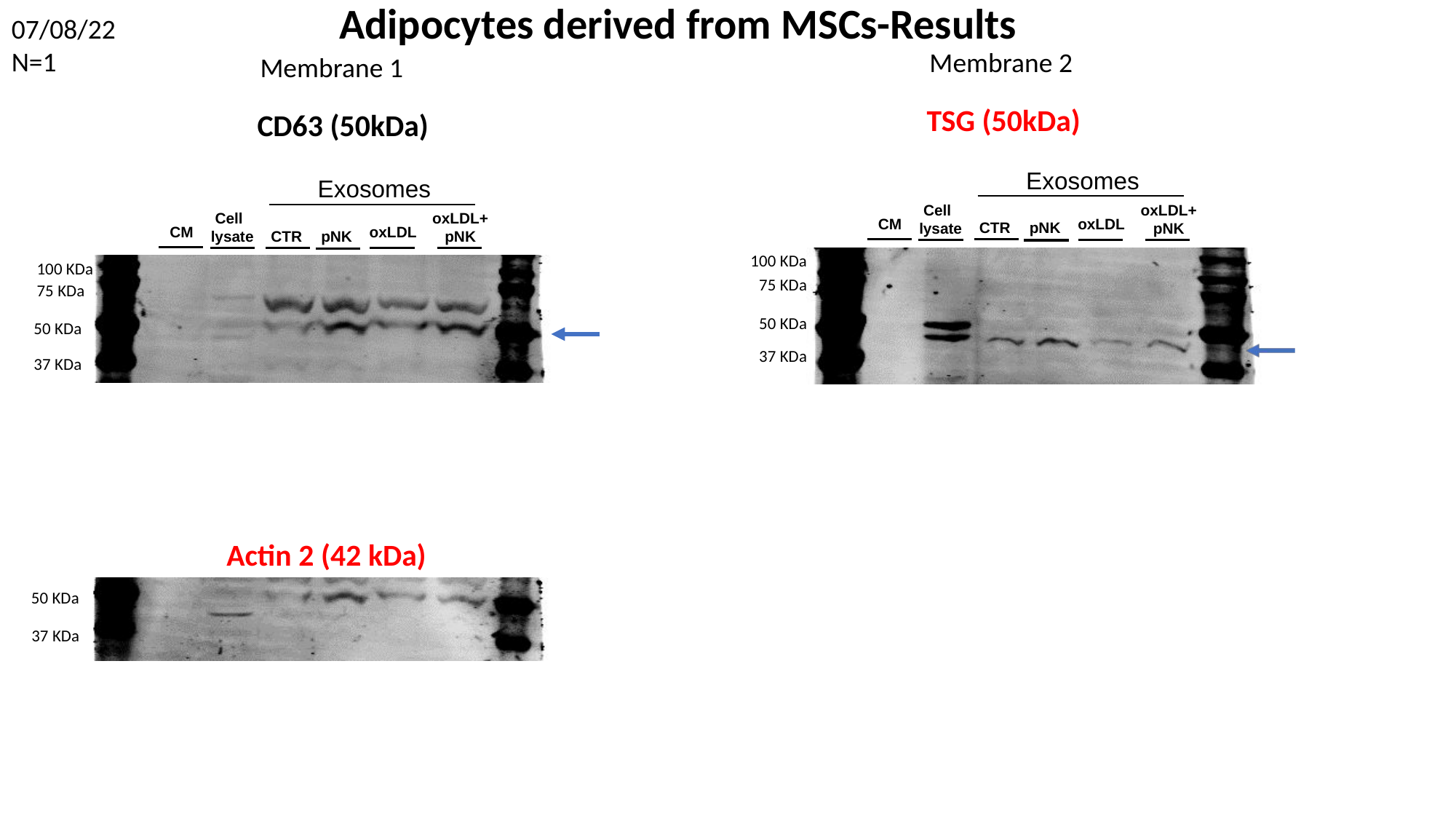

Adipocytes derived from MSCs-Results
07/08/22
N=1
Membrane 2
Membrane 1
TSG (50kDa)
CD63 (50kDa)
Exosomes
Exosomes
 Cell
lysate
oxLDL+pNK
 Cell
lysate
oxLDL+pNK
CM
oxLDL
pNK
CTR
CM
oxLDL
pNK
CTR
100 KDa
100 KDa
75 KDa
75 KDa
50 KDa
50 KDa
37 KDa
37 KDa
Actin 2 (42 kDa)
50 KDa
37 KDa

## Slide 2
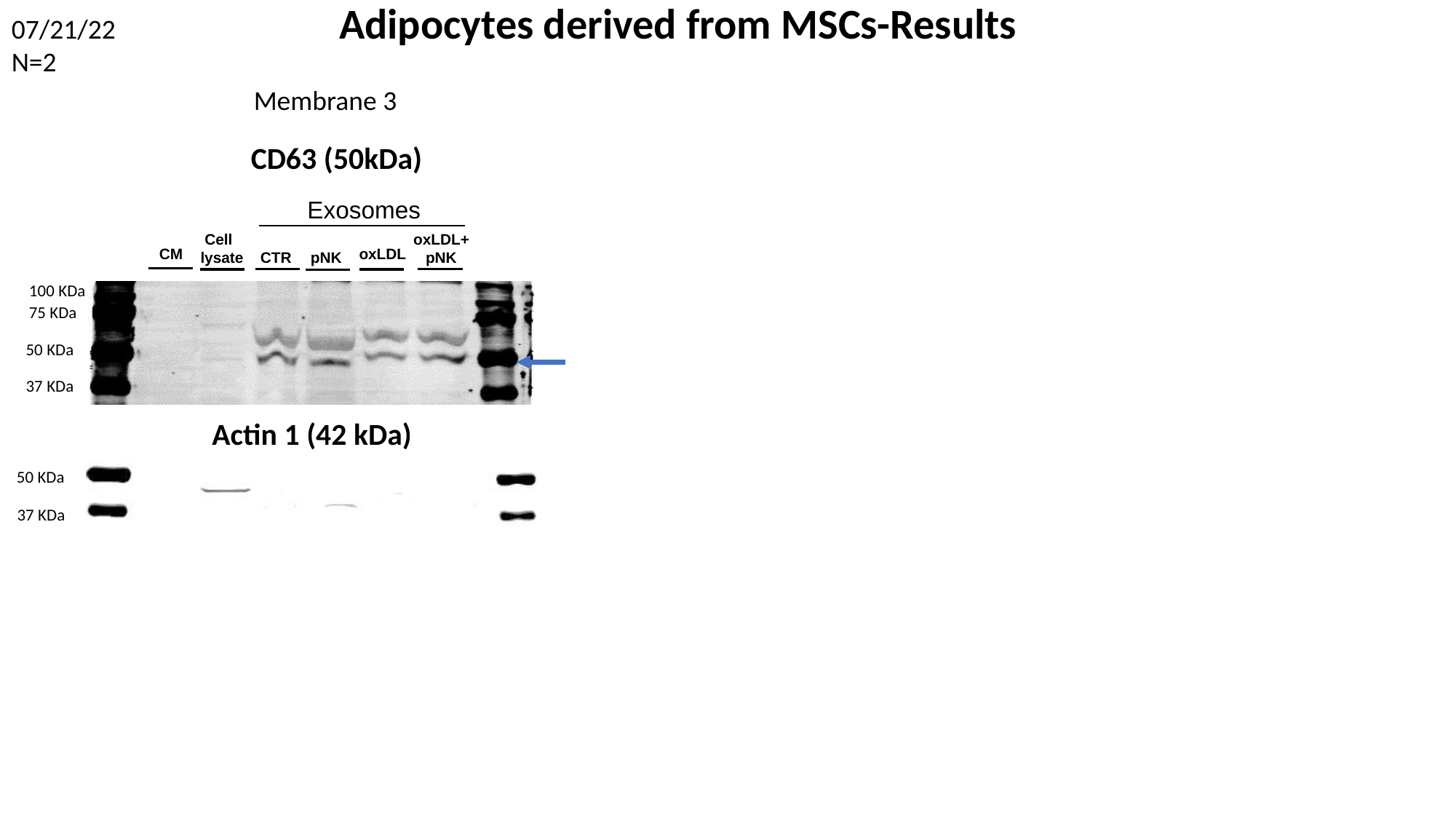

Adipocytes derived from MSCs-Results
07/21/22
N=2
Membrane 3
CD63 (50kDa)
Exosomes
 Cell
lysate
oxLDL+pNK
CM
oxLDL
pNK
CTR
100 KDa
75 KDa
50 KDa
37 KDa
Actin 1 (42 kDa)
50 KDa
37 KDa

## Slide 3
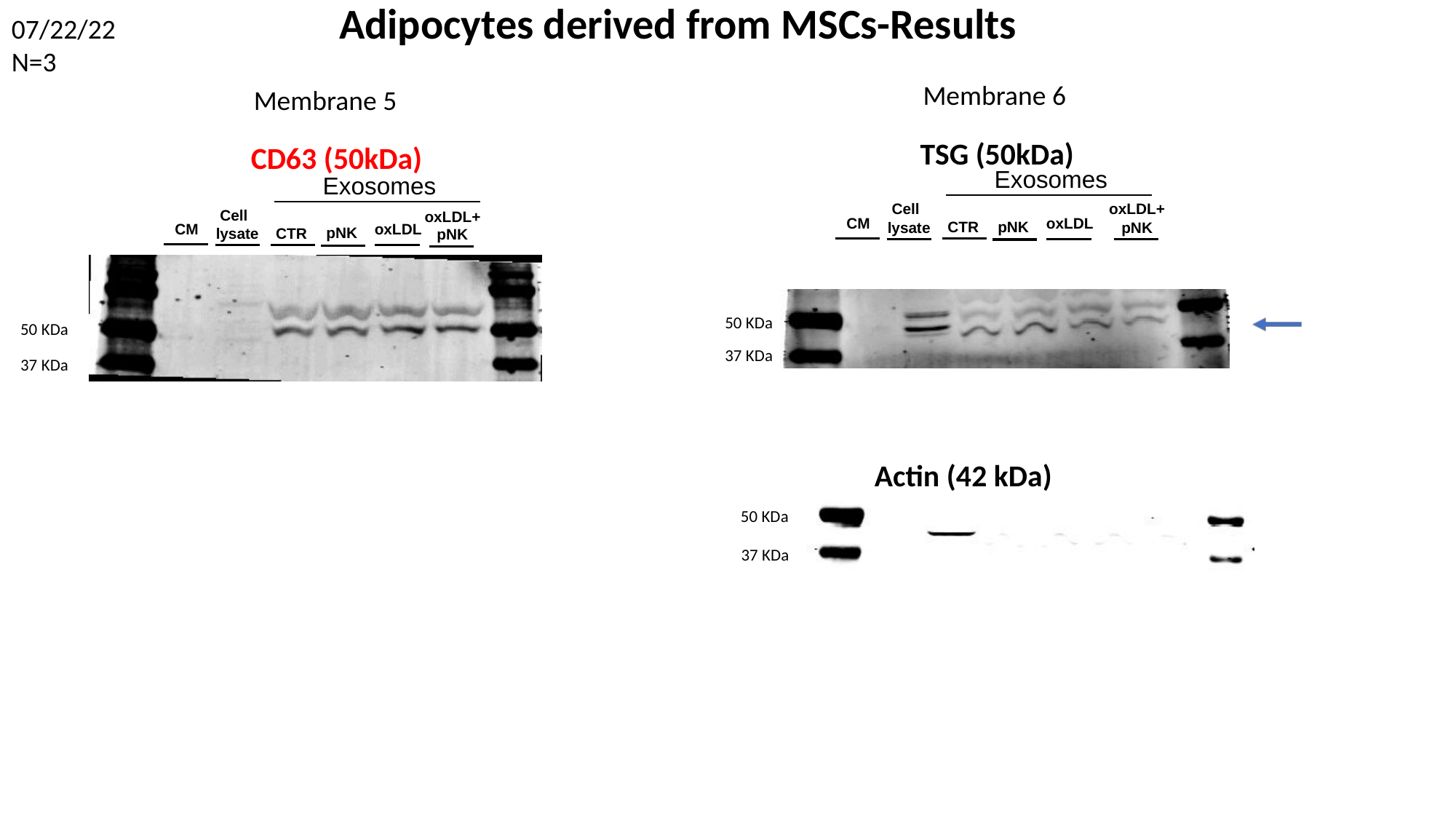

Adipocytes derived from MSCs-Results
07/22/22
N=3
Membrane 6
Membrane 5
TSG (50kDa)
CD63 (50kDa)
Exosomes
Exosomes
 Cell
lysate
oxLDL+pNK
 Cell
lysate
oxLDL+pNK
CM
oxLDL
pNK
CTR
CM
oxLDL
pNK
CTR
50 KDa
50 KDa
37 KDa
37 KDa
Actin (42 kDa)
50 KDa
37 KDa

## Slide 4
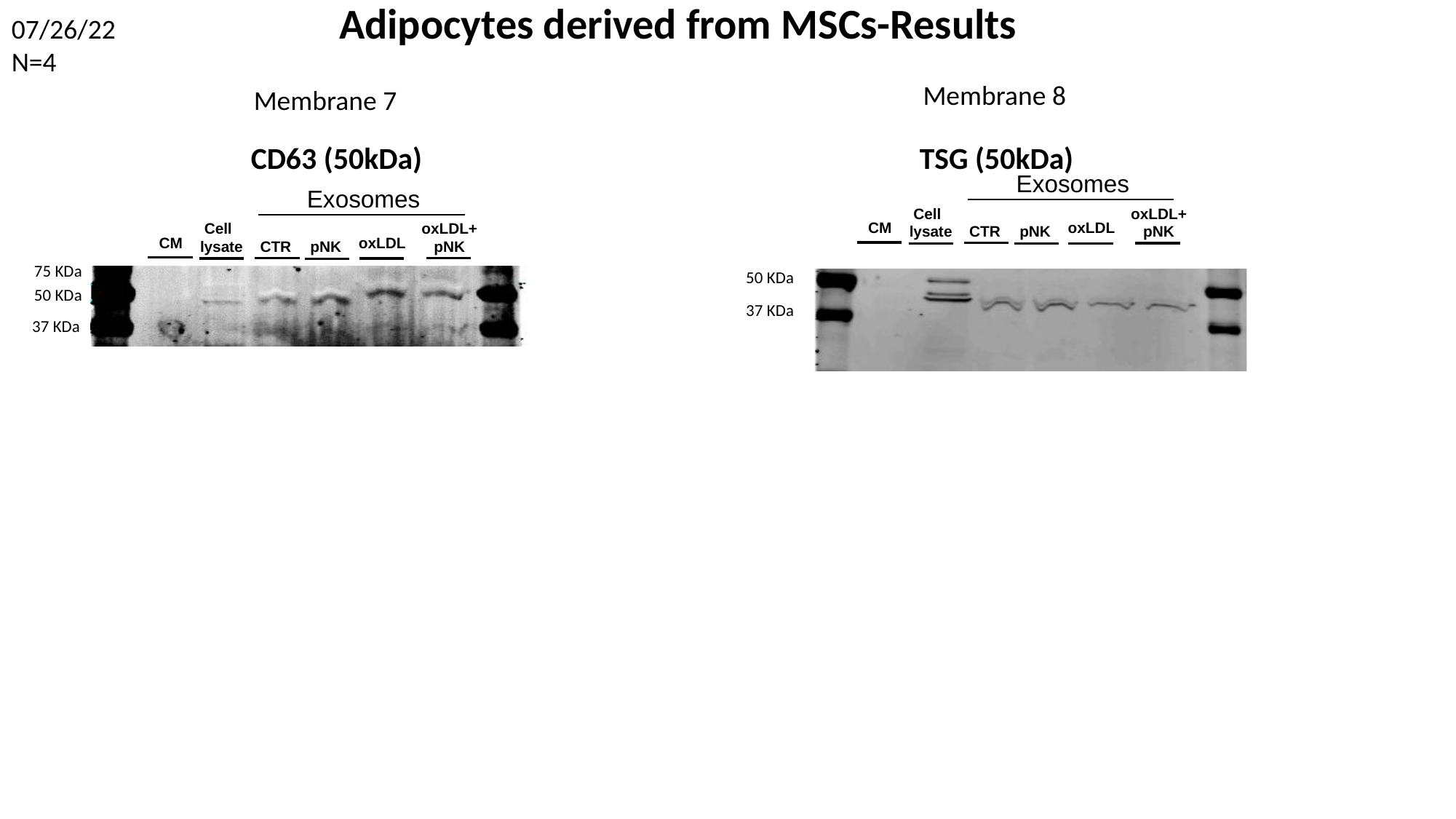

Adipocytes derived from MSCs-Results
07/26/22
N=4
Membrane 8
Membrane 7
CD63 (50kDa)
TSG (50kDa)
Exosomes
Exosomes
 Cell
lysate
oxLDL+pNK
CM
oxLDL
 Cell
lysate
oxLDL+pNK
pNK
CTR
CM
oxLDL
pNK
CTR
75 KDa
50 KDa
50 KDa
37 KDa
37 KDa
