# Supplementary material for: Contribution of adipocyte Na/K-ATPase α1/CD36 signaling induced exosome secretion in response to oxidized LDL
Source: Front Cardiovasc Med. 2023 Apr 27;10:1046495. doi: 10.3389/fcvm.2023.1046495 (PMC10174328; doi:10.3389/fcvm.2023.1046495)
Supplement: Supplementary file 6 [file Datasheet6.docx]

Supplementary Material

## Supplementary Figures

**Supplementary Figure 1.** **EMSC from WT and CD36 knockout (CD36^-/-^) mice can differentiate into adipocyte-like cells.** (**A**) EMSCs from WT and CD36^-/-^ mice at day 0, 7, and 9 post-differentiation were stained with Oil Red O and imaged at 20X magnification. Representative images were selected from three separate experiments. (**B**) Protein expression of PPARγ and tubulin was measured by immunoblot in WT and CD36^-/-^ EMSCs at day 0, 7, and 9 post-differentiation. Tubulin served as a loading control. Representative images were selected from three separate experiments. (**C**) Differentiated WT and CD36^-/-^ adipocytes were incubated with [9,10-^3^H(N)]-palmitic acid conjugated to fatty acid free BSA (4:1) for 5, 15, and 30 min. Radioactivity in the media and isolated lipids was counted and a percent palmitic acid uptake was calculated (n=3, performed in triplicate). Values represent mean ± SEM. *** p<0.001, **** p < 0.0001 WT vs. CD36^-/-^.

**
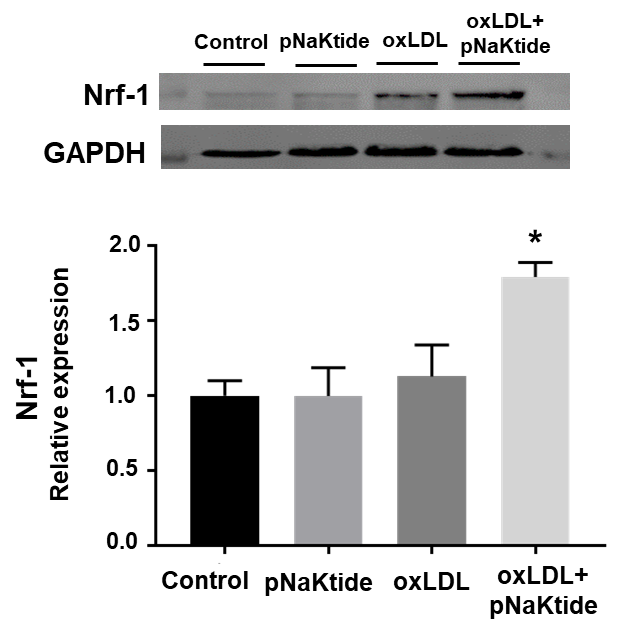
**

**Supplementary Figure 2.** **The effect of oxLDL treatment on Nrf-1 protein expression in 3T3-L1 murine adipocytes.** Differentiated 3T3-L1 adipocytes were treated with 50 µg/ml oxLDL and/or 0.7 µM pNaKtide every 24 h for 2 days. Immunoblot analysis was performed for Nrf-1. Representative blot images are shown, and data are represented as mean band density normalized to GAPDH (n=5). Values represent mean ± SEM.* p < 0.05 vs. oxLDL.
